# Zirconium-Based Metal–Organic Framework Mixed-Matrix Membranes as Analytical Devices for the Trace Analysis of Complex Cosmetic Samples in the Assessment of Their Personal Care Product Content

**DOI:** 10.1021/acsami.1c21284

**Published:** 2022-01-10

**Authors:** Adrián Gutiérrez-Serpa, Tanay Kundu, Jorge Pasán, Ana I. Jiménez-Abizanda, Stefan Kaskel, Irena Senkovska, Verónica Pino

**Affiliations:** † Laboratorio de Materiales para Análisis Químicos (MAT4ALL), Departamento de Química, Unidad Departamental de Química Analítica, Universidad de La Laguna (ULL), 38206 La Laguna, Tenerife, Spain; ‡ Unidad de Investigación de Bioanalítica y Medioambiente, Instituto Universitario de Enfermedades Tropicales y Salud Pública de Canarias, Universidad de La Laguna (ULL), 38206 La Laguna, Tenerife, Spain; § Department of Chemistry, SRM Institute of Science and Technology, Kattankulathur, 603203 Chennai, Tamil Nadu, India; ∥ Technische Universität Dresden (TUD), Bergstrasse 66, 01069 Dresden, Germany; ⊥ Laboratorio de Materiales para Análisis Químicos (MAT4ALL), Departamento de Química, Unidad Departamental de Química Inorgánica, Universidad de La Laguna (ULL), 38206 La Laguna, Tenerife, Spain

**Keywords:** mixed-matrix membranes, metal−organic frameworks, polymeric composite, personal care products, environmental analyses, isotherms

## Abstract

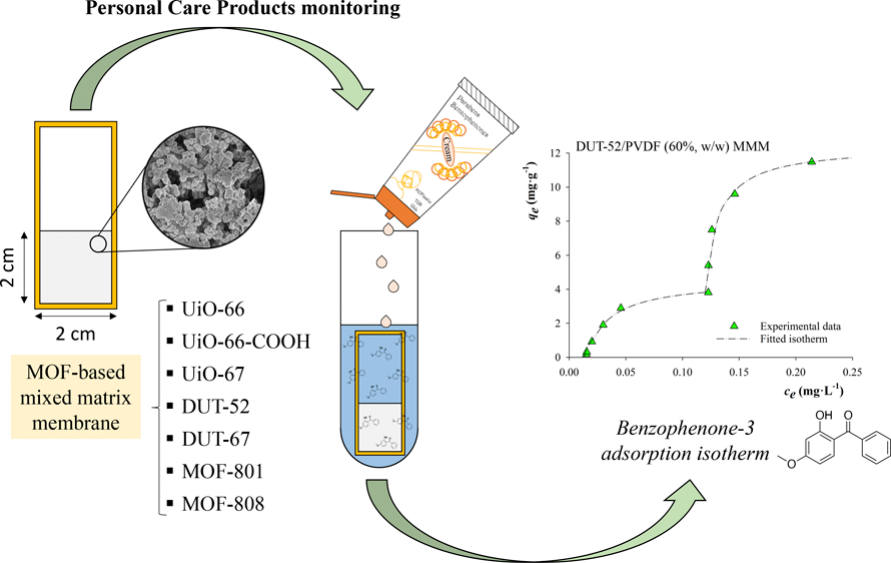

A device comprising a zirconium-based metal–organic framework (MOF) mixed-matrix membrane (MMM) framed in a plastic holder has been used to monitor the content of personal care products (PCPs) in cosmetic samples. Seven different devices containing the porous frameworks UiO-66, UiO-66-COOH, UiO-67, DUT-52, DUT-67, MOF-801, and MOF-808 in polyvinylidene fluoride (PVDF) membranes were studied. Optimized membranes reach high adsorption capacities of PCPs, up to 12.5 mg·g^–1^ benzophenone in a 3.0 mg·L^–1^ sample. The MMM adsorption kinetics, uptake measurements, and isotherm studies were carried out with aqueous standard solutions of PCPs to ensure complete characterization of the performance. The studies demonstrate the high applicability and selectivity of the composites prepared, highlighting the performance of PVDF/DUT-52 MMM that poses uptakes up to 78% for those PCPs with higher affinity while observing detection limits for the entire method down to 0.03 μg·L^–1^. The PVDF/DUT-52 device allowed the detection of parabens and benzophenones in the samples, with PCPs found at concentrations of 1.9–24 mg·L^–1^.

## Introduction

The design of novel devices in the analytical sample preparation aims to simplify their use while keeping both high extraction rates of target compounds from complex sample matrices, and high desorption rates in compatible solvents with the subsequent analytical instrument for detection.^1^ Highly robust and chemically stable materials are required in such devices, together with other valuable aspects linked to the performance of the device, such as uniformity and reproducibility.^2^

Metal–organic frameworks (MOFs) are highly porous crystalline materials characterized for having one of the highest surface areas known so far.^3^ MOFs are made of two basic secondary building units (SBUs): (i) metal ions (or metal clusters) that act as nodes and (ii) organic molecules as linkers. The SBUs form an ordered network containing regular pores and channels via coordination bonds. The large number of possible combinations of metal ions and organic linkers makes these materials more versatile than other porous materials. In addition, further modifications can be introduced to their structure after their synthesis.^4,5^ Due to these properties, the use of MOFs has become popular in various fields such as gas storage, water treatment, and biomedicine.^4,6,7^

The use of neat MOF membranes is challenging in practical applications, as their preparation is difficult and expensive.^8^ Thus, they have been integrated in mixed-matrix membranes (MMMs) which are composites characterized for combining a polymer as a matrix continuous phase, and an inorganic or inorganic–organic material denoted as a filler or additive.^9^ The intrinsic structural properties of the resulting membrane, such as the permeability, are defined by the nature of the polymer, while the filler defines the adsorptive properties. In the case of using MOFs as the filler, MMMs can be prepared by different approaches including blending, in situ growth, layer-by-layer (LBL), and gelatin-assisted seed growth.^2^ Among them, the preparation of MOF-based MMMs by blending is advantageous due to its simplicity. Blending methods can be done by three different strategies depending on how the colloidal suspension of the filler/polymer mixture is combined. It is possible by the following: (i) dispersing the filler in a solvent and adding the polymer, (ii) dissolving the polymer and adding the filler, or (iii) dissolving the polymer and dispersing the filler, separately, and then mixing them, forming a homogeneous colloidal suspension termed “pre-ink” or “ink”. The third option ensures the homogeneous integration of the MOF into the polymeric matrix, reducing the possible agglomeration of the MOFs.^2^

Analytical applications require MOFs with high stability in aqueous media, such as aluminum-, chromium-, and zirconium-based MOFs; that is, MIL-53(Al), MIL-101(Cr), and UiO-66(Zr).^4^ The stability of Zr-based MOFs together with their impressive surface area has made these materials highly attractive for their use in the development of devices for analytical microextraction. In this sense, miniaturized devices containing MOFs have been presented in different formats, shapes, and sizes, including solid-phase microextraction fibers, thin films made of MMMs, and stir bars, among others.^10^

Thin-film microextraction devices require an extractant material spread over a wide surface. By this strategy, the amount of extractive phase area exposed to a certain sample considerably increases if compared to conventional approaches. This permits to extract a larger amount of the target analyte and improving the sensitivity, without requiring larger extraction times (as it happens with conventional methods). So far, only one thin-film device made of polydimethylsiloxane (PDMS) is commercially available. This extractant polymeric phase lacks selectivity due to its nature.^11^ Besides, its thermal and chemical stability is not as good as desired. Thus, recent efforts are shifted to develop novel extractant phases to be used in thin-film microextraction approaches.^11^

Few MOF-based MMMs have been used as microextraction devices in analytical chemistry. The MOFs used as filler for these MMMs include UiO-66,^12^ UiO-66-NH_2_,^13^ MIL-53(Al),^14,15^ MIL-53-NO_2_(Al),^16^ and MIL-101(Cr).^17^ These MOF-based MMMs have been used for the determination of phenols,^13^ dyes,^12^ estrogens,^15^ H_2_S,^16^ drugs,^17^ and polycyclic aromatic hydrocarbons^14^ present in water, drinks, and urine. However, none of the reported applications studies the extraction behavior of the MOF-based MMMs, neither the adsorption capacity nor the extraction kinetics of the membranes, to fully characterize and/or understand the efficacy of these devices when used in complex samples.

This research article studies seven zirconium-based MOF MMMs as thin-film devices in analytical microextraction, thoroughly evaluating their capability for the adsorption of contaminants of emerging concern such as personal care products (PCPs) in complex aqueous media, including cosmetic formulations. This information serves to evaluate their use as a potential alternative to commercial polymeric membranes. The MOFs to prepare the MMMs were chosen based on their stability, easiness of synthesis, and low costs associated to their synthesis. Thus, UiO-66 was chosen, being not only a pioneer of Zr-MOFs but also one of the most water-stable MOFs. MOF-801, UiO-66-COOH, UiO-67, and DUT-52^18−21^ were also studied as they are isoreticular to UiO-66, making it possible to evaluate the effect of enlarging pore volume on the adsorption capacity. Besides, DUT-67 and MOF-808 were studied as an alternative to these MOFs, as their synthesis is performed in water, making the entire preparation procedure more sustainable.^22^ Apart from the impact of the MOF nature, other parameters affecting the performance of the membranes, such as MOF loading, kinetics, isotherms, and their possible applicability, were also evaluated.

## Results and Discussion

### MOF-Based MMM Preparation and Characterization

To prepare highly homogeneous and robust devices based on thin films with MMMs, several preparation parameters have to be optimized. These parameters include the dispersion method used for obtaining a homogeneous pre-ink, the solvent evaporation method during the ink preparation, the spreading speed, the amount of ink used, and the thickness of the MOF-based MMM. The results obtained with these studies are all available in detail in the Supporting Information. As main features, it is possible to obtain homogeneous and resistant membranes with a thickness of 60 μm when using a maximum MOF loading value of 60%.

The seven MOFs synthesized and all the MOF-based MMMs were characterized by powder X-ray diffraction (XRD), Fourier transform infrared (FT-IR) spectroscopy, N_2_ adsorption, thermogravimetric analysis (TGA), and scanning electron microscopy (SEM).

**Figure 1:**
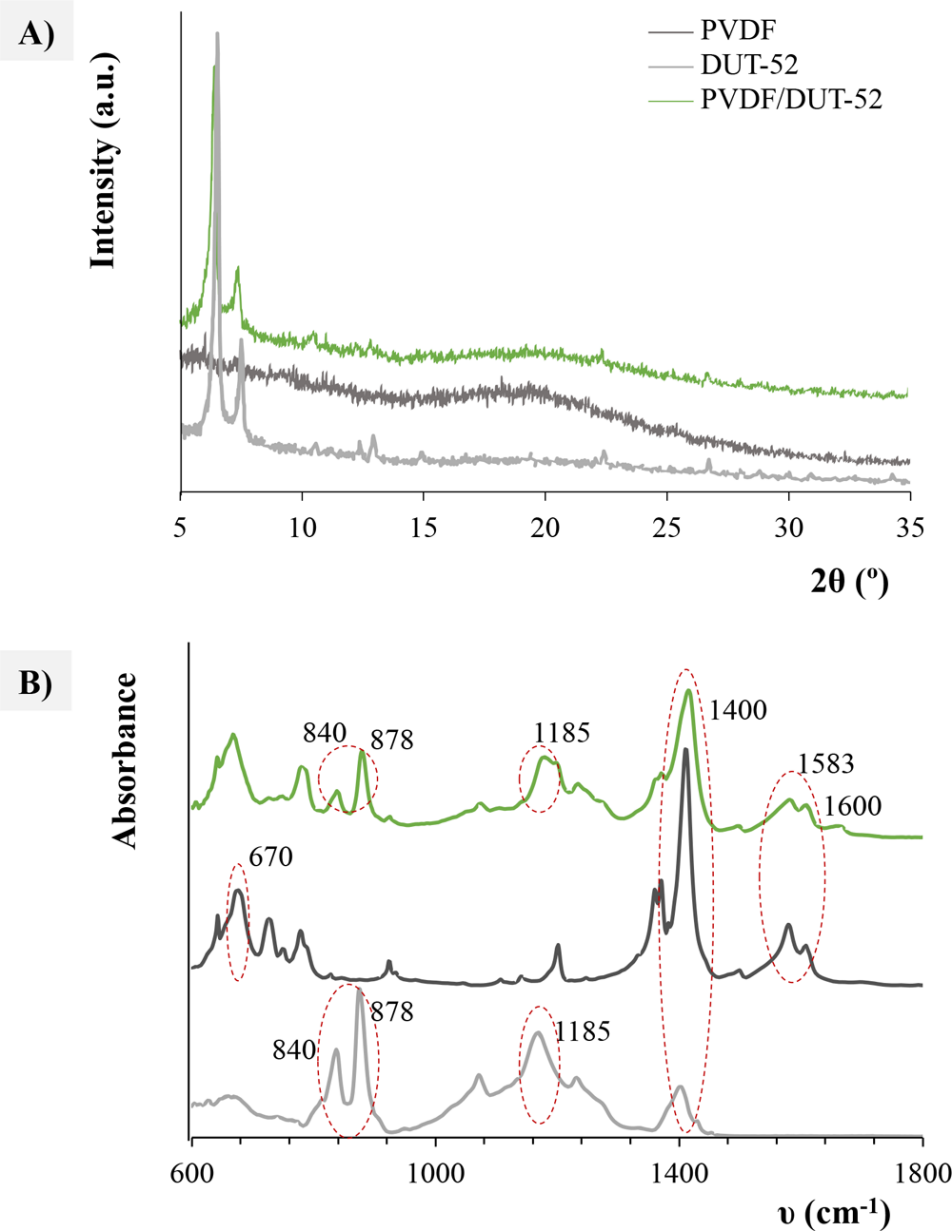
Representative examples of MOF(DUT-52)-based MMM characterization: (A) powder XRD patterns and (B) FT-IR spectra.

All the MOFs presented good crystallinity (Figures 1 and S5) and their PXRD patterns are in good agreement with the simulated ones, supporting that only the desired phase is present.^23^ Regarding the patterns of the MOF-based MMMs (Figure S6), they keep all the reflections from the neat MOF XRD patterns, and therefore, their crystal structure is maintained after being embedded into the polymeric matrix of polyvinylidene fluoride (PVDF). A broad background peak corresponding to the amorphous PVDF phase can be observed in all the cases.

The infrared spectrum of neat PVDF is shown in Figures 1B and S7, with characteristic peaks at 875 and 840 cm^–1^ related to C–C–C and CF_2_ asymmetric stretching vibration, respectively. Furthermore, peaks at 1180 cm^–1^ and at ∼1400 cm^–1^, related to the C–C and the CH_2_ wagging vibration bands, respectively, are observed.^24,25^ The neat MOF infrared spectra (Figure S7) show bands around 670 cm^–1^ assigned to the Zr-μ_3_-O stretching nodes. The bands located at 1400 cm^–1^ correspond to the C=O stretching, and those at 1593 cm^–1^ are due to the C=C stretching, typically observed with aromatic compounds. Figure S7 shows the presence of characteristic bands belonging to the polymer and the neat MOFs in the MOF-based MMMs.

**Figure 2:**
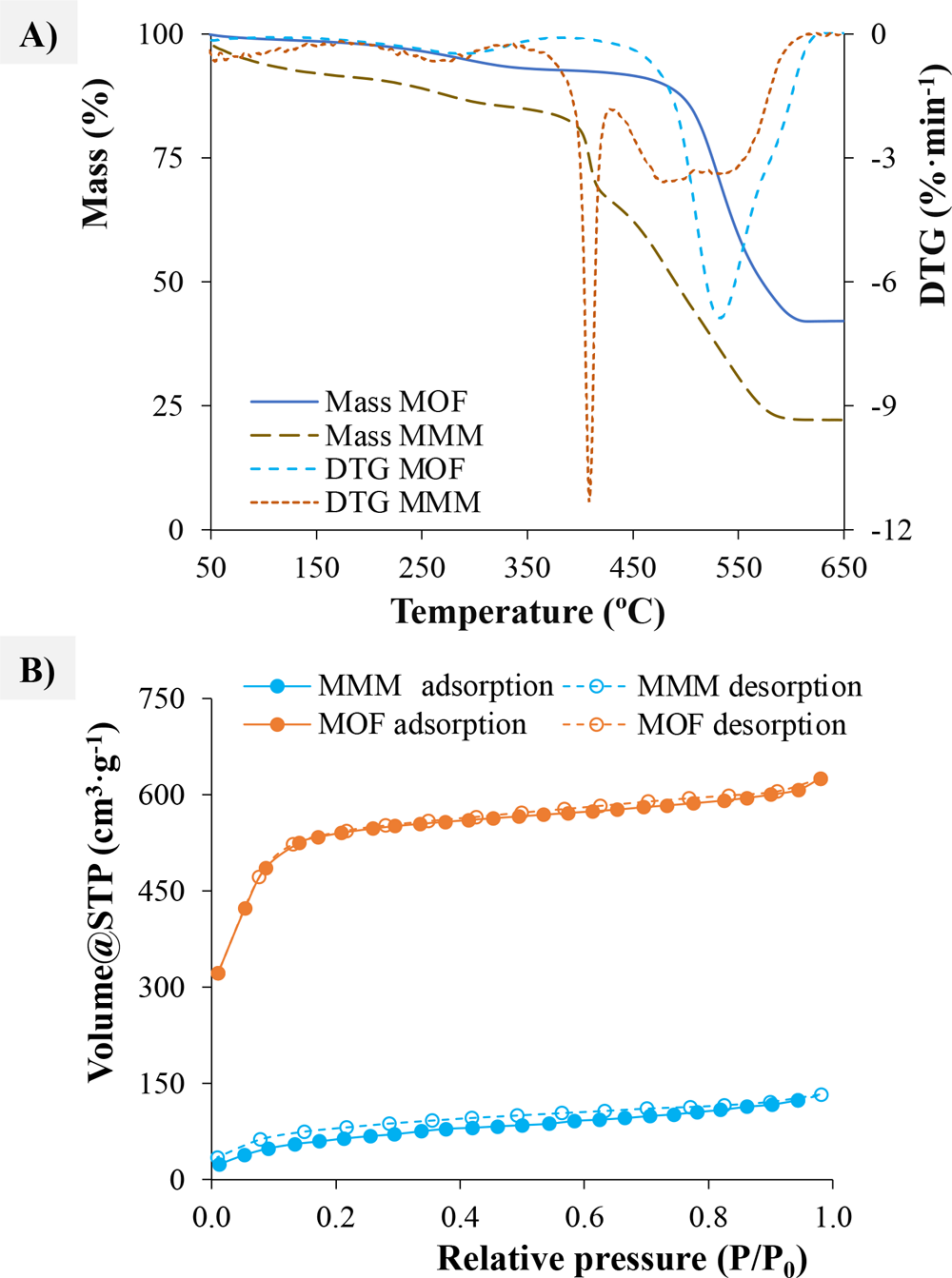
Representative examples of MOF(DUT-52)-based MMM characterization: (A) TGA and (B) N_2_ adsorption.

The thermal stability of PVDF, MOFs, and MOF-based MMMs was measured by TGA (Figures 2A and S8). For neat PVDF, it can be observed that there is a slight mass loss due to the removal of moisture, *N*,*N*′-dimethylformamide (DMF), and MeOH from 30 to 407 °C. The decomposition of the polymer starts at 407 °C, occurring in two steps. The first one, from 407 to 497 °C, corresponds to PVDF primary (main) degradation of the polymer, and then, there is a second decomposition step up to 697 °C, being the total amount of mass loss close to 100%. These values are in concordance with previous studies reported on the evaluation of thermal stability of PVDF.^25,26^ Regarding the thermal stability of the seven MOFs synthesized, it can be observed in all cases that a first step of mass loss, related to the removal of water and/or organic solvents trapped in the pores, is between 35 and 350 °C. The second step in the neat MOF TGA corresponds to their decomposition, which significantly varies from 310 to 480 °C (Table 1).

**Table 1:**
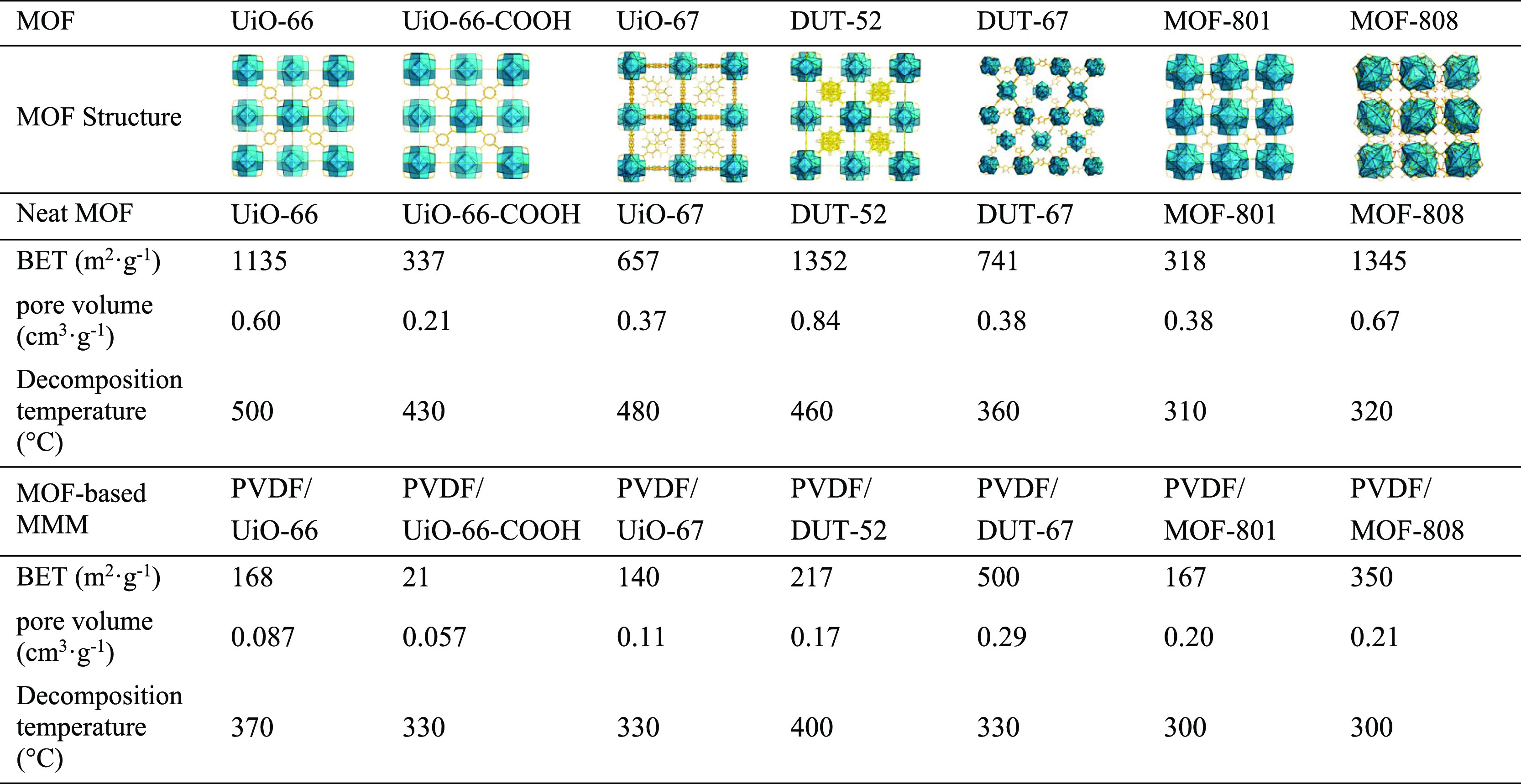
MOF Structure, Specific Surface Areas, Pore Sizes, and Decomposition Temperatures of Neat MOFs and MOF-Based MMMs (60%, w/w)

The decomposition temperature of the MOF-based MMMs is slightly lower than that of the neat MOF and the neat PVDF. Besides, it is also possible to observe the first mass loss associated to the polymer and then the decomposition of the MOF. A 40% of mass loss is registered during the polymer decomposition. This agrees with the prepared MMMs that were intended to be loaded with 60% of MOF and 40% of PVDF. Among the MOF-based MMMs studied, PVDF/DUT-52 MMM showed the highest thermal stability followed by PVDF/UiO-66 MMM.

The N_2_ adsorption capacity of the MOFs is in good accordance with the reported values in the literature (Figures 2B, S9, and S10). Among them, DUT-52 shows the highest adsorption capacity and the largest pore volume (Table 1). Thus, a better extraction capability can be expected for this MOF.

There is a considerable decrease on the gas adsorption for MMMs due to the permeability of the PVDF (Figure S9 and Table 1). Neat PVDF has a low adsorption capacity and a surface area of only 5.2 m^2^·g^–1^. However, good adsorption properties are measured when combining MOF and PVDF. Thus, MMMs loaded with DUT-67, MOF-808, and DUT-52 have higher extraction capacity than the other MOFs, being therefore UiO-66-COOH, UiO-66, UiO-67, and MOF-801, less favorable as analytical microextraction devices.

**Figure 3:**
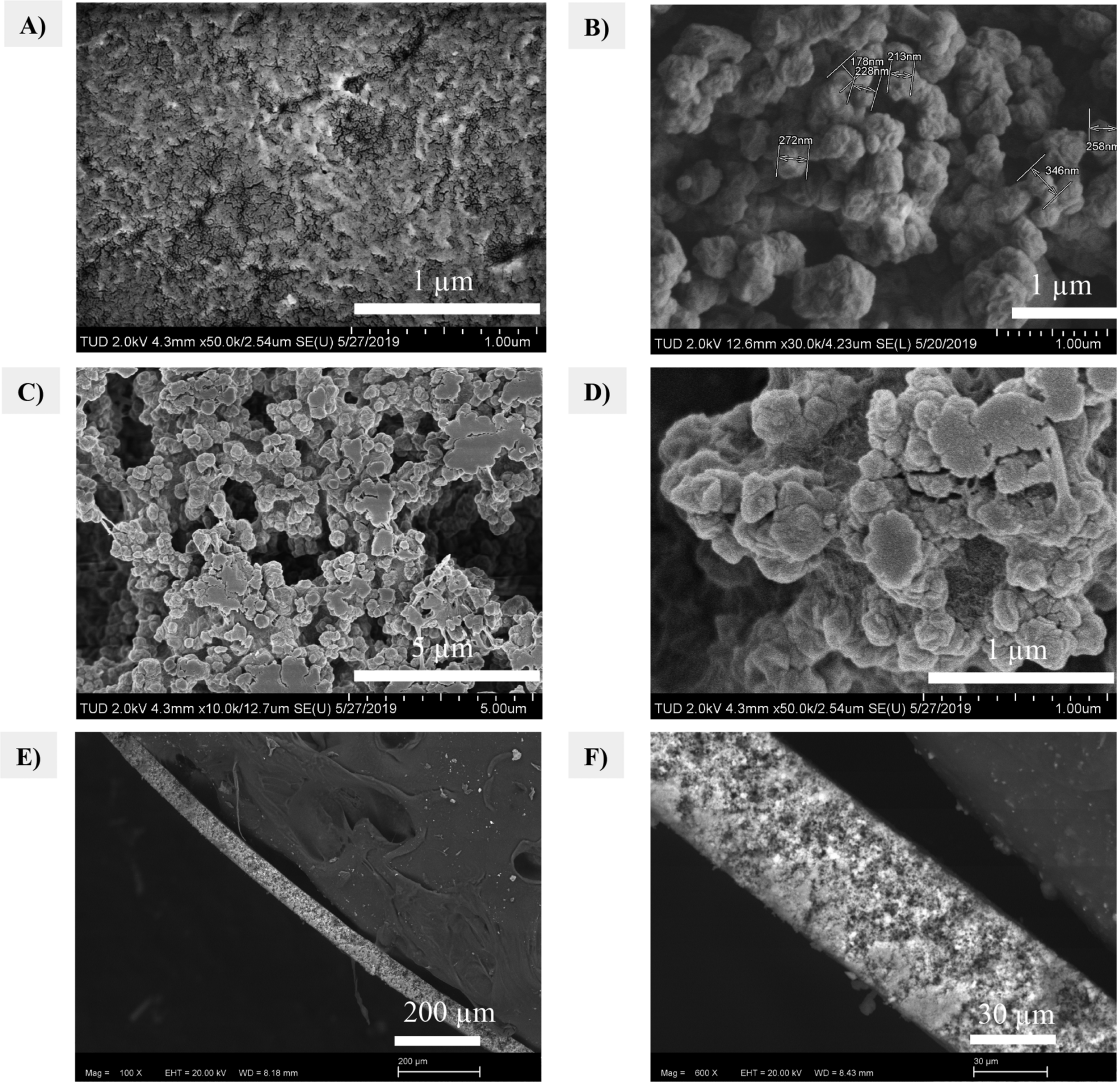
Representative examples of SEM images: (A) PVDF and (B) DUT-52, whereas PVDF/DUT-52 (60%, w/w) MMM is shown in (C–F).

The morphology of the neat PVDF membrane shows a rough surface with cracks (Figures 3A and S11A). SEM images of the MOF-based MMMs show the clear integration of the MOF in the polymeric matrix, while the MOF crystallites kept their morphology (Figure 3C,D). For those MOFs with small crystal sizes, such as UiO-66-COOH and UiO-66 (Figures 3B and S11B,C), a higher degree of agglomeration and less uniformity on the MOF-polymer packing can be observed, whereas for those MOFs with bigger crystallites, such as DUT-67 and MOF-808, the MOF particles are well dispersed in the polymer (Figure S11F–H). In the cross-sectional SEM image, it is possible to observe a thickness of 60 μm for the MOF-based MMMs (Figure 3E,F).

### Adsorption Studies

**Figure 4:**
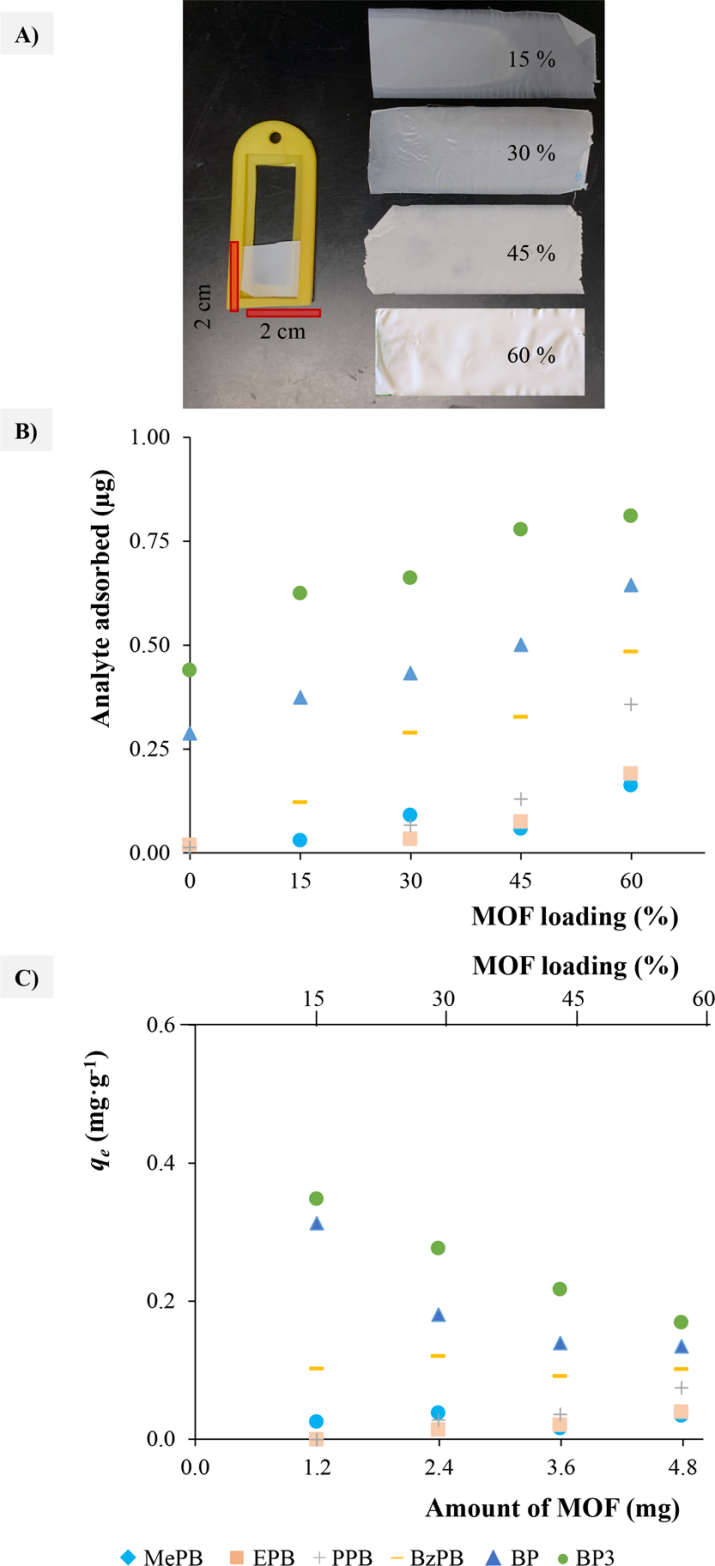
(A) Device used for the adsorption studies, the membrane at different MOF loadings (in %), and experiments performed for the characterization of the membrane including the following: (B) amount of analyte adsorbed at different % of MOF loadings and (C) amount of analyte absorbed per gram of MOF in the PVDF/DUT-52 MMM.

The MOF-based MMMs with 15, 30, 45, and 60% (w/w) of MOF loadings were used to adsorb six PCPs from aqueous solutions: methylparaben (MePB), ethylparaben (EPB), propylparaben (PPB), benzylparaben (BzPB), benzophenone (BP), and benzophenone-3 (BP3) (Figures 4A–C and S12). The amount of analyte adsorbed on the MOF (*q*) is calculated by the difference between the initial analyte concentration (*C*_0_) and the final concentration after 24 h of adsorption (*C*_f_). In addition, the volume of the solution (*V*_s_) and the mass of MOF (*m*) must be taken into account as shown in eq 1.
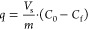
1

The amount of analyte adsorbed increases with the MOF loading into the MMM, as expected (Figures 4B and S12). However, it does not increase proportionally, as occurred in gas adsorption (Figure 4C). This is the reason why *q*_e_ is higher for low MOF-loaded MMMs, although the 60% loaded MMM shows the best uptake. The reason for this is not the competition between analytes but the adsorption and diffusion of analytes through the MOF pores.^27^

### Adsorption Kinetics

**Figure 5:**
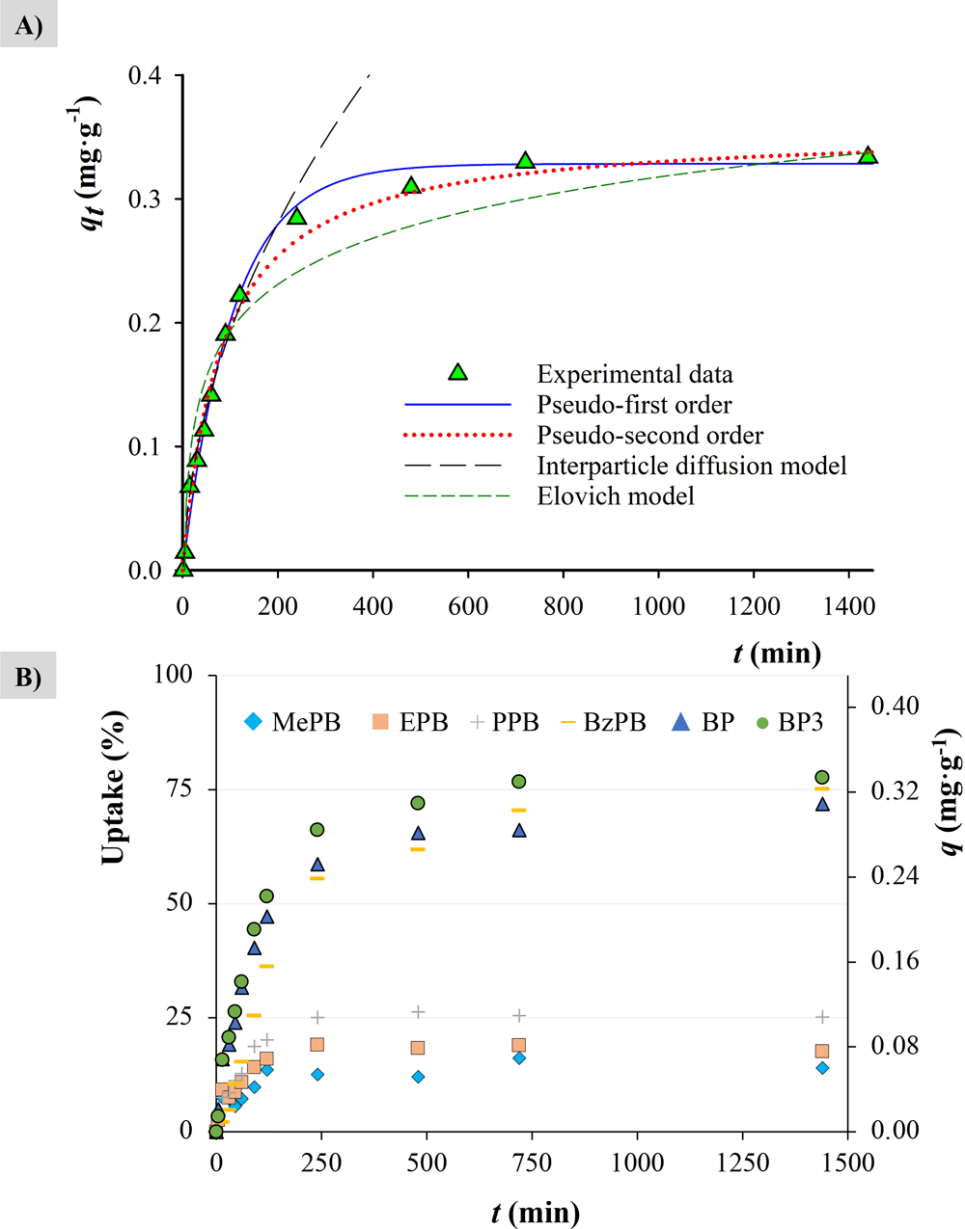
(A) Kinetic fitting models for BP3 using PVDF/DUT-52 MMM (60%, w/w) and (B) uptake (%) and *q* (mg·g^–1^) of the six PCPs for the PVDF/DUT-52 MMM (60%, w/w).

Adsorption processes are driven by kinetically controlled mechanisms that can vary from few seconds to several hours, weeks, or even years. To study this phenomenon, 60% (w/w) MOF-based MMMs were immersed into an aqueous solution containing all the six PCPs at a concentration level of 100 μg·L^–1^. Aliquots of the supernatant solution were taken (in the μL order) and analyzed, so the amount of adsorbed analyte can be determined at different times. The experimental results are fitted to pseudo-first, pseudo-second, Elovich, and interparticle diffusion models (Figure 5A).

There is not a clear kinetic mechanism well defined for all the cases (Figures S13–S19). The MOF DUT-52 shows the highest adsorption capacity for the PCPs, together with the best fittings in terms of correlation coefficient (*R*^2^). Considering the *R*^2^ values (Tables S1–S7), the pseudo-second-order model seems to be the most relevant (Table S8). However, none of the proposed models perfectly match the experimental data, indicating most likely that there is more than one mechanism involved in the adsorption of these analytes. Besides, because there is a mixture of analytes in the solution (mimicking the real situation when using these devices in complex samples), cooperative and competitive effects cannot be ruled out.

The adsorption kinetics of the six PCPs for the PVDF/DUT-52 MMM (as a representative example) are shown in Figure 5B. The values of uptake and *q* graphics for the remaining six MOFs are included in the ESM (Figure S20). From an adsorption capacity point of view, there are clearly two different groups of analytes. Smaller PCPs (MePB, EPB, and PPB) with low uptakes (under 10%) and *q* values (below 0.1 mg·g^–1^) for all the MOFs except for DUT-52 (uptakes are in the range of 15–25%). Larger PCPs, such as BP and BP3, have considerably higher uptakes, reaching values around 55% for BP and 75% for BP3, and *q* values ∼ 0.3 mg·g^–1^ in both cases. This might be related to the chemical structure of BP and BP3, as they have two aromatic groups as substituents, while the smaller PCPs have only one aromatic ring and a linear substituent (Table S9). The aromatic groups can improve the extraction via π–π stacking interactions with the linkers of the MOFs. Besides, the adsorption of less polar compounds such as BP and BP3 is higher than the adsorption of more polar compounds. The attaining of improved extractions due to the increased availability of π–π stacking interactions has been previously reported by other authors for other analytes, such as parabens^27^ and triazines.^28^ Increasing the aromaticity and the number of π–π donor groups of the MOF linker clearly benefits the adsorption of this kind of compounds.^28−30^

The adsorption capacities of the neat MOFs were also measured. Thus, 5 mg of the neat MOF was immersed into an aqueous solution containing all the six PCPs at a concentration level of 100 μg·L^–1^. Aliquots of the supernatant solution were taken (in the μL order) and analyzed, so the amount of adsorbed analyte can be determined after 24 h. The *q* values for neat MOFs ranged from 0.01 to 0.29 mg·g^–1^ (Table S10). As it happened with MOF-based MMMs, PCPs with a larger size have considerably higher uptakes, reaching values around 50% for BP and 80% for BP3. These adsorption values are slightly lower than the values obtained with MOF-based MMMs.

**Figure 6:**
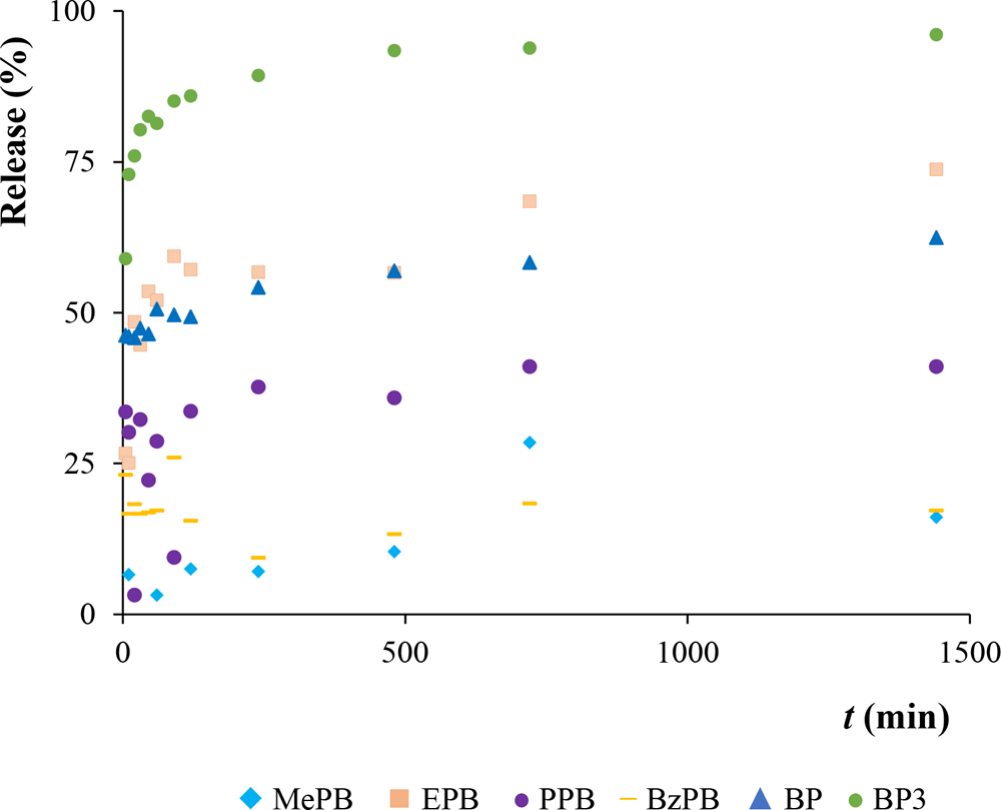
Release (in %) of the six PCPs trapped by the PVDF/DUT-52 MMM (60%, w/w).

For the release studies (in %), the MOF-based MMMs were immersed into aqueous solutions containing all the six PCPs at a concentration level of 100 μg·L^–1^ for 24 h. Then, the MOF-based MMMs containing the trapped analytes were removed from the aqueous standard solution, followed by immersion into 5 mL of MeOH to perform the release of the analytes trapped. Aliquots of such MeOH supernatant solution were taken (in the μL order) and analyzed, and thus, the amount of desorbed analytes can be determined at different times. As observed in Figure 6 and in Figure S21, PCPs with a larger size also show higher release (%) values, reaching values around 62% for BP and 96% for BP3. This is indicative that these larger analytes are more loosely attached to the MOF framework, and those with small size can diffuse better within the MOF pores while establishing stronger interactions with their functional groups. It is also important to consider that the amount of analyte adsorbed is much higher for those larger-size PCPs. Thus, better measurements and fittings can be achieved for them.

**Figure 7:**
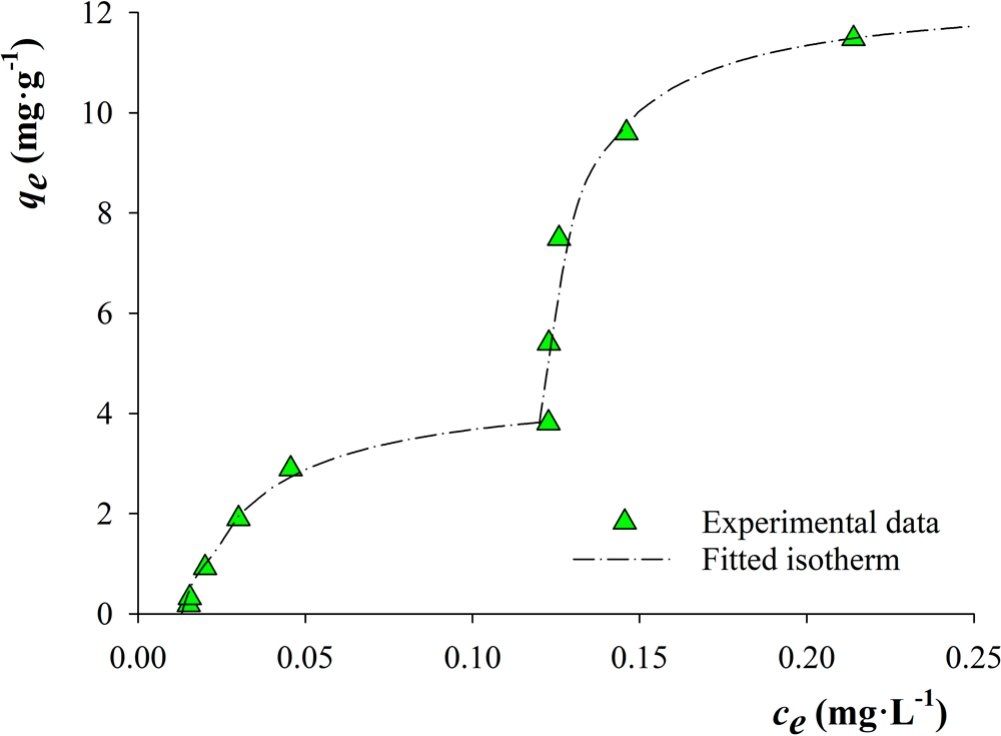
BP3 adsorption isotherms for the PVDF/DUT-52 MMM (60%, w/w).

DUT-52 is a network with *fcu* topology, incorporating octahedral and tetrahedral micropores with 9.3 and 7.5 Å in diameter, respectively, and a limiting pore window of 4.35 Å.^21^ According to the quantum mechanics kinetic diameter for the studied analytes (Table S9), all the analytes could fit inside the pores. Among the PCPs studied, BP3 is the largest one (Figure S22). Thus, BP3 may be retained on the surface of the MOF instead of the inner of the pores, while the remaining PCPs can get trapped inside the pores. The adsorption of BP3 over the surface of the MOF may cause a blocking effect on the pore window of the MOF, avoiding the entrance of the analytes on the pore when a competitive adsorption is taking place.

Regarding the equilibrium time (with high importance for real laboratory studies), it can be concluded that equilibrium time is reached between 240 and 480 min (6–8 h) of adsorption for all the MOFs.

The PVDF/UiO-67 MMM showed the highest *q*_e_ values for the six analytes studied, with values ranging from 0.10 to 0.97 mg·g^–1^. This is related to the pore size of the MOF. Despite this, the UiO-67 structure can collapse due to the removal of water from the pores if it is not carefully done.^4^ In terms of adsorption capacity, PVDF/DUT-52, PVDF/MOF-808, and PVDF/UiO-66 are highly attractive, as the *q*_e_ values are higher than 0.41 mg·g^–1^. Among them, only PVDF/DUT-52 was able to extract all the analytes. With respect to the remaining MOF-based MMMs (PVDF/DUT-67 and PVDF/MOF-801), their adsorption capacity is the lowest, making their use less attractive for microextraction strategies.

In order to determine whether the adsorption of the analytes is related to the presence of MOF or not, neat PVDF membranes were used as sorbents. The amount of analyte adsorbed on the membrane was negligible, confirming that the main parameter responsible for the adsorption is the MOF used as filler in the devices.

### Adsorption Isotherm

As BP3 showed the highest adsorption values with all the MOFs in this study, it was selected as the model compound for the adsorption isotherm study. BP3 has a solubility of 3.7 mg·L^–1^ in water. Thus, the concentrations used for the isotherm adsorption ranged from 0.05 to 3 mg·L^–1^ (Figure 7). For the measurements, the MOF-based MMM devices were immersed in the aqueous solutions containing PCPs under continuous shaking for 24 h. Aliquots at *t* = 0 h and *t* = 24 h were taken to determine the amount of compound adsorbed.

Three different types of isotherms are observed when using the MOF-based MMMs (Figure S23). In the case of PVDF/UiO-66 MMM, it corresponds to a single-step Langmuir isotherm, while PVDF/MOF-801 MMM shows a better fitting with a sigmoidal Freundlich isotherm. For the remaining MOFs-based MMMs, a double-step isotherm was observed. Table 2 shows the isotherm constant values for the studied MOF-based MMMs.

**Table 2:** Parameters of the Adsorption Isotherms for the PVDF/MOF MMMs^g^

Czinkota Isotherm						
	first step			second step		
	*R*^2^^a^	*a*^b^ (mg·g–1)	*k*^c^ (L·mg–1)	*a*^a^ (mg·g–1)	*k*^c^ (L·mg–1)	*b*^d^
UiO-66-COOH	0.995	3.3 (0.3)	34 (8)	29 (12)	2.6 (1.4)	0.07
UiO-67	0.956	4.3 (0.6)	21 (8)	15 (1)	7.3 (0.5)	0.23
DUT-52	0.998	4.4 (0.2)	45 (6)	8.6 (−)	86 (−)	0.12
DUT-67	0.990	5.5 (0.6)	15 (4)	6.4 (0.6)	55 (19)	0.14
MOF-808	1.000	4.3 (−)	11 (−)	7.6 (0.3)	18 (1.5)	0.07

Langmuir Isotherm			
	*a*^a^ (mg·g–1)	*k*^c^ (mg·L–1)	*R*^2^^a^
UiO-66	46 (14)	1.7 (0.7)	0.9924

Freundlich Isotherm			
	*F*^e^ (L·g–1)	*n*^f^	*R*^2^^a^
MOF-801	65 (3)	1.32 (0.05)	0.9953

a Determination coefficient.

b Adsorption capacity.

c Adsorption rate constant.

d Critical concentration.

e Freundlich constant.

f Surface heterogeneity constant.

g –Uncertainty cannot be calculated, and standard deviation of the data is shown in parentheses.

Single-step isotherms are the most widely known and studied. The adsorption of the analyte can take place forming an ordered monolayer at specific sites as described by the Langmuir isotherm (eq 2) or multilayer adsorptions over heterogeneous surfaces as described by the Freundlich isotherm (eq 3).
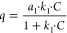
2
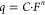
3

However, a more complex procedure takes place during the adsorption of the analytes over the surface for multistep adsorptions. Each step represents different specific types of adsorption mechanisms, which are differentiated by a critical concentration.^31^

These kinds of multistep isotherms were first described by Czinkota et al.^32^ In this approximation, the isotherm is expressed as the sum of the Langmuir-type isotherms of the steps. For two-step adsorptions, the mathematical expression is (eq 4)
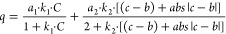
4where *a* are the adsorption capacities (mg·g^–1^), *k* are the adsorption equilibrium constants (L·mg^–1^), *c* is the equilibrium analyte concentration in solution (mg·L^–1^), and *b* is the critical concentration (mg·L^–1^).

Two-step isotherms are related to structures with more than one type of pore. This behavior of multistep adsorption has been previously described for some MOFs, such as UiO-67 and DUT-67, during water vapor adsorption isotherm measurements. Both MOFs show two-step adsorption due to the successive filling of pores with different sizes.^33,34^ Among the seven MOF-based MMMs studied, DUT-52-based MMM shows the higher value of *k* for the two steps observed, which is related to a high affinity between the sorbent and the adsorbate. DUT-52 has two defined kinds of micropores: octahedral (9.3 Å) and tetrahedral (7 Å) pores. From the results, it is not possible to determine the filling order of the pores. Nevertheless, it can be assumed that it is taking place as a sequential filling.

Scarce studies have been published with respect to the removal of BP3, and those reported in the literature employs an ethanolic solution of BP3 at high-concentration levels or do not report the extraction capacity of the material. As it can be observed in Table S11, the adsorption capacity of the PVDF/DUT-52 MMM is higher than the extraction capacity of materials such as graphene oxide, polysulfone, and the composite graphene oxide–polysulfone.

### Analytical Application

Table S12 shows several quality analytical parameters of the HPLC–UV/vis method. The calibration curves were obtained by direct injection of 20 μL of all calibration standard solutions. Calibration curves were linear, with *R*^2^ higher than 0.999. Limits of detection (LODs) and quantification (LOQs) were calculated as 3 and 10 times the signal-to-noise ratio, respectively. Thus, chromatographic LODs ranged from 0.5 to 1.0 μg·L^–1^ for MPB and BP3, respectively, and chromatographic LOQs ranged from 1.5 to 2.5 μg·L^–1^ for MPB and BP3, respectively. The chromatographic precision was evaluated in terms of intra-day and inter-day relative standard deviation (RSD, in %) at a concentration level of 40 μg·L^–1^ in quadruplicate. For both, intra-day and inter-day, RSD values (in %) were lower than 3.7 and 4.8%, respectively.

**Figure 8:**
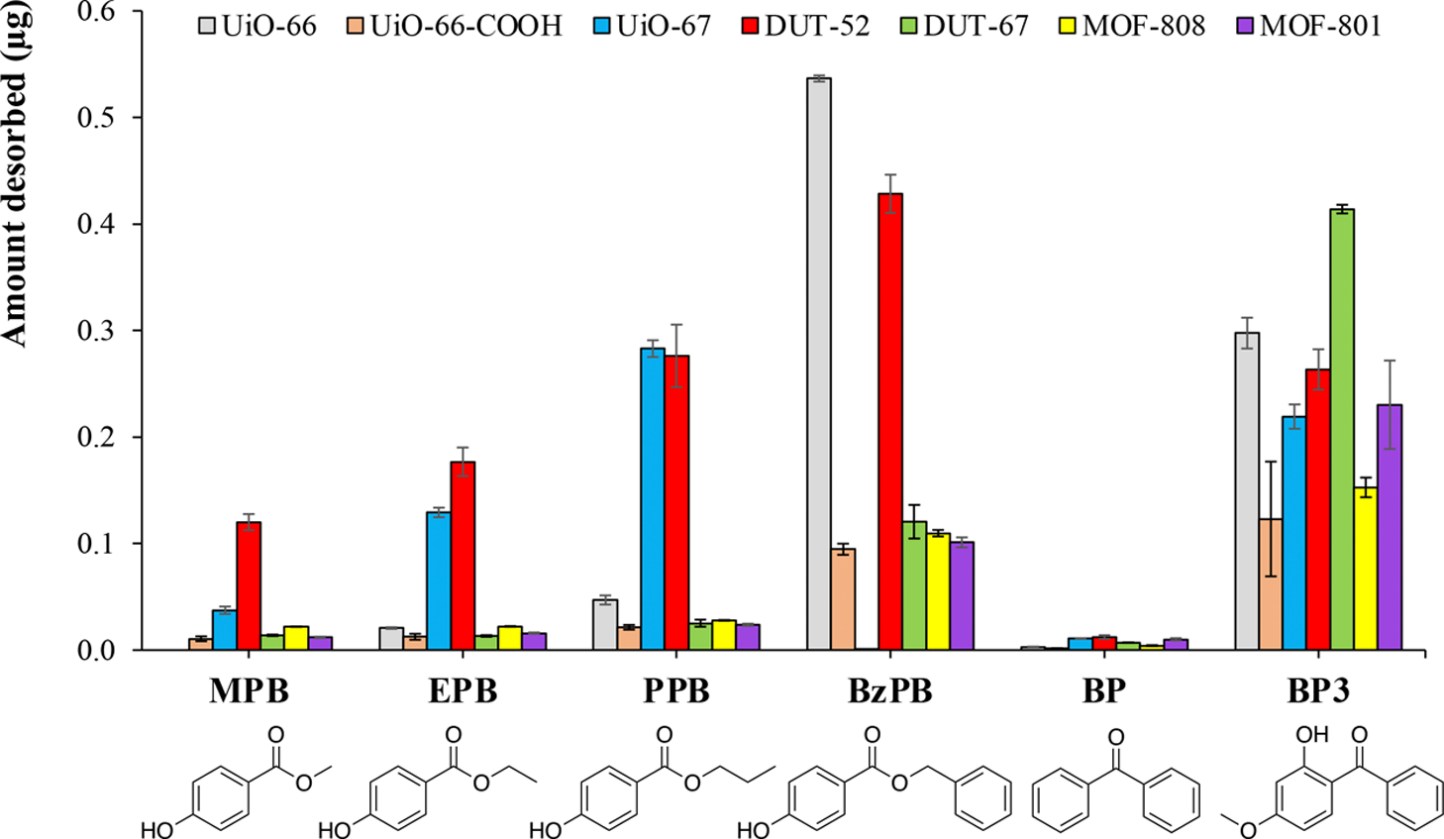
Extraction capacity, as mass of analytes finally desorbed from the seven MOF-based MMMs, extracting aqueous standards at the 100 μg·L^–1^ level.

The extraction efficiency of the MOF-based MMMs was evaluated as a proof of concept (Figure 8). As it can be observed, MOF-based MMMs showed good extraction performance and good precision, demonstrating their applicability for the determination of PCPs in aqueous samples. The precision was measured as the RSD (RSD %) of the amount of analyte desorbed while performing the extractions in triplicate to an aqueous standard solution at a concentration level of 100 μg·L^–1^. The RSD (%) values ranged from 2.3 to 24% (Table S13). The analytical acceptance value for trace levels in water must be below 25%. Besides, enrichment factors (*E*_F_) were calculated to determine the preconcentration capacity achieved with the seven membranes. *E*_F_ was calculated as the ratio of the concentration of the analyte in the final extract (*C*_F_) and the concentration of the analyte in the initial standard solution (*C*_0_) (eq 5).
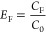
5

As it can be observed in Table S14, PVDF/DUT-52 MMM showed the highest *E*_F_ for most of the analytes among all MOF-based MMMs studied. This MOF has not been widely explored in real applications. Thus, the employment of DUT-52 in MMMs as adsorbent in microextraction techniques for the determination of pollutants of emerging concern at trace levels, in complex samples, is a highly interesting topic to be further studied.

Besides, the extraction efficiency (*E*_R_, in %) of the MOF-based MMMs were calculated comparing the maximum expected *E*_F_ and the obtained *E*_F_ (eq 6).
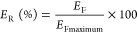


The maximum (theoretical) expected *E*_F_ was calculated considering the initial volume of the sample and the final volume of the extract, assuming a theoretical 100% recovery. As it can be observed in Table S15, PVDF/DUT-52 MMM showed the highest *E*_R_ (%) for most of the analytes. It is important to highlight that thin-film microextraction is a nonexhaustive extraction technique, and clearly, low *E*_R_ (%) values are expected as this is not the main goal of the technique but to obtain high sensitivity. In other words, this type of technique is intended for lower detection limits and adequate reproducibility instead of quantitative extraction.^35^

Based on the LODs of the HPLC–UV/vis method and considering the abovementioned *E*_F_ values for the seven MOF-based MMMs, it was possible to estimate the LODs of the entire methodology MOF-based MMMs with HPLC–UV/vis, as the ratio of the chromatographic LODs and the *E*_F_.

As it can be observed in Table S16, the obtained LODs for the entire methodology ranged between 0.03 and 8 μg·L^–1^. These low values ensure the successful application of the MOF-based MMMs for the determination of PCPs in cosmetic products, as the concentrations of parabens and ultraviolet filters incorporated in cosmetics are in the range of ^—^milligram per liter. Besides, the low values of LODs for MOF-based MMMs open up the possibility of applying these materials to other samples with lower concentrations of analytes such as environmental waters.

To test the DUT-52/PVDF MMM performance as extractant material with real samples, three commercial cosmetic samples were analyzed for the determination of the six PCPs. One of the samples was declared to be free of parabens and BPs by the manufacturer, while the other two samples contained parabens (as claimed in their labels). For the paraben-free cosmetic (sample 1), none of the six PCPs were detected. On the other hand, for the other two cosmetic samples, it was possible to detect and quantify some of the studied PCPs. Thus, sample 2 contained MePB and EPB at concentrations of 24 and 11 mg·L^–1^, respectively. Sample 3 contained MePB, EPB, and PPB at concentrations of 10, 3.6, and 1.9 mg·L^–1^, respectively. Besides, the label of sample 3 also indicated that it contained butylparaben (BuPB). Figure S24 shows the chromatograms corresponding to the final extract of the samples obtained after the MOF-based MMM extractions. In sample 3, it was possible to observe an unknown fourth peak, which may be attributed to BuPB. Thus, the potential of DUT-52/PVDF MMM as extractant material for the developing of a microextraction device using thin-film membranes to monitor PCPs in complex samples has been demonstrated.

## Conclusions

In this study, seven different Zr-MOF-based MMMs were successfully prepared and characterized for their use as a potential miniaturized device in environmental monitoring. Through the physical characterization studies, the proper preparation of the MMMs was demonstrated, confirming the maintenance of the polymer and MOFs integrity by different techniques. The combination of both materials resulted in a high synergic effect, taking advantage of the flexibility offered by PVDF and the higher adsorption and selectivity offered by MOFs. The resulting properties of the MMMs resembled closely those of the respective MOF loaded on the membrane. The increase of amount of MOF loaded decreased the mechanical flexibility but increased the adsorption capacity.

With respect to the adsorption kinetics, different kinetic models fitted better the adsorption procedure depending on the MOF and the PCP studied, with the pseudo-second-order kinetic being one of the most common. Besides, the intraparticle diffusion model showed a good fitting for the initial adsorption. However, it should be highlighted that not a pure and single kinetic model was observed for PCP adsorption, and most likely the adsorption procedure takes places by a combination of processes.

Among the six PCPs studied, the Zr-MOF-based MMMs showed particular adsorption of aromatic-containing substituents and therefore compounds such as BzPB, BP, and BP3 that may be promoted by π–π stacking interactions with the MOF linkers, thus demonstrating certain selectivity. The adsorption isotherms obtained with BP3 (the one with highest affinity toward the MMMs) presented a multistep isotherm due to the progressive filling of pores with different sizes.

Finally, the seven Zr-MOF-based MMMs were tested as possible analytical microextraction devices for the extraction of PCPs in aqueous-based cosmetic samples. All the MOFs showed good extraction performance and reproducibility. Among the studied Zr-MOF-based MMMs, DUT-52/PVDF showed the highest extraction efficiency of PCP, thus becoming a potential device for analytical applications.

## Experimental Section

### Materials and Methods

Reagents for the preparation of the MOF-based MMMs included the following: zirconium(IV) oxychloride octahydrate (ZrOCl_2_·8H_2_O, 98%), supplied by Honeywell (Germany). Zirconium(IV) chloride (ZrCl_4_, 98%) and fumaric acid (C_4_H_4_O_4_, 99%) were purchased from Merck (Germany). 2,5-Thiophene dicarboxylic acid (H_2_TDC, 100%), benzene-1,3,5-tricarboxylic acid (H_3_BTC, 95%), 2,6-naphthalene dicarboxylic acid (H_2_(2,6-NDC), 98%), 1,2,4-benzenetricarboxylic anhydride (1,2,4-BTC, 97%), and sodium acetate (99%) were all from Aldrich (USA). Biphenyl-4,4′-dicarboxylate (BPDC, 97%) was supplied by Intatrade Chemicals (Germany). Benzene-1,4-dicarboxylic acid (H_2_BDC, 99%) was acquired from Acros Organics (USA). Formic acid (98%) and acetic acid (100%) were purchased from Carl Roth (Germany). DMF (99.5%) was supplied by Fisher Chemical (UK). Chlorohydric acid (37%) was acquired from VWR Chemicals (France). PVDF was purchased from Alfa Aesar (Germany).

MOFs were synthesized using stainless steel autoclaves and Schott bottles. MMMs were prepared by bar coating using a UA3000 Universal-film applicator with an adjustable gap height, and an automatic precision film applicator CX1, both from mtv messtechnik oHG (Germany), and glass microscope slides (7.5 × 2.5 cm) acquired from Thermo Scientific (Germany).

Six PCPs including four preservatives and two UV filters were used for the adsorption studies. MePB (99%), EPB (99%), and PPB (98%) were acquired from Dr. Ehrenstorfer (Germany). BzPB (99%), BP (99%), and BP3 (98%) were supplied by Sigma-Aldrich (Germany). Individual standard solutions at concentrations ranging from 1060 to 4256 mg·L^–1^ were prepared in acetonitrile (ACN) (99.9%) of LC–MS grade, purchased from VWR Chemicals (Spain). Intermediate standard mix solutions containing the six analytes at concentration levels of 50, 10, and 1 mg·L^–1^ were prepared in ACN and stored in the fridge protected from light at 4 °C. Working aqueous standard solution were prepared daily by dilution of the intermediate solution using an acetic/acetate buffer solution (pH = 5) prepared using ultrapure Milli-Q water obtained by the water purification system A10 Millipore (Watford, UK), glacial acid acetic acquired from Honeywell, and sodium acetate anhydrous (99%) from Sigma-Aldrich.

### Characterization and Instruments

Phase identification studies were performed with an STOE STADI P diffractometer using Cu Kα_1_ radiation (λ = 1.5406 Å) and a 2D detector (Mythen, Dectris). Data collection was carried out in transmission geometry using a rotating flatbed sample holder and over the angular range from 5 to 55°. Data analysis was carried out using WINXPOW.

The N_2_ adsorption isotherms studies were performed using a Quadrasorb SI at 77 K, with liquid nitrogen to reach the measurement temperatures. Specific surface areas were calculated applying the Brunauer–Emmett–Teller (BET) method in the range from 0.05 to 0.3. The pore size distribution of the materials was calculated applying the density functional theory (DFT) method.

A Bruker Vertex 70 FT-IR spectroscope using a 2 cm^–1^ resolution from 600 to 4000 cm^–1^ was used for the identification of functional groups of the synthetized MOFs and MOF-based MMMs.

TGA was performed on a NETZSCHSta 409C/CD from NETZSCH-Gerätebau GmbH (Germany) at a heating rate of 10 K·min^–1^ in a temperate range from 25 to 700 °C in the air atmosphere.

MOFs and MOF-based MMM morphology was characterized by SEM using a Hitachi SU8020 scanning electron microscope (Japan). Samples were sputtered with Au before measuring to ensure adequate surface conductivity.

Chromatographic analysis was performed using the UHPLC 1260 Infinity Series from Agilent Technologies (USA) controlled by Agilent OpenLAB control Panel ChemStation. The equipment is supplied with a quaternary pump, an InfinityLab Poroshell 120 EC-C18 (4.6 × 50 mm, 2.7 μm particle size) column, and a Rheodyne 7725i injection valve with a loop of 5 μL. The detector used was a vis/UV ProStar 325 LC detector series supplied from Varian (USA). The separation of the analytes is achieved under the gradient mode using a binary mobile phase composed of ACN and water [0.1% (v/v) of acetic acid] at a flow rate of 0.5 mL·min^–1^ and at a constant temperature of 298 K. The mobile phase gradient used was as follows: initially 50% (v/v) of ACN, kept isocratic during 1 min, followed by a linear elution gradient increase to 90% (v/v) of ACN in 7 min, and then kept isocratic for 1 additional minute. The wavelength of the detector was fixed at 254 nm.

An IKA shaker KS3000i control model from Janke&Kunkel was used for the adsorption and desorption studies at 298 K and 225 min^–1^. Corning 50 mL polypropylene conical bottom centrifuge tubes (USA) with dimensions of 11.5 × 2.9 cm were used for the adsorption studies.

SigmaPlot software (Systat Software, USA) was used to fit the experimental data to the different models and equations studied on this work. MOF structures were visualized using the crystal and molecular structure visualization software Diamond 4.6.4. Crystallographic information framework (CIF) files of the MOF structures studied on this work were downloaded from the Cambridge Crystallographic Data Center (CCDC).

### Synthesis of Zr-Based MOFs

The seven zirconium MOFs (UiO-66, UiO-66-COOH, UiO-67, DUT.52, DUT-67, MOF-801, and MOF-808) were synthesized following the experimental procedure described with slight modifications.^18−22^ Further details of their synthesis are included in the Supporting Information.

### Preparation of MOF-Based MMMs

The preparation of polymeric MOF-based MMMs with different loads of MOF [15, 30, 45, and 60% (w/w)] is based on an already described procedure.^12^ Briefly, a MOF suspension containing the required amount of MOF and acetone in the specific ratio 30:1 (mg/mL) is prepared by sonication during 25 min, and subsequently stirred for 5 min. Then, 1.0 g of a polymeric solution of PVDF in DMF (7.5%, w/w) is mixed under stirring with the MOF colloidal suspension. Afterward, the acetone is evaporated under an air stream with continued stirring. The resulting MOF-PVDF “ink” is spread at a speed of 100 mm·s^–1^ over a glass slide using a film applicator with a gap height of 250 μm. Finally, the remaining DMF is removed heating the film at 70 °C for 1 h in an oven. Finally, the MOF-based MMM is peeled from the glass slide by immersion in methanol.

### Adsorption Studies

In all the adsorption studies, the MOF-based MMMs are cut into rectangular pieces with dimensions of 1.50 × 1.70 cm (2.55 cm^2^) and kept into a plastic holder to ensure its flat format (and it is maintained like that despite strong stirring rates) when further immersed in solutions. These pieces of MOF-based MMMs are immersed in 20 mL of aqueous acetate/acetic buffer (pH = 5) working standard solution containing the six PCPs at a concentration level of 100 μg·L^–1^, at a controlled temperature of 25 °C, and under continuous mechanical shake at 250 min^–1^.

For the studies evaluating the effect of the amount of MOF loaded in the MMM, MOF-based MMMs at 15, 30, 45, and 60% MOF loadings were immersed in the working solution during 24 h (also using plastic holders). Measurements of the upper natant at *t* = 0 min and *t* = 24 h were performed to calculate the amount of analytes extracted per gram of MOF.

Kinetic studies were performed using the 60% (w/w) MOF-based MMMs by immersing each membrane (with the plastic holder) in the working solution, and 12 aliquots (100 μL each one) of the upper natant solution were taken at 0, 5, 15, 40, 45, 90, 120, 240, 480, 720, and 1440 min to measure the remaining amount of PCP in the solution. The results were fitted through nonlinear regression to pseudo-first-order (eq 6), pseudo-second-order (eq 7), Elovich (eq 8), and intraparticle diffusion (eq 9) models.^36,37^
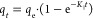
6
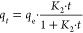
7
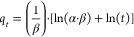
8
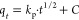
9where *q*_*t*_ (mg g^–1^) is the amount adsorbed at a given time *t*, *q*_e_ (mg g^–1^) is the amount adsorbed at equilibrium, *K*_1_ (min^–1^) is the pseudo-first-order adsorption kinetics parameter, *K*_2_ (min^–1^) is the pseudo-second-order parameter, α (mg g^–1^ min^–1^) is the initial adsorption rate, β (g mg^–1^) is the Elovich constant, *k*_p_ (mg g^–1^ min^1/2^) is the intraparticle diffusion rate, and the constant *C* (mg g^–1^) provides information about the thickness of the boundary layer.

Release studies were performed using the 60% (w/w) MOF-based MMMs by immersing each membrane (with the plastic holder) in the working solution for 24 h. Right after that, the MOF-based MMMs were removed from the aqueous standard solution and immersed in 5 mL of MeOH. 12 aliquots (50 μL each one) of the supernatant solution were taken at 0, 5, 15, 40, 45, 90, 120, 240, 480, 720, and 1440 min to measure the amount of PCP released in MeOH.

Adsorption capacity studies were performed immersing the MOF-based MMMs in 20 mL of aqueous acetate/acetic buffer (pH = 5) solutions of BP3 at concentrations of 50, 100, 250, 500, 750, 1000, 1500, 2000, 2500, and 3000 μg·L^–1^ during 24 h. Then, the upper natant of the solutions were measured, and the treated data were fitted to the proper mathematic isotherm model.

### Analytical Application Studies

MOF-based MMMs (with the plastic holder) were immersed into 20 mL of acetate/acetic aqueous buffer solution (pH = 5) containing the six PCPs at a concentration level of 100 μg·L^–1^ for 90 min under stirring at 500 min^–1^ and at room temperature. Then, the trapped analytes by the MOF-based MMM are desorbed in 5 mL of MeOH for 30 min. Finally, the final desorption extract is collected in a round-bottom flask, evaporated till dryness, and reconstituted in 150 μL of UHPLC mobile phase to perform the chromatographic determination. The experiments were performed in triplicate to include the error associated to the extraction procedure. For the analysis of real cosmetic samples, MOF MMMs were immersed in a 2 mL ethanolic extract of the cosmetic diluted to 20 mL using ultrapure water and the procedure above described for standards was also performed. In all cases, absences of interfering compounds coming from the plastic holders were ensured.

## Supporting Information Available

Experimental details including synthesis of MOFs and preparation of MOF-based MMMs; MOFs and MOF-based MMMs characterization including PXRD, SEM, N_2_ adsorption, TGA, FT-IR, adsorption experiments, and kinetic fitting models; isotherm adsorption fitting parameters; parameters for the analytical application; and analyte properties (PDF)

## Abbreviations

1,2,4-BTC: 1,2,4-benzenetricarboxilic anhydride
ACN: acetonitrile
BET: Brunauer–Emmett–Teller
BP3: benzophenone-3
BP: benzophenone
BPDC: biphenyl-4,4′-dicarboxylate
BzPB: benzylparaben
CCDC: Cambridge Crystallographic Data Center
CIF: crystallographic information framework
DFT: density functional theory
DMF: *N*,*N*′-dimethylformamide
EF: enrichment factor
EPB: ethylparaben
FT-IR: Fourier transform infrared
H_2_(2,6-NDC): 2,6-naphthalenedicarboxylic acid
H_2_BDC: benzene-1,4-dicarboxylic acid
H_2_TDC: 2,5-thiophene dicarboxilic acid
H_3_BTC: benzene-1,3,5-tricarboxilic acid
LBL: layer-by-layer
MeOH: methanol
MePB: methylparaben
MMMs: mixed matrix membranes
MOFs: metal–organic frameworks
PCPs: personal care products
PDMS: polydimethylsiloxane
PPB: propylparaben
PVDF: polyvinylidene fluoride
RSD: relative standard deviation
SBUs: secondary building units
SEM: scanning electron microscopy
TGA: thermogravimetric analysis
