# Supplementary material for: Zirconium-Based Metal–Organic Framework Mixed-Matrix Membranes as Analytical Devices for the Trace Analysis of Complex Cosmetic Samples in the Assessment of Their Personal Care Product Content
Source: ACS Appl Mater Interfaces. 2022 Jan 10;14(3):4510–21. doi: 10.1021/acsami.1c21284 (PMC8796172; doi:10.1021/acsami.1c21284)
Supplement: Supplementary file 1 — am1c21284_si_001.pdf [file am1c21284_si_001.pdf]

Supporting Information

for the manuscript

Zirconium-based metal-organic frameworks mixed-matrix membranes as analytical devices for the trace analysis of complex cosmetic samples in the assessment of their personal care products content

Adrián Gutiérrez-Serpa,[†,‡] Tanay Kundu,[ð] Jorge Pasán,[**&] Ana I. Jiménez-Abizanda,[†] Stefan Kaskel,[§] Irena Senkovska,[§] Verónica Pino [*†,‡]

[*] veropino@ull.edu.es
[**] jpasang@ull.edu.es

[†]Laboratorio de Materiales para Análisis Químicos (MAT4ALL), Departamento de Química, Unidad Departamental de Química Analítica, Universidad de La Laguna (ULL), La Laguna, Tenerife, 38206, Spain.
[‡]Unidad de Investigación de Bioanalítica y Medioambiente, Instituto Universitario de Enfermedades Tropicales y Salud Pública de Canarias, Universidad de La Laguna (ULL), 38206 Tenerife, Spain.
[ð]Department of Chemistry, SRM Institute of Science and Technology, Kattankulathur 603203, Tamil Nadu, India.
[§]Technische Universität Dresden (TUD), Bergstrasse 66, 01069, Dresden, Germany.
[&]Laboratorio de Materiales para Análisis Químicos (MAT4ALL), Departamento de Química, Unidad Departamental de Química Inorgánica, Universidad de La Laguna (ULL), La Laguna, Tenerife, 38206, Spain.

Table of contents

Synthesis of MOFs	S3
Preparation of MOF-based MMMs	S5
Figure SI.1. SEM of neat MOFs	S7
Figure SI.2. Influence of ink amount	S8
Figure SI.3. Thickness of MMM	S9
Figure SI.4. MOF loading in MMM	S10
Figure SI.5. XRD patterns of MOFs	S11
Figure SI.6. XRD patterns of MMMs	S12
Figure SI.7. FT-IR	S13
Figure SI.8. TGA	S14
Figure SI.9.-S.10. N_2 adsorption and desorption isotherms of MOFs and MMMs	S15
Figure SI.11. SEM of MMMs	S17
Figure SI.12. Influence of MOF loading	S18
Figure SI.13. Kinetic of UiO-66 MMM	S19
Figure SI.14. Kinetic of UiO-66-COOH MMM	S21
Figure SI.15. Kinetic of UiO-67 MMM	S22
Figure SI.16. Kinetic of DUT-52 MMM	S24
Figure SI.17. Kinetic of DUT-67 MMM	S26
Figure SI.18. Kinetic of MOF-801 MMM	S27
Figure SI.19. Kinetic of MOF-808 MMM	S28
Figure SI.20. Uptake of MMM	S29
Figure SI.21. Release of MMM	S30
Figure SI.22. MOFs and analytes structures	S31
Figure SI.23. Adsorption isotherms of BP3	S32
Figure SI.24. Chromatograms of samples	S34
Table SI.1. Fitting parameters for the kinetic models for UiO-66 MMM	S35
Table SI.2. Fitting parameters for the kinetic models for UiO-66-COOH MMM	S36
Table SI.3. Fitting parameters for the kinetic models for UiO-67 MMM	S37
Table SI.4. Fitting parameters for the kinetic models for DUT-52 MMM	S38
Table SI.5. Fitting parameters for the kinetic models for DUT-67 MMM	S39
Table SI.6. Fitting parameters for the kinetic models for MOF-801 MMM	S40
Table SI.7. Fitting parameters for the kinetic models for MOF-808 MMM	S41
Table SI.8. Kinetic fitting resume	S42
Table SI.9. Analyte's properties	S43
Table SI.10. Uptake of neat MOFs	S44
Table SI.11. Other studies	S45
Table SI.12. Several quality analytical parameters	S46
Table SI.13. Precision study of MMMs	S47
Table SI.14. Enrichment factors of MMMs	S48
Table SI.15. Extraction efficiency of MMMs	S49
Table SI.16. Limits of detection of MMMs	S50
References	S51

Synthesis of the MOFs

Synthesis of [$Zr_6O_4(OH)_4(BDC)_6$] (UiO-66)
UiO-66 is synthesized by adjusting the procedure described by Katz *et al.* [18]. 22 mmol of $ZrCl_4$ are dissolved using 200 mL of DMF and 40 mL of HCl (37%). 30 mmol of H_2BDC are dissolved in 400 mL of DMF. Both solutions were heated at 80 ºC to fully dissolve the linker and metallic cluster. Once the solutions are colorless, they are mixed in a Schott bottle and heated at 110 ºC during 24 h in an oven. The white crystalline powder is collected by filtration and washed with fresh DMF and ethanol by Soxhlet. Finally, the MOF is dried and activated at 120 ºC overnight.

Synthesis of [$Zr_6O_4(OH)_4$(1,2,4-BTC)$_6$] (UiO-66-COOH)
UiO-66-COOH is synthesized following the procedure described by Ragon *et al.* with slight modifications [19]. Briefly, 5 mmol of $ZrCl_4$ and 10 mmol of 1,2,4-BTC are dissolved in 25 mL of H_2O and set under reflux at 100 ºC for 24 h. Afterwards, it is cooled down to room temperature, washed with deionized H_2O and filtrated. The white powder is re-suspended in 80 mL of H_2O and refluxed for 16 h. Finally, the MOF is filtered and dried for 48 h at 120 ºC.

Synthesis of [$Zr_6O_4(OH)_4(BPDC)_6$] (UiO-67)
UiO-67 is synthesized following the procedure described by Katz *et al.* introducing some modifications [18]. Briefly, 14 mmol of $ZrCl_4$ are dissolved in 260 mL DMF and 26 mL of HCl (37%). Then, 20 mmol of BPDC are dissolved in 525 mL of DMF. Both solutions are mixed and placed in a Schott bottle. The synthesis took place at 120 ºC for 48 h in an oven. Crystals are collected by centrifugation (2575 × g, 30 min) and washed with fresh DMF and ethanol three times respectively. Finally, the crystalline powder is dried at 80 ºC overnight. Before its use, MOF is activated at 150 ºC.

Synthesis of [$Zr_6O_4(OH)_4(NDC)_6(H_2O)_6$] (DUT-52)
DUT-52 synthesis is based in the method reported by Volodymyr *et al.* [21], modifying some parameters to obtain larger amounts of MOF. 27.5 mmol of $ZrCl_4$ is dissolved in 209 mL of DMF and 41 mL of HCl (37%). Then 31 mmol of H_2(2,6-NDC) are dissolved in 417 mL of DMF. Both solutions are mixed in a Schott bottle and heated in an oven at 120 ºC during 24 h. The white crystalline powder formed is filtered and washed twice with fresh DMF and three times with ethanol. Then, it is dried at room temperature. Before using the MOF, it is activated at 120 ºC overnight under vacuum.

Synthesis of [$Zr_6O_4(OH)_8(TDC)_4(H_2O)_4$] (DUT-67)
DUT-67 synthesis is based on the procedure proposed by Helge *et al.* with few modifications [22]. 10 mmol of $ZrOCl_2 \cdot 8H_2O$ and 6.7 mmol of H_2TDC are dissolved using 50 mL of acetic acid:H_2O (50:50, v/v). The resulting solution is refluxed at 110 ºC for 24 h. After cooling down to room temperature crystals are collected by centrifugation (2575 × g, 45 min) and redispersed in 15 mL of a 0.1 M sodium acetate solution, three times. Finally, the white powder is washed with 15 mL of H_2O twice. DUT-67 is dried at 95 ºC overnight.

Synthesis of [$Zr_6O_4(OH)_4(C_4H_2O_4)_6$] (MOF-801)
MOF-801 is synthesized based on the method reported by Yun *et al.* with some modifications [20]. Briefly, 11 mmol of $ZrCl_4$ are dissolved in 65 mL of H_2O and 27 mL of HCl (37%). Then, 30 mmol of fumaric acid are dissolved in 130 mL of H_2O. Both

solutions are mixed in a Schott bottle and heated at 120 ºC for 24 h. The resulting crystalline powder is washed with H_2O and ethanol several times. Finally, the MOF is dried at room temperature.

Synthesis of $[Zr_6O_4(OH)_{10}(BTC)_2(H_2O)_6]$ (MOF-808)
MOF-808 is synthesized by reflux following the procedure described by Helge *et al.* [22]. 20 mmol of $ZrOCl_2{\cdot}8H_2O$ and 6.6 mmol of H_3BTC are dissolved using 200 mL of a mixture of H_2O:acetic acid (50:50, v/v). Once the chemicals are fully dissolved, the solution is heated at 120 ºC for 24 h. Afterwards, the withe crystalline powder is collected by filtration and washed several times using H_2O and ethanol. Finally, the MOF is activated in an oven at 150 ºC for 24 h.

MOF-based MMMs preparation

For the preparation of the MOF-MMMs, different parameters were studied to ensure the obtaining highly homogeneous membranes. For this purpose, neat PVDF, and PVDF/UiO-66 at the maximum load (60% (w/w)), were used as model materials. The 60% loading was selected as this loading amount is the less favorable for their preparation. Studied parameters were: dispersion method used for obtaining a homogeneous pre-ink, solvent evaporation method for ink preparation, spreading speed, amount of ink used, and thickness of the MMM.

A key factor to obtain highly homogeneous and reproducible MOF-inks is to ensure/achieve a proper dispersion of the MOFs in the polymeric solution of PVDF. Several methods have been reported for this step, being ultrasonic-assisted dispersion the preferred one. However, this method can be not valid for large crystal size, as they can rapidly decant instead of staying dispersed once the sonication is stopped. The seven MOFs studied have different crystal size. Figure SI.1 shows SEM images of the seven Zr based MOFs crystals. For all the MOFs, good particle size distribution can be considered as the crystal size range from an 18% to a 27%. Attending to the SEM images, UiO-66-COOH and UiO-66 are those with a smaller crystal size with an average size of ≈ 176 nm and ≈ 232 nm respectively. The dispersion of these MOFs is easier and more stable than for DUT-52 (≈ 339 nm), DUT-67 (≈ 386 nm), and UiO-67 (≈ 414 nm), which tends to decant faster. In the case of MOF-801 (≈ 300 µm) and MOF-808 (≈ 960 nm), the large size of the crystals makes difficult their proper dispersion in a solvent just by sonication and an extra dispersion method is needed. In addition, smaller particles provide more polymer/particle interfacial area, further improving the membrane performance [S1]. In order to ensure the proper dispersion of the MOF a combined method of sonication followed by stirring is carried out.

Once the MOF suspension in acetone is prepared, it is mixed with the polymeric solution under continuous stirring to form the pre-ink. To ensure the obtaining of the working MOF-ink, it is necessary to remove the acetone while maintaining the MOF suspended in the polymer. Solvent removal was performed using a rotary evaporator and by air stream. The easier operationality avoiding significative loses of ink, while being a faster method, is the application of an air flow under continuous stirring. The time needed to remove the acetone while obtaining the MOF-ink under air stream is ≈ 2 h. The resulting ink is dense and viscous.

Different spreading speeds between 20 and 200 $mm \cdot s^{-1}$ were also tested. Slow spreading speeds affect the uniformity of the MMM as larger crystals can decant forming a crystal containing gradient in the membrane. Fast speeds produce non-uniform MMMs, as the spread of the ink over the glass surface is incomplete. For these reasons, medium spread speed of 100 $mm \cdot s^{-1}$ was selected as the most adequate to obtain uniform MMMs.

The amount of ink necessary to completely cover the glass slide while obtaining large MMM sheets (7.5 × 2.5 cm) was studied using volumes of ink between 0.2 and 0.8 mL. Figure SI.2 shows images related to the influence of the amount of ink used in the resulting membranes. For neat PVDF membranes, 0.4 mL of polymeric ink was the minimum amount to ensure the obtaining of a full sheet of membrane (Figure SI.2.A). However, for MOF-MMMs, the amount of ink required to ensure the obtaining of uniform sheets is 0.8 mL (Figure SI.2.B). This may be related to the lower amount of polymer contained in these membranes, as it is just a 40%. Thus, it is needed to use at least a double amount of ink to get a full sheet of a uniform MOF-MMM sheet.

Different gap size of the spreading bar ranging from 75 µm to 250 µm were tested. Figure SI.3 shows images of the influence of the gap size of the spreading bar. For neat PVDF

membranes (Figure SI.3. A), gaps of 100 µm and 150 µm are enough to prepare the films. On the other hand, for PVDF/UiO-66 MMM, thicker membranes are needed. Thus, the gap size of the spreading bar has to be the doble (250 µm) than for neat PVDF (Figure SI.3.B). This is also related with the % of polymer. For highly MOF loaded membranes, the decrease in the polymer content difficult the formation of the films, as the amount of filler material is considerably low, in turn getting a lower level of agglomeration and embedding of the crystals. Thus, 250 µm is selected as MMMs optimum gap size of the spreading bar. Using this gap size of the spreading bar, the obtained MOF-based MMMs (once the solvent has been removed) have a thickness of 60 µm (Figure 3 E and F).

In order to proof the proper performance of the membranes obtained when using the selected conditions for the preparation of the MMMs, several membranes were prepared to check their uniformity, the easiness of their preparation, and the effectivity of the established procedure (Figure SI.3.C). MMMs show high uniformity, making their preparation highly interesting due to its easiness and effectiveness. A 95 % of the prepared membranes did not show any physical damage or defect naked eye. Therefore, the conditions selected were applied for the preparation of MMMs at 15 %, 30 %, 45 % and 60 % of the seven MOFs. Figure SI.4 shows the as prepared MMMs for all the MOFs. The increase on the % of MOF loaded is directly related with the opacity and flexibility of the MMMs. At higher % of MOF the opacity is higher, getting white like colored, and a loose of their flexibility is also noticed. MOF-MMMs with higher amounts of loaded MOF have been reported in literature. In order to check the procedure stablished at higher loading %, membranes at 70 % (w/w) were prepared. The MMMs considerably lost their flexibility, being easily cracked. The effectiveness of the preparation of uniform and non-defective MMMs decreased from the 95 % to the 65 %. Thus, 60 % (w/w) was kept as maximum MOF loading for the PCPs adsorption studies.

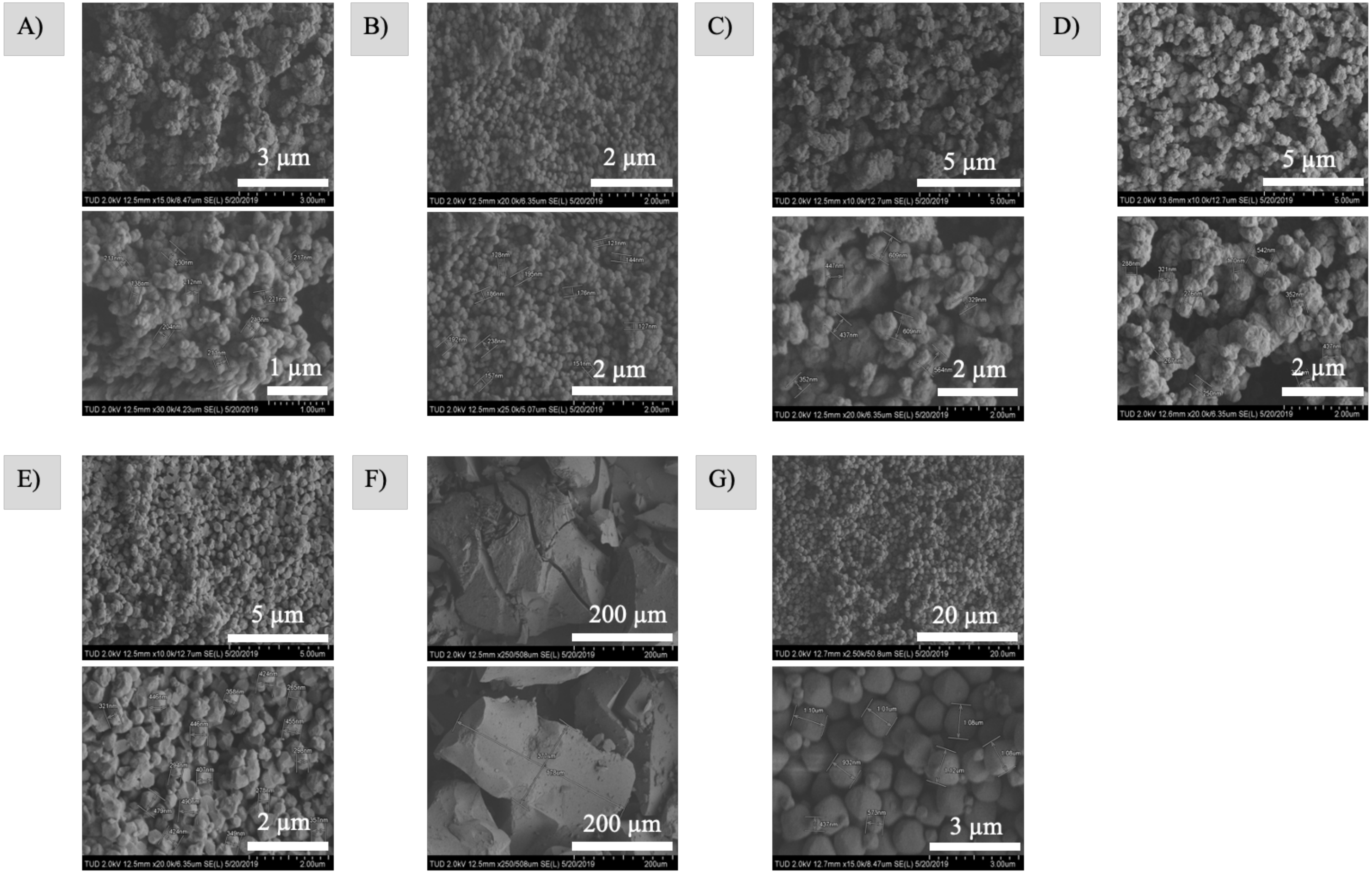

Figure SI.1. SEM images of the neat MOFs **A)** UiO-66 **B)** UiO-66-COOH, **C)** UiO-67, **D)** DUT-52, **E)** DUT-67, **F)** MOF-801 and **G)** MOF-808.

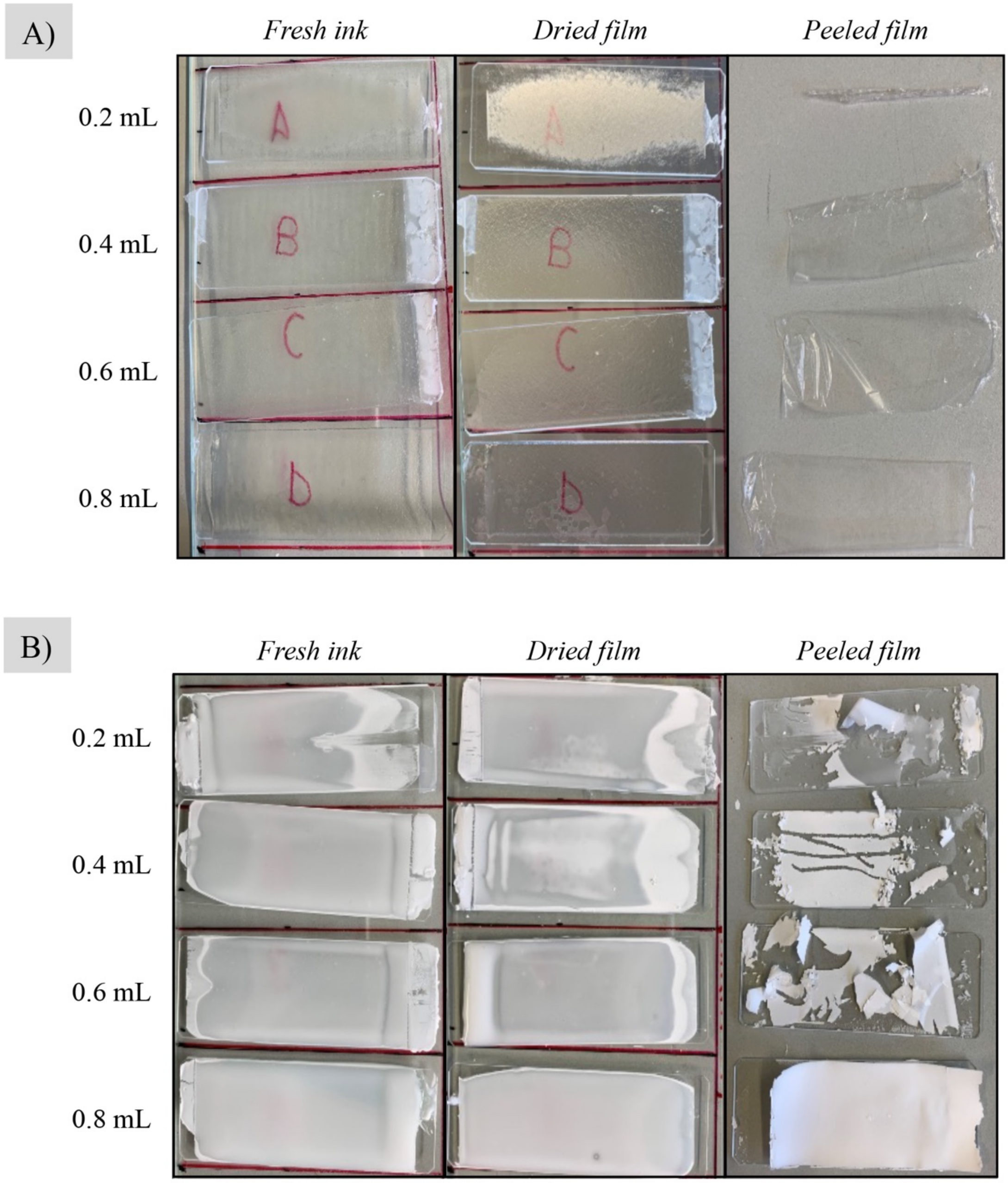

Figure SI.2. Images of the influence of the amount of ink used for the preparation of **A)** neat PVDF, and **B)** PVDF/UiO-66 40% (w/w) of PVDF and 60% (w/w) of MOF MMMs.

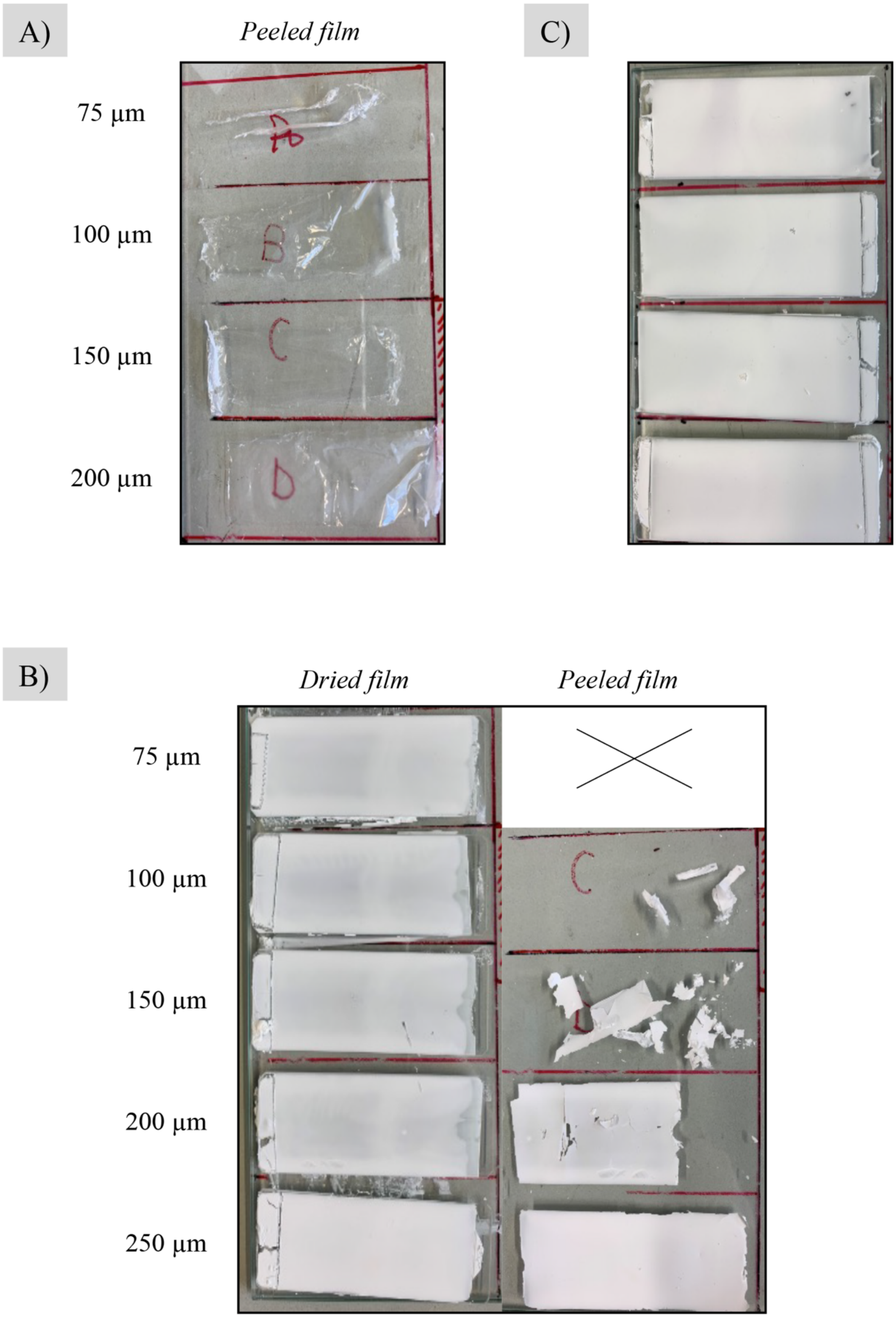

Figure SI.3. Influence of the gap size of the spreading bar (MMMs thickness) for the preparation of **A)** neat PVDF, and **B)** PVDF/UiO-66 (60%, w/w) MMMs. **C)** PVDF/UiO-66 (60%, w/w) prepared under optimized conditions in order to evaluate the reproducibility and effectiveness of the procedure established.

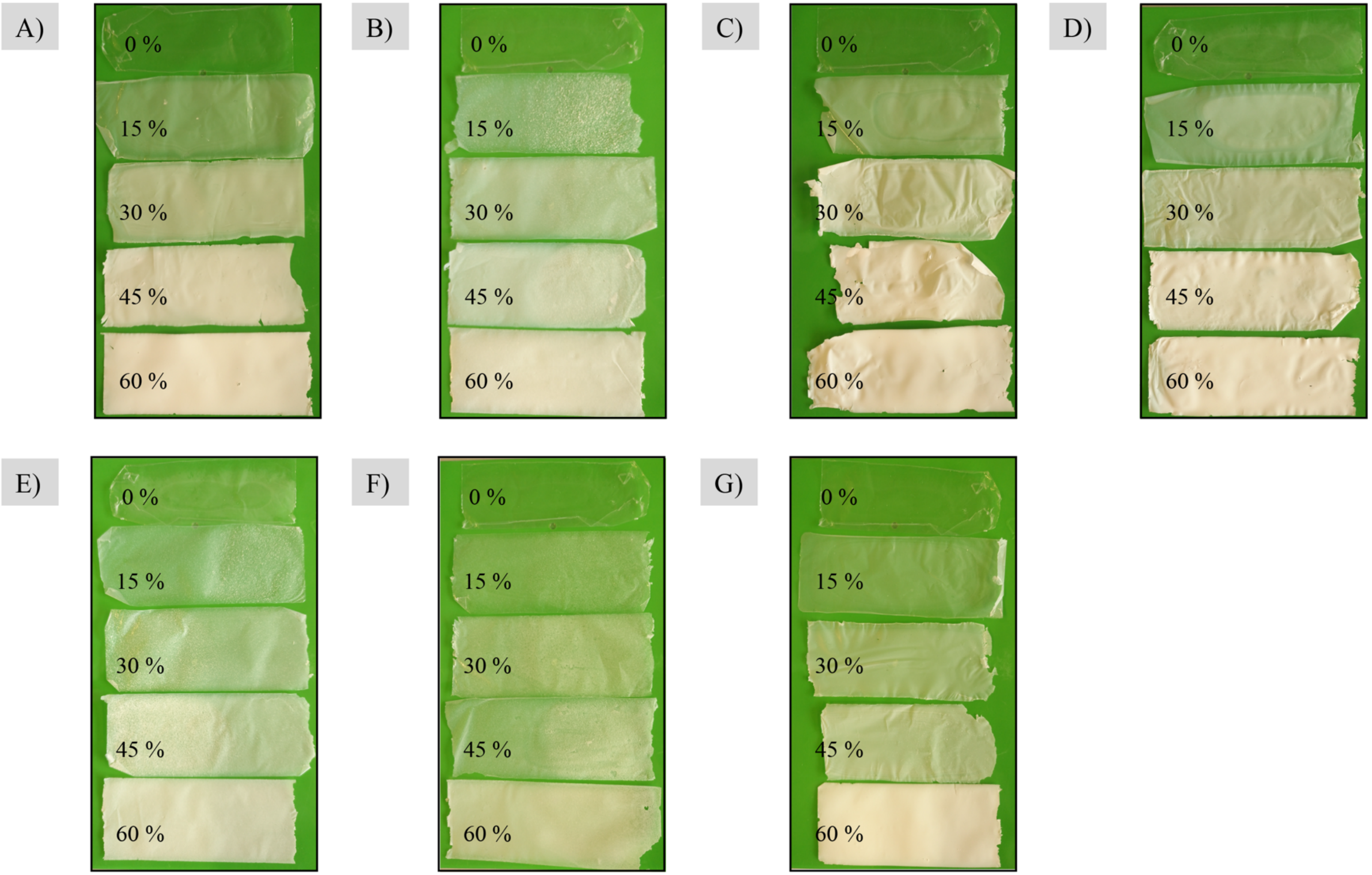

Figure SI.4. Prepared MMMs at different MOF load (%, w/w) of **A)** UiO-66, **B)** UiO-66-COOH, **C)** UiO-67, **D)** DUT-52, **E)** DUT-67, **F)** MOF-801, and **G)** MOF-808

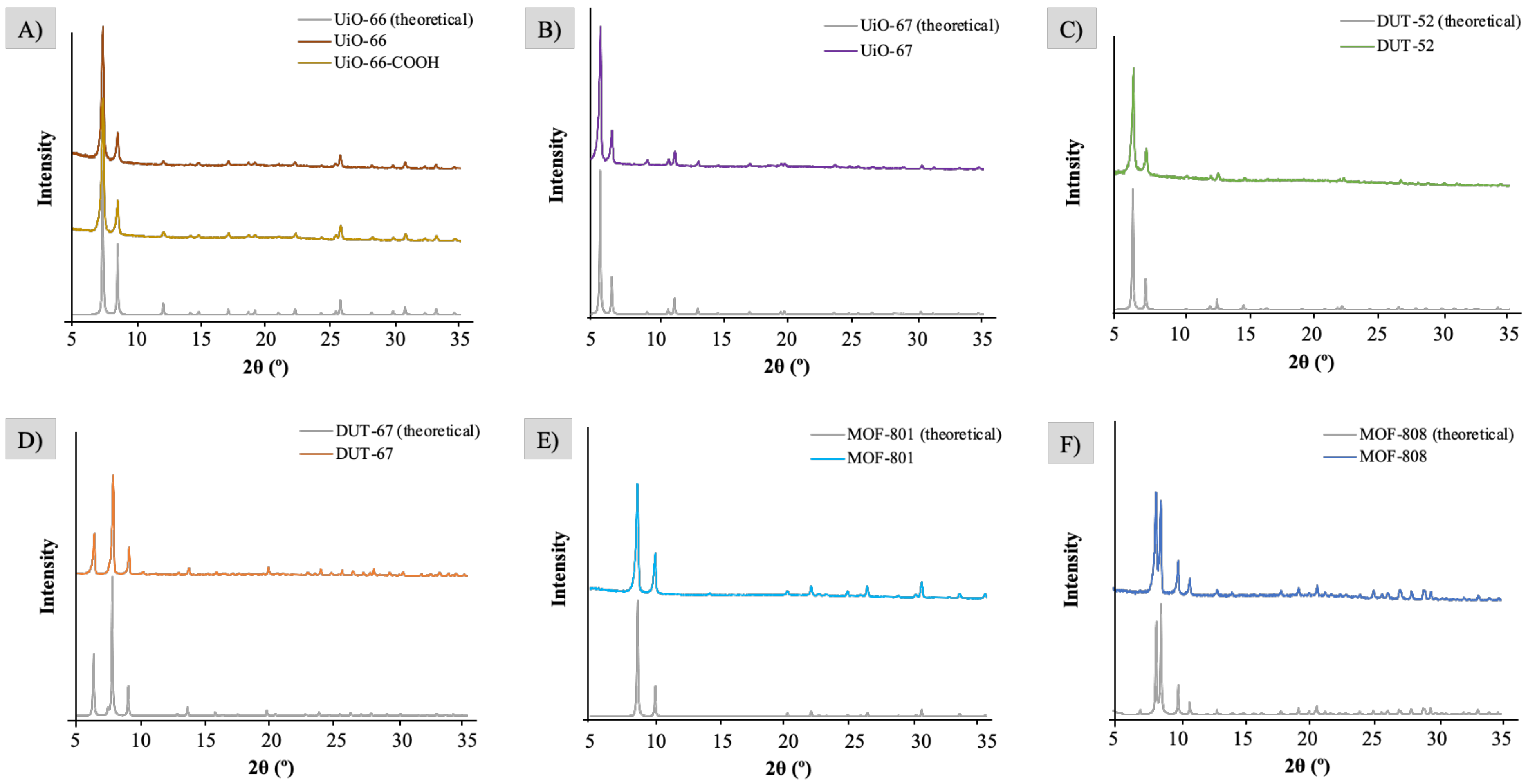

Figure SI.5. Theoretical and experimental powdered XRD patterns of the synthesized **A)** UiO-66 and UiO-66-COOH, **B)** UiO-67, **C)** DUT-52, **D)** DUT-67, **E)** MOF-801, and **F)** MOF-808.

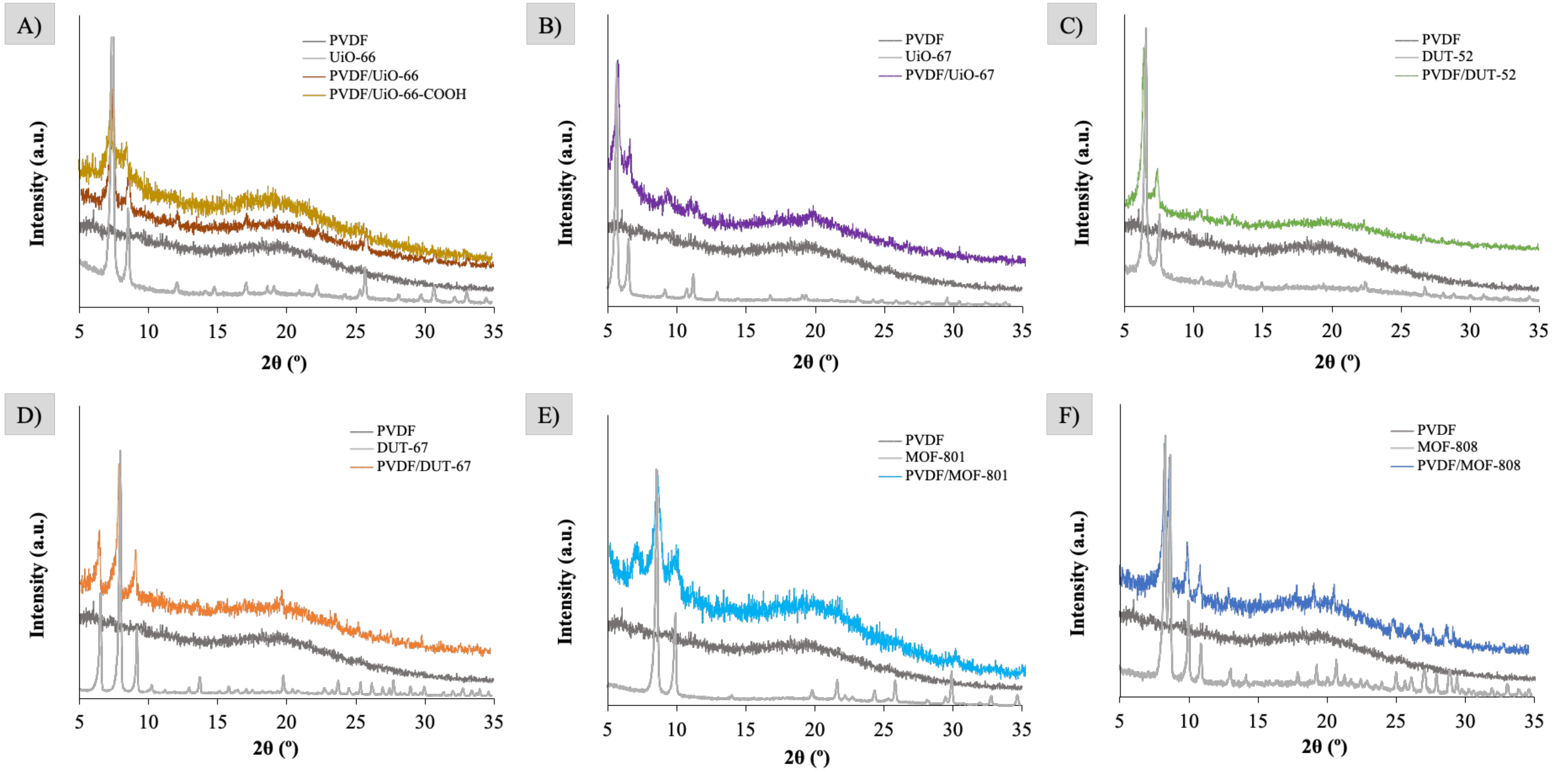

Figure SI.6. Experimental powdered XRD patterns of the prepared **A)** PVDF/UiO-66 and PVDF/UiO-66-COOH, **B)** PVDF/UiO-67, **C)** PVDF/DUT-52, **D)** PVDF/DUT-67, **E)** PVDF/MOF-801, and **F)** PVDF/MOF-808 MMMs.

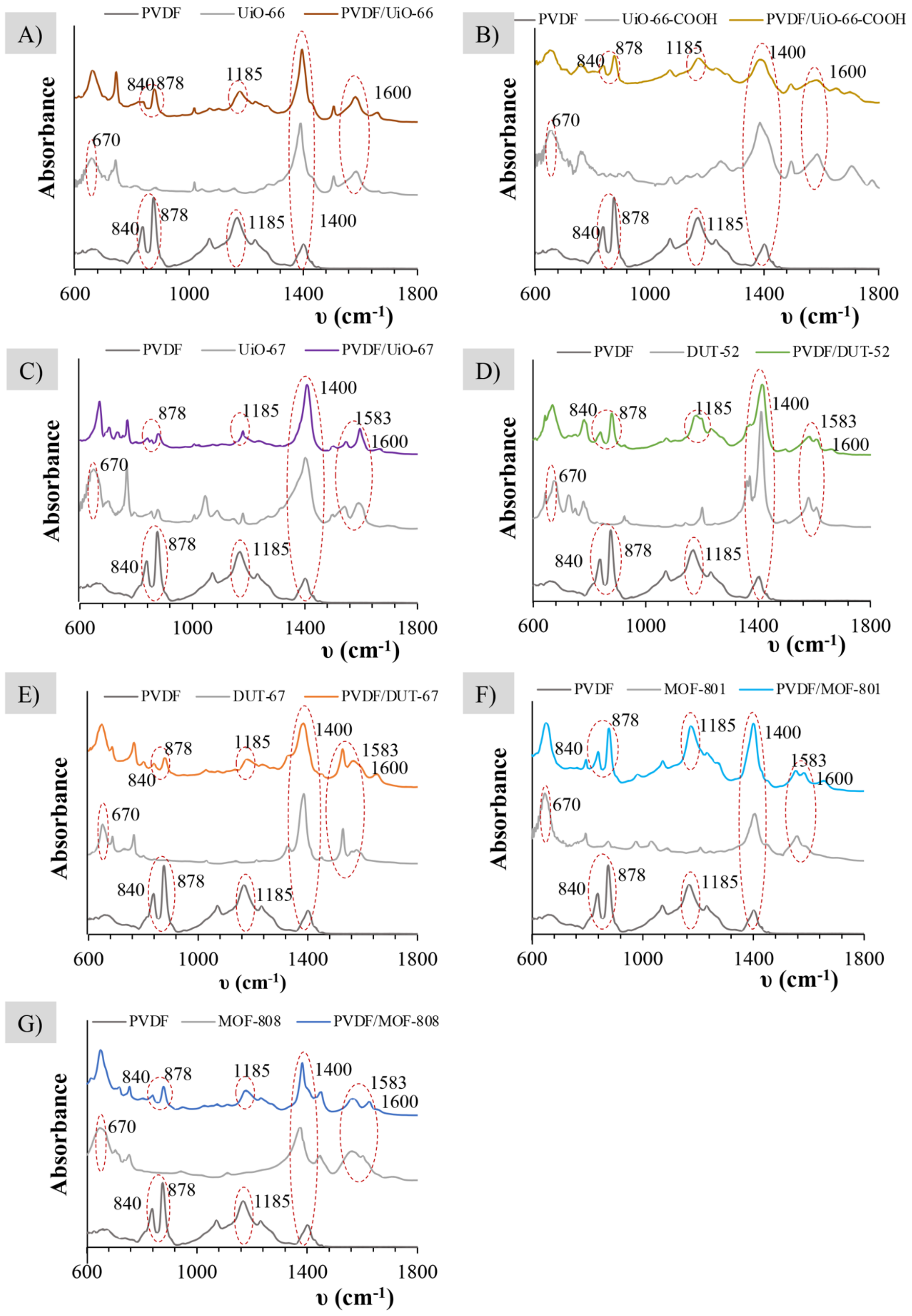

Figure SI.7. FT-IR spectra of the polymer, the neat MOFs, and MOF-MMMs: **A)** UiO-66, **B)** UiO-66-COOH, **C)** UiO-67, **D)** DUT-52, E) DUT-67, **F)** MOF-801, and **G)** MOF-808.

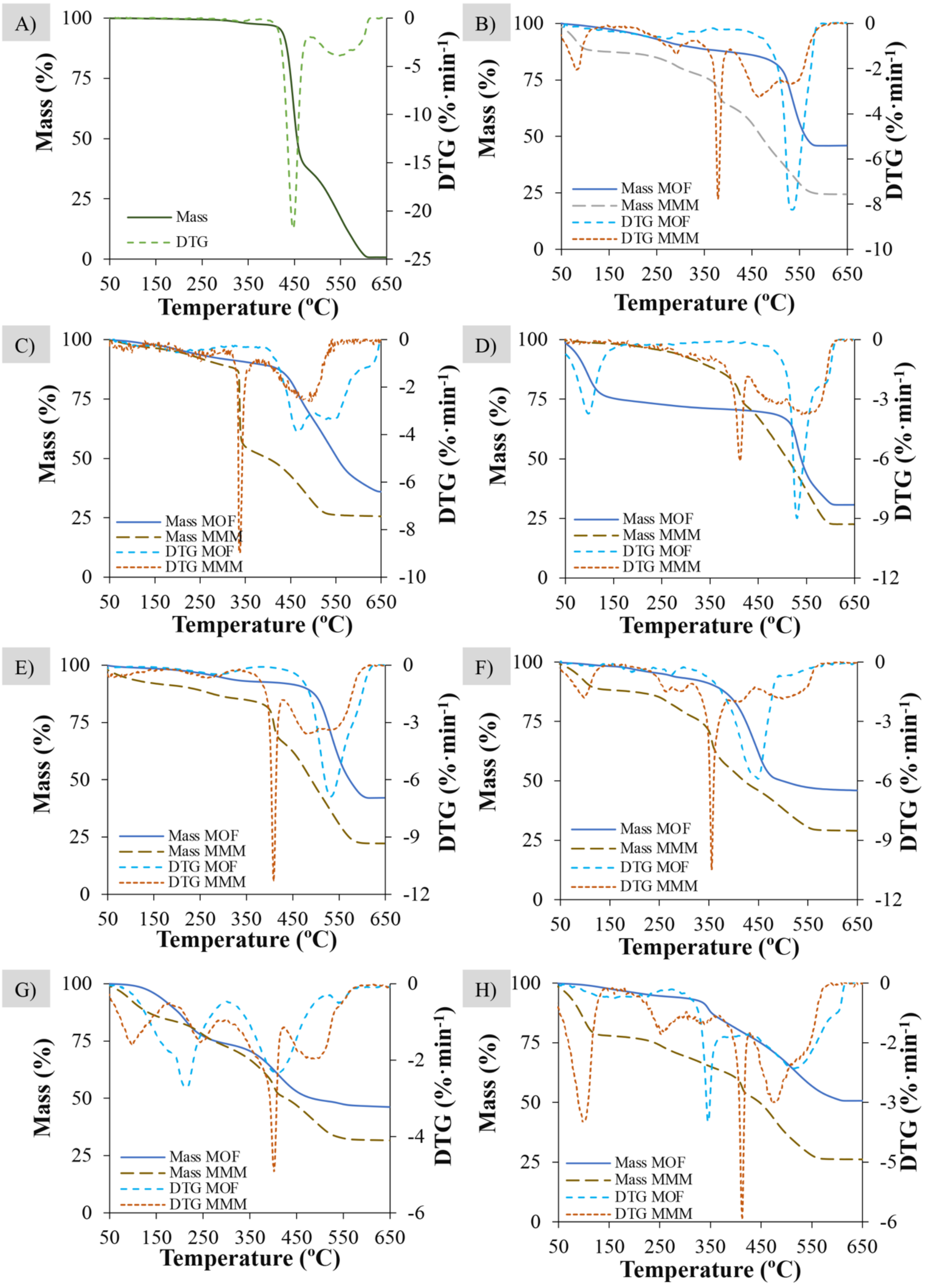

Figure SI.8. TGA in air atmosphere of **A)** PVDF **B)** UiO-66, **C)** UiO-66-COOH, **D)** UiO-67, **E)** DUT-52, **F)** DUT-67, **G)** MOF-801, and **H)** MOF-808, and their MOF-MMMs at 60% (w/w).

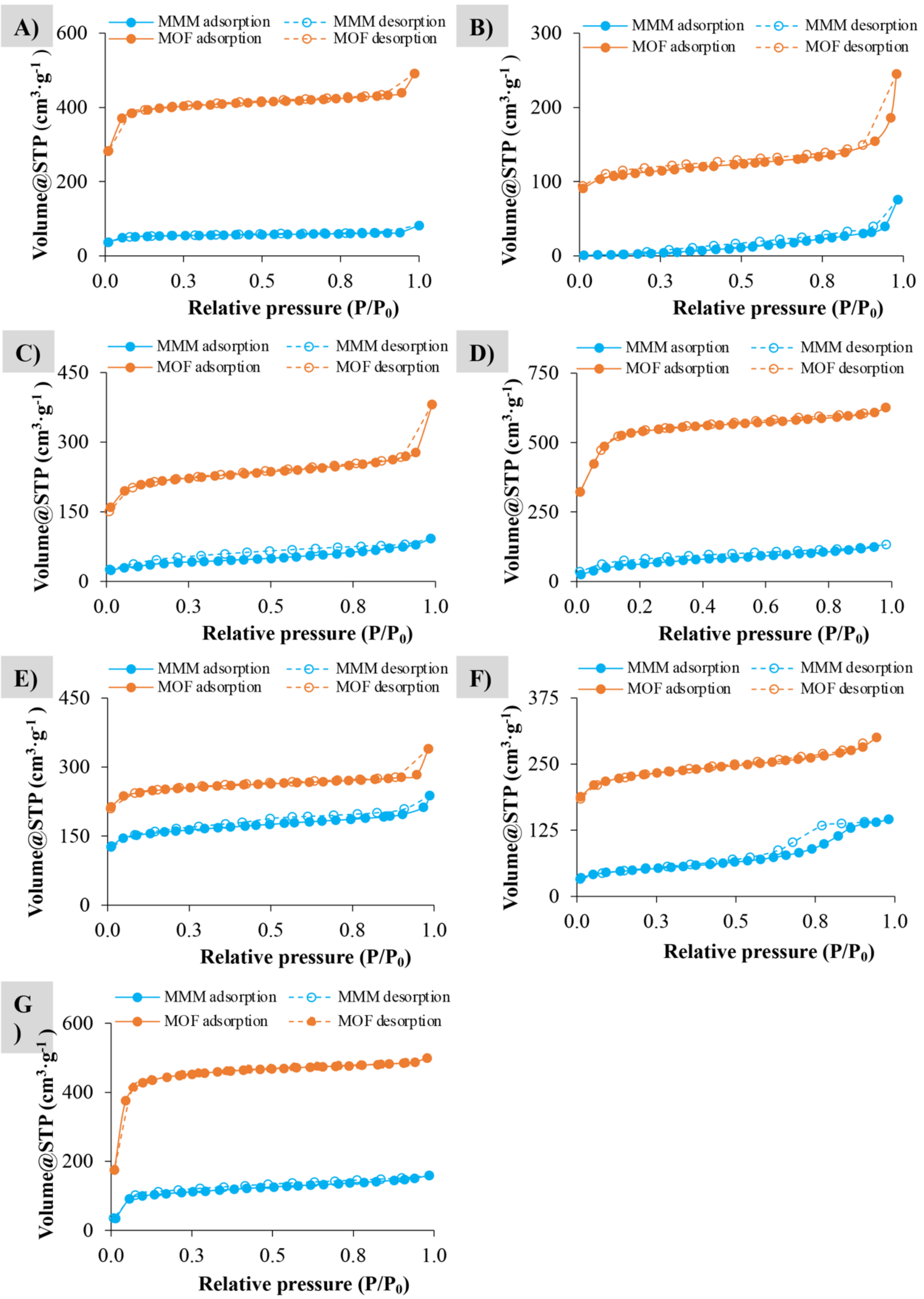

Figure SI.9. N_2 adsorption and desorption isotherms at 77 K of neat MOFs and MOF-based MMMs of: **A)** UiO-66, **B)** UiO-66-COOH, **C)** UiO-67, **D)** DUT-52, **E)** DUT-67, **F)** MOF-801, and **G)** MOF-808.

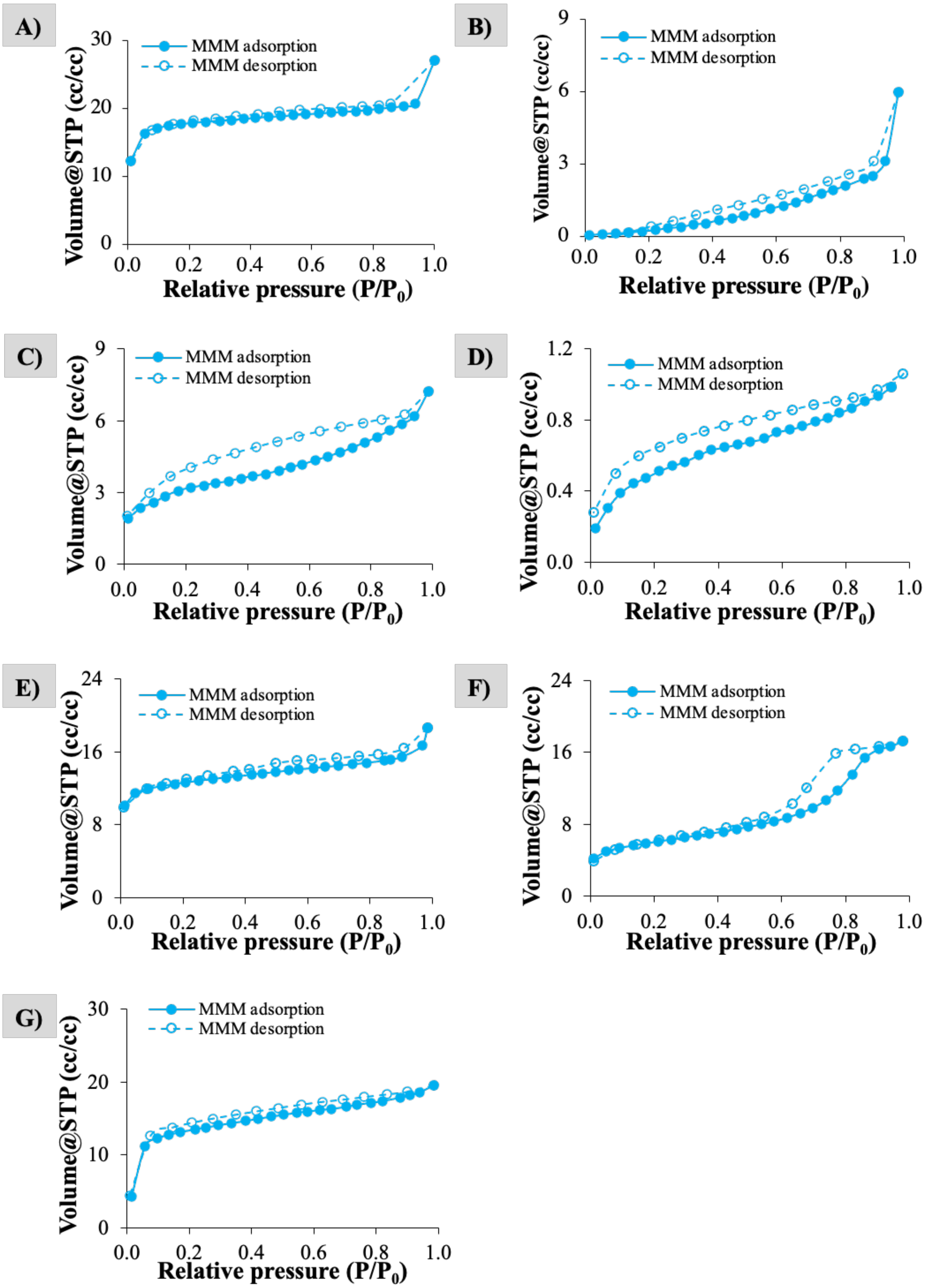

Figure SI.10. N_2 adsorption and desorption isotherms at 77 K of neat MOFs and MOF-based MMMs of: **A)** UiO-66, **B)** UiO-66-COOH, **C)** UiO-67, **D)** DUT-52, **E)** DUT-67, **F)** MOF-801, and **G)** MOF-808.

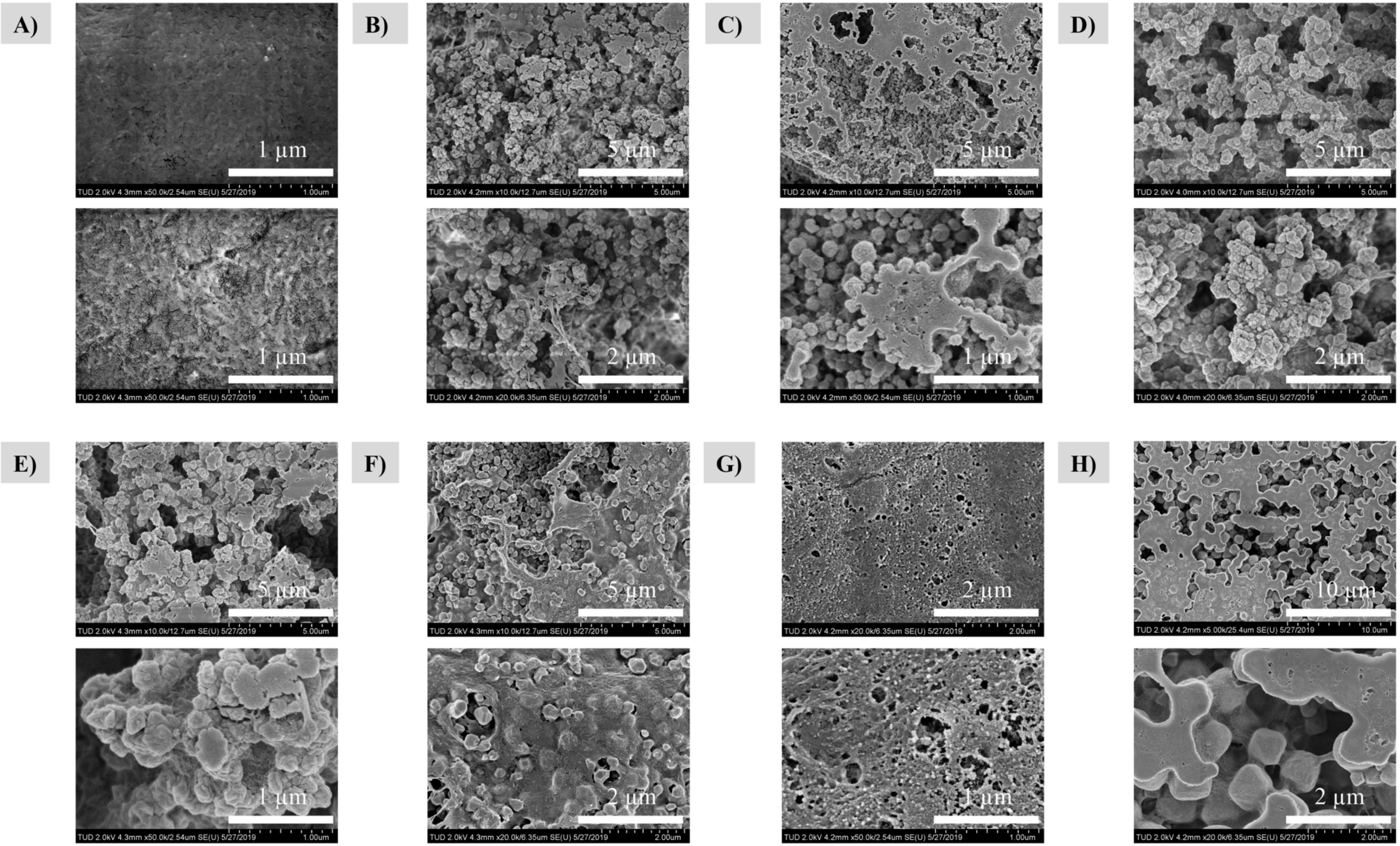

Figure SI.11. SEM images of the prepared **A)** PVDF, **B)** PVDF/UiO-66, **C)** PVDF/UiO-66-COOH, **D)** PVDF/UiO-67, **E)** PVDF/DUT-52, **F)** PVDF/DUT-67, **G)** PVDF/MOF-801, and **H)** PVDF/MOF-808 MMMs.

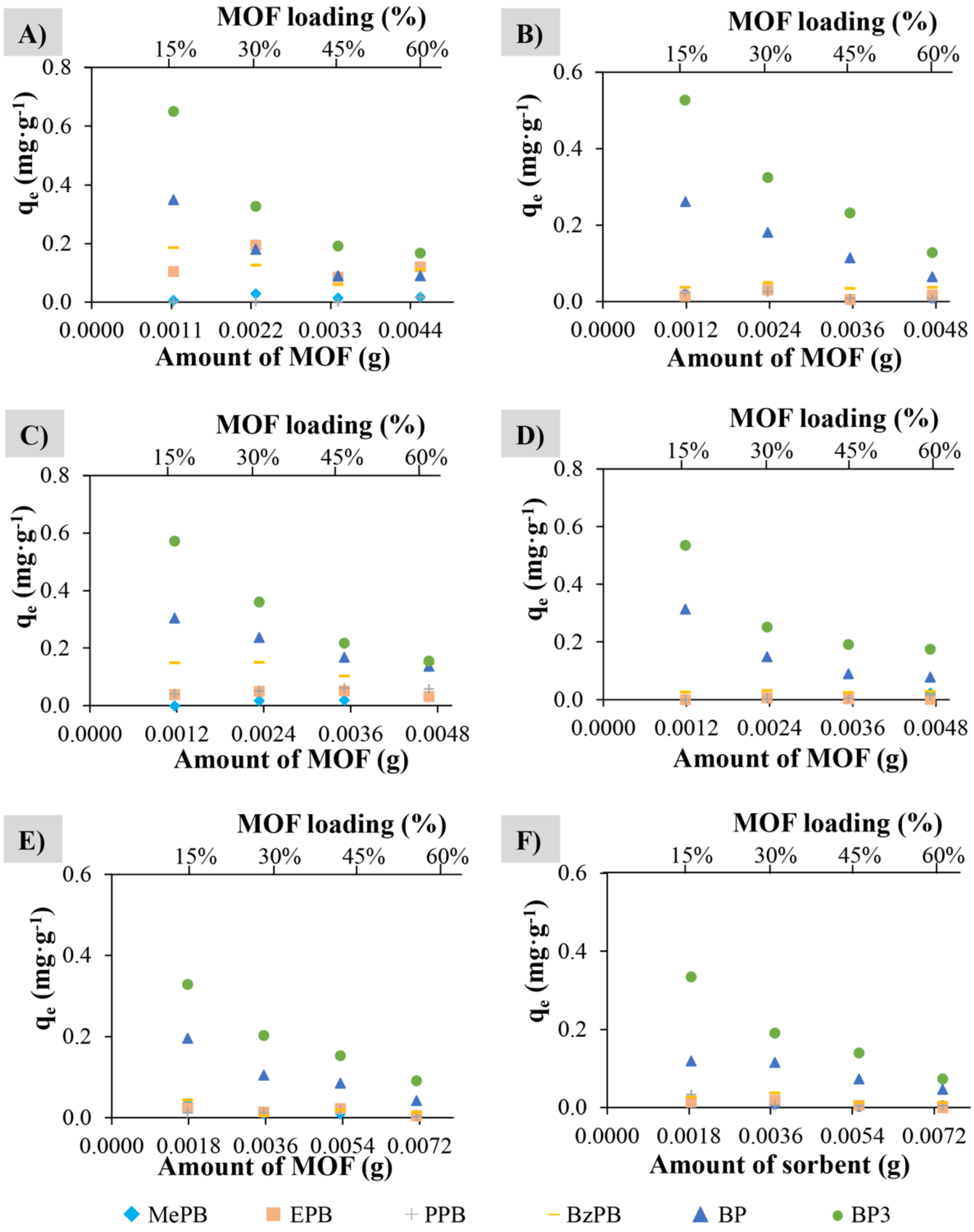

Figure SI.12. Influence of MOF loadings in the MMMs for the studies of PCPs adsorption using: **A)** PVDF/UiO-66, **B)** PVDF/UiO-66-COOH, **C)** PVDF/UiO-67, **D)** PVDF/DUT-67, **E)** PVDF/MOF-801, and **F)** PVDF/MOF-808.

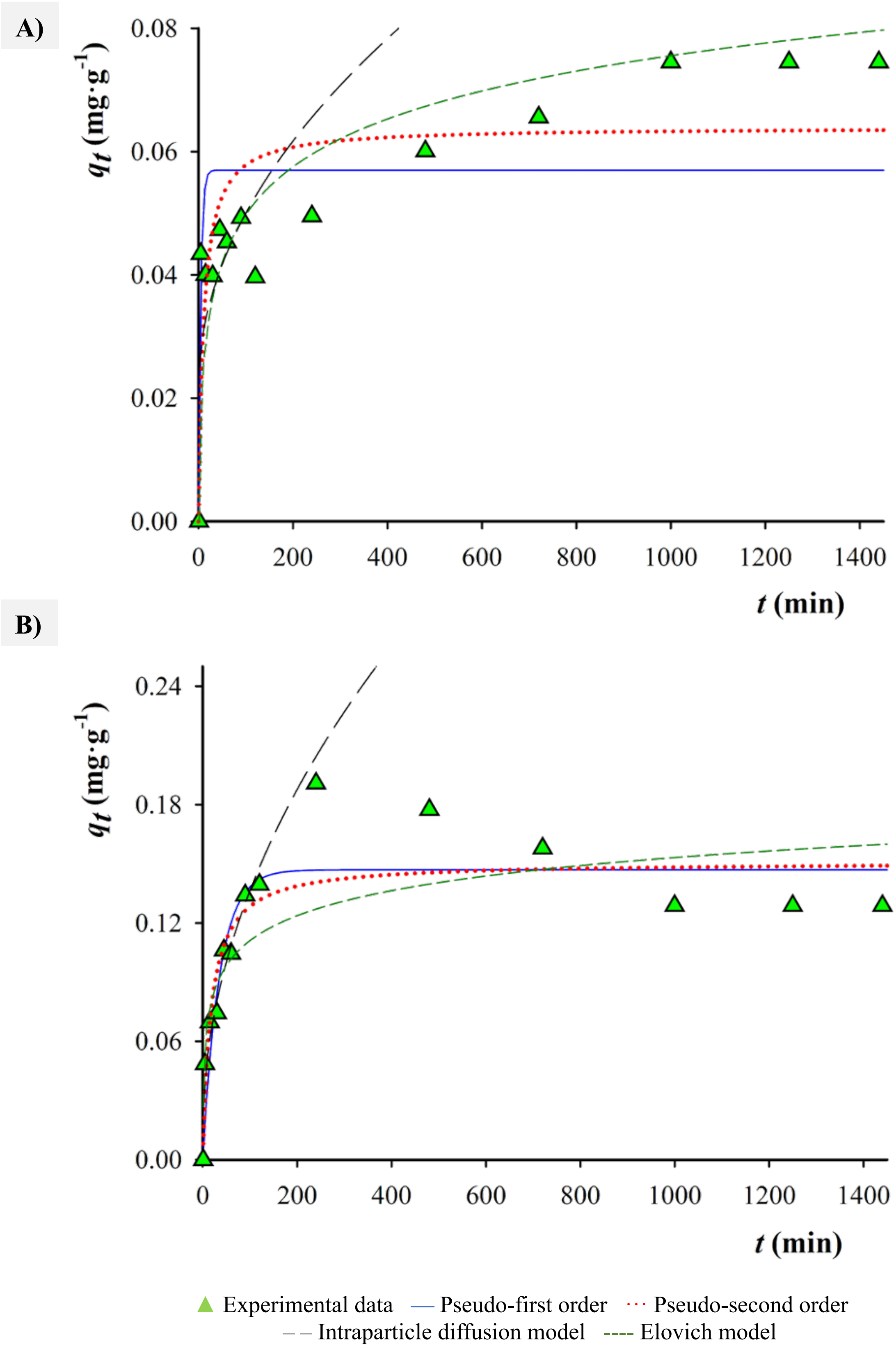

Figure SI.13. Kinetics of **A)** MePB, **B)** BzPB, **C)** BP, and **D)** BP3 adsorption into PVDF/UiO-66, fitted through pseudo-first order, pseudo-second order, interparticle diffusion, and Elovich models.

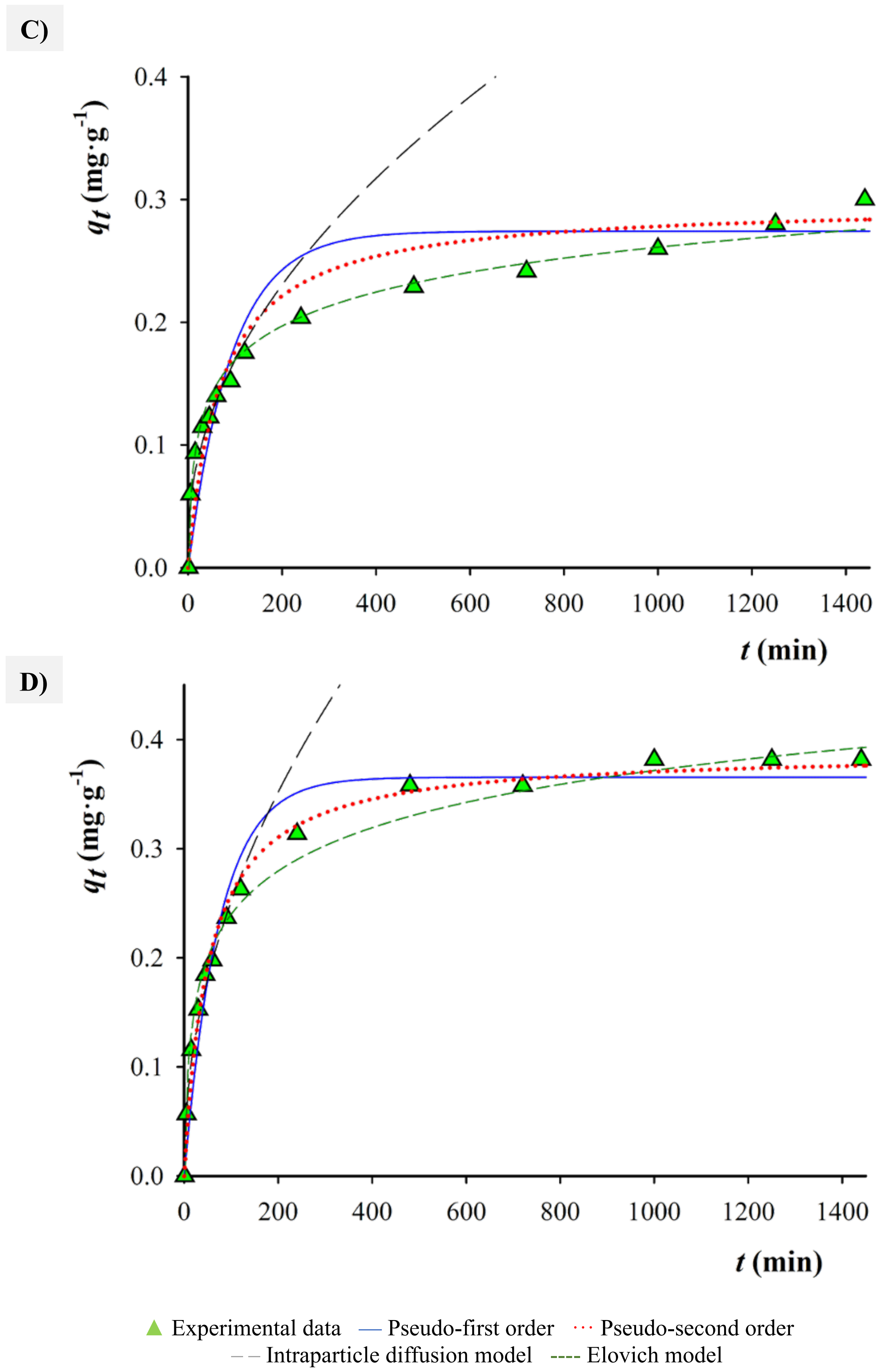

Figure SI.13.(continued)

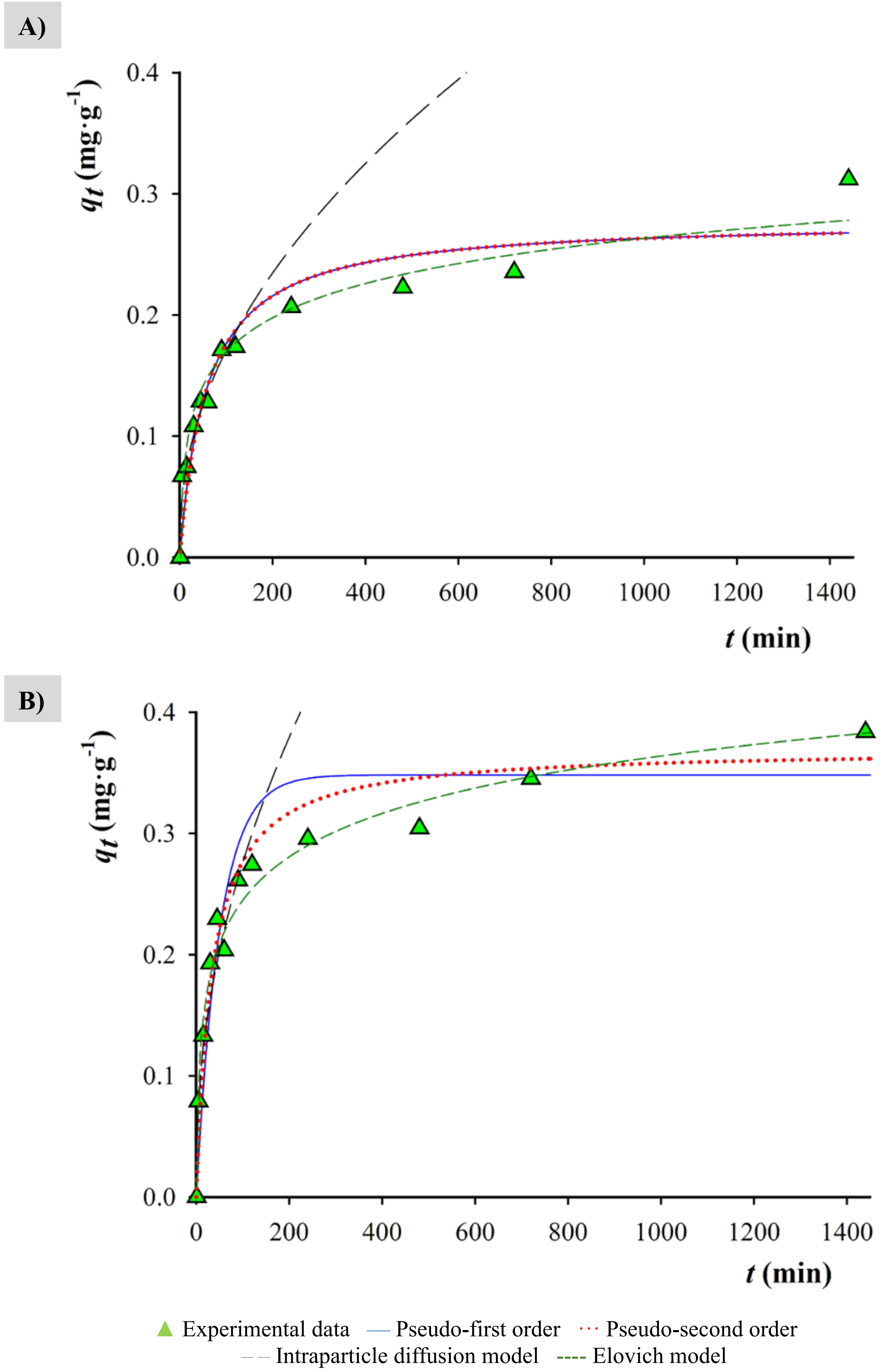

Figure SI.14. Kinetics of **A)** BP, and **B)** BP3 adsorption into PVDF/UiO-66-COOH, fitted through pseudo-first order, pseudo-second order, interparticle diffusion, and Elovich models.

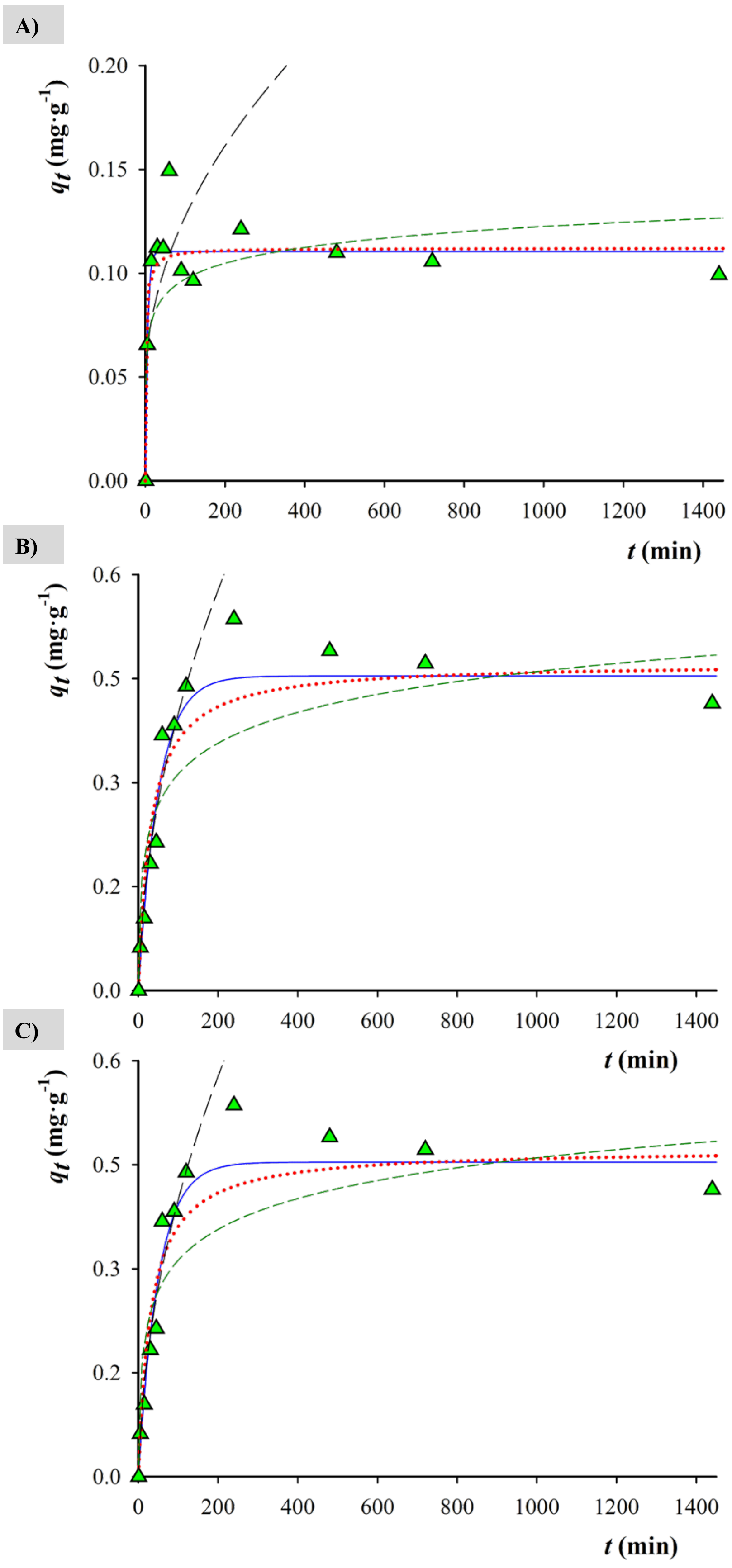

Figure SI.15. Kinetics of **A)** MePB, **B)** EPB, **C)** BzPB, **D)** BP, and **E)** BP3 adsorption into PVDF/UiO-67, fitted through pseudo-first order, pseudo-second order, interparticle diffusion, and Elovich models.

D)

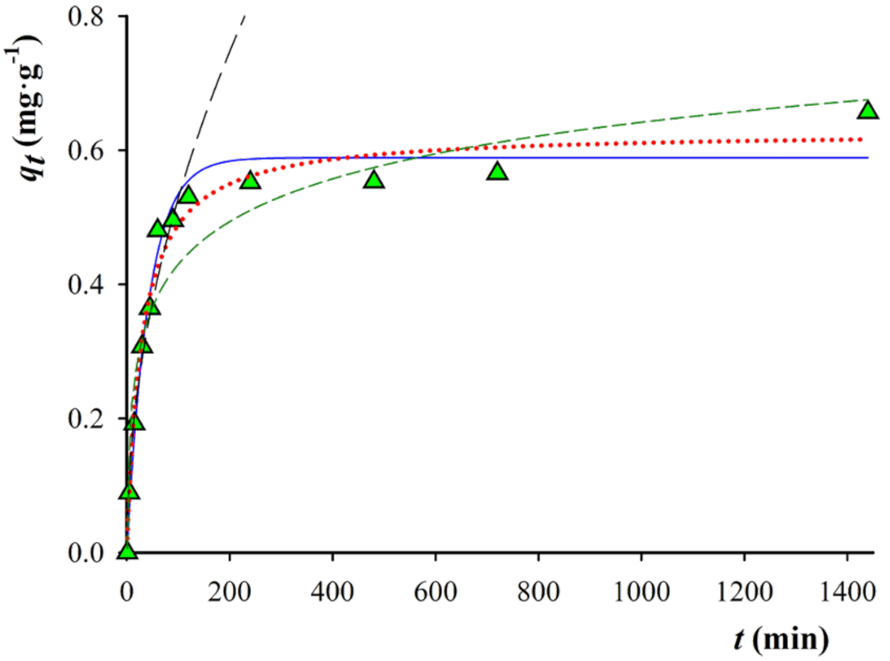

E)

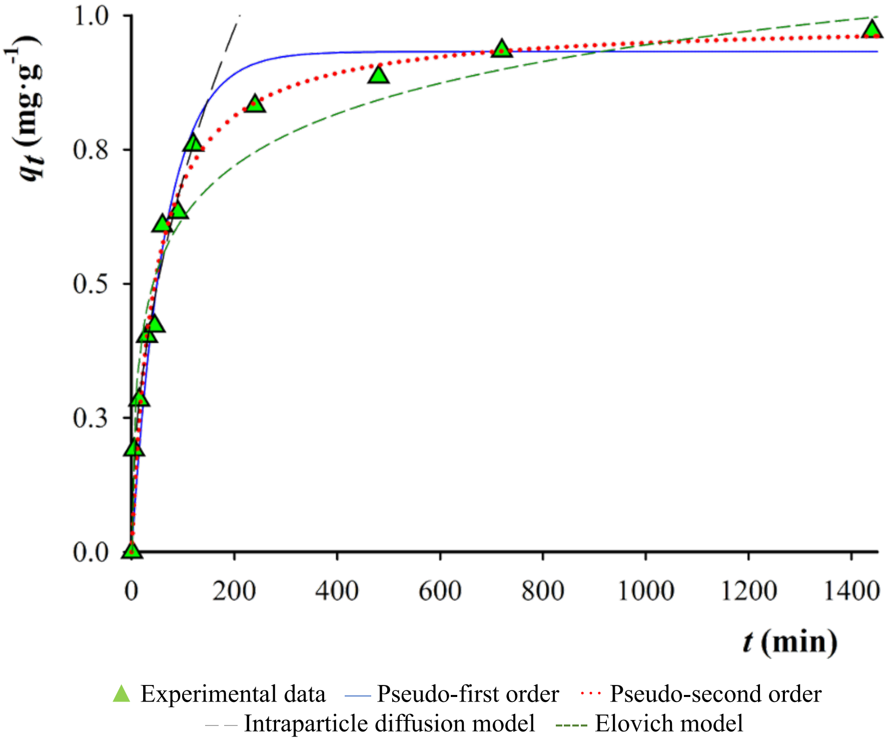

Figure SI.16.(continued)

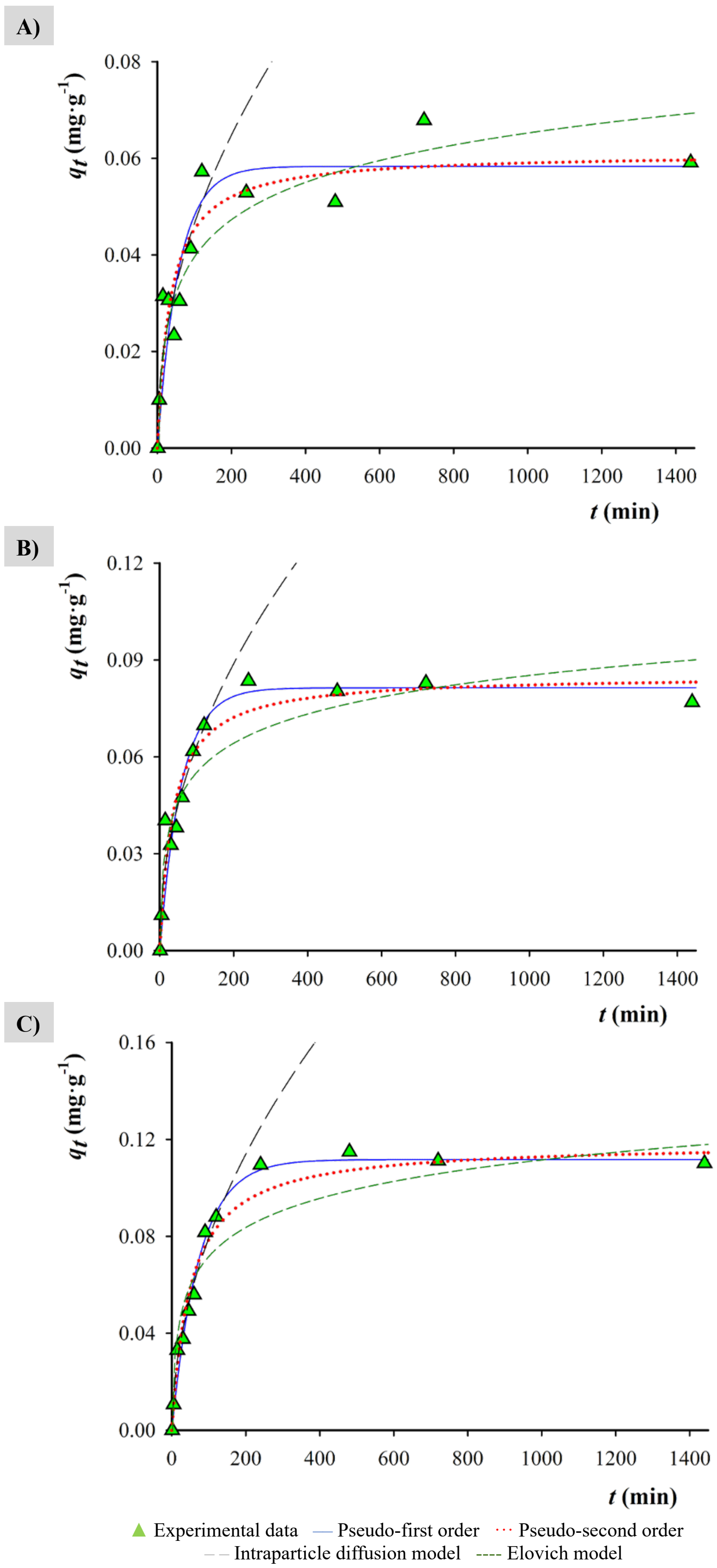

Figure SI.16. Kinetics of **A)** MePB, **B)** EtPB, **C)** PPB, **D)** BzPB, **E)** BP, and **F)** BP3 adsorption into PVDF/DUT-52, fitted through pseudo-first order, pseudo-second order, intraparticle diffusion, and Elovich models.

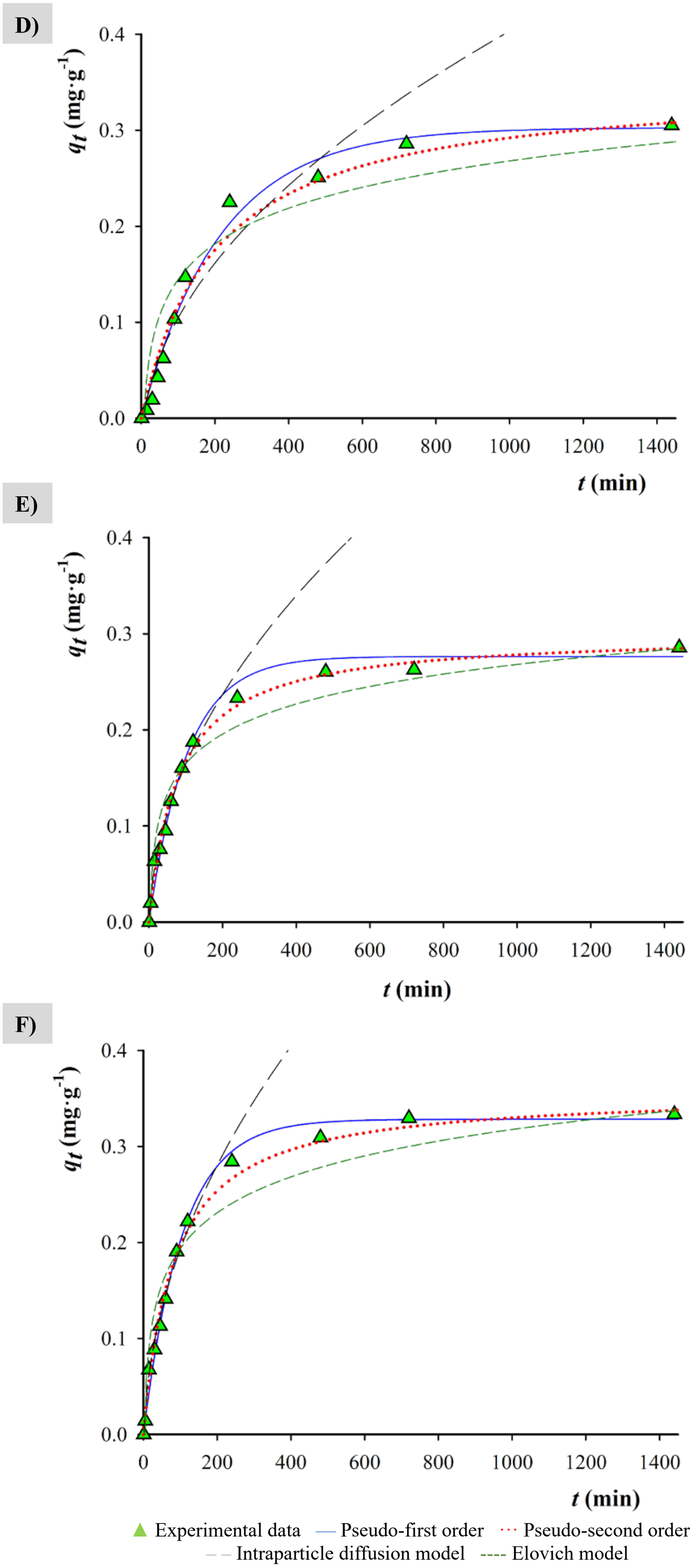

Figure SI.16.(continued)

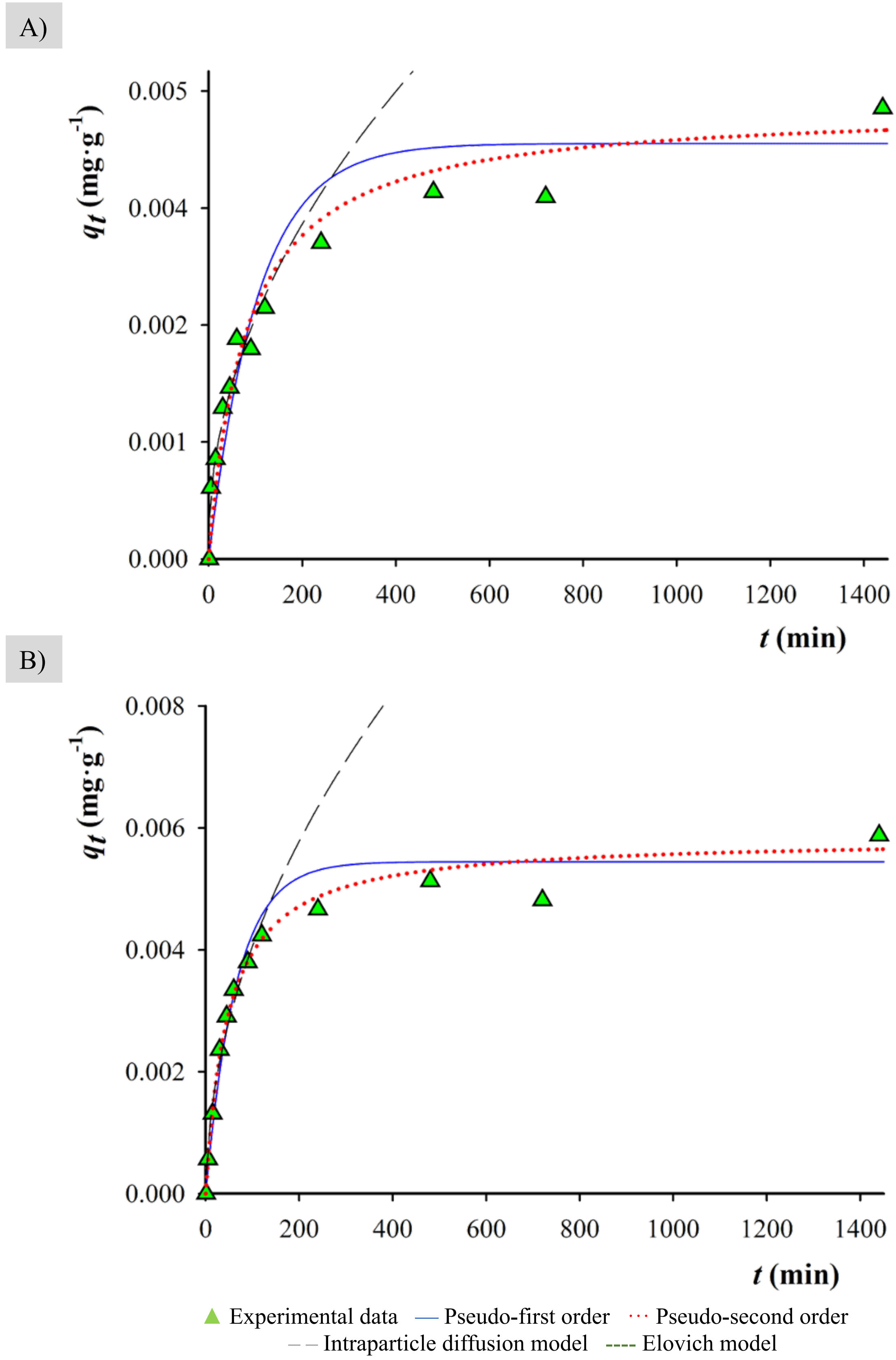

Figure SI.17. Kinetics of **A)** BP, and **B)** BP3 adsorption into PVDF/DUT-67, fitted through pseudo-first order, pseudo-second order, and interparticle diffusion models.

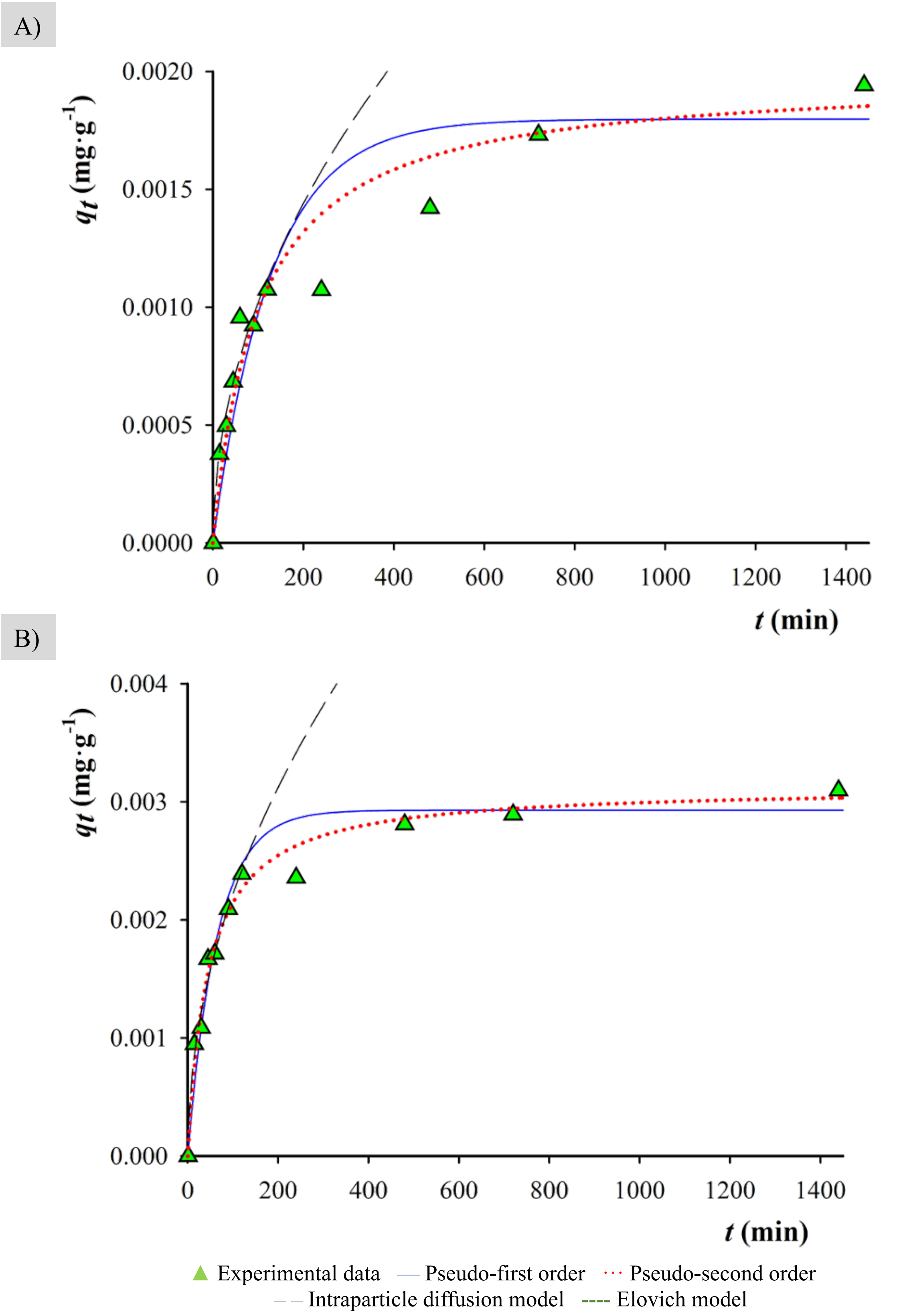

Figure SI.18. Kinetics of **A)** BP, and **B)** BP3 adsorption into PVDF/MOF-801, fitted through pseudo-first order, pseudo-second order, and interparticle diffusion models.

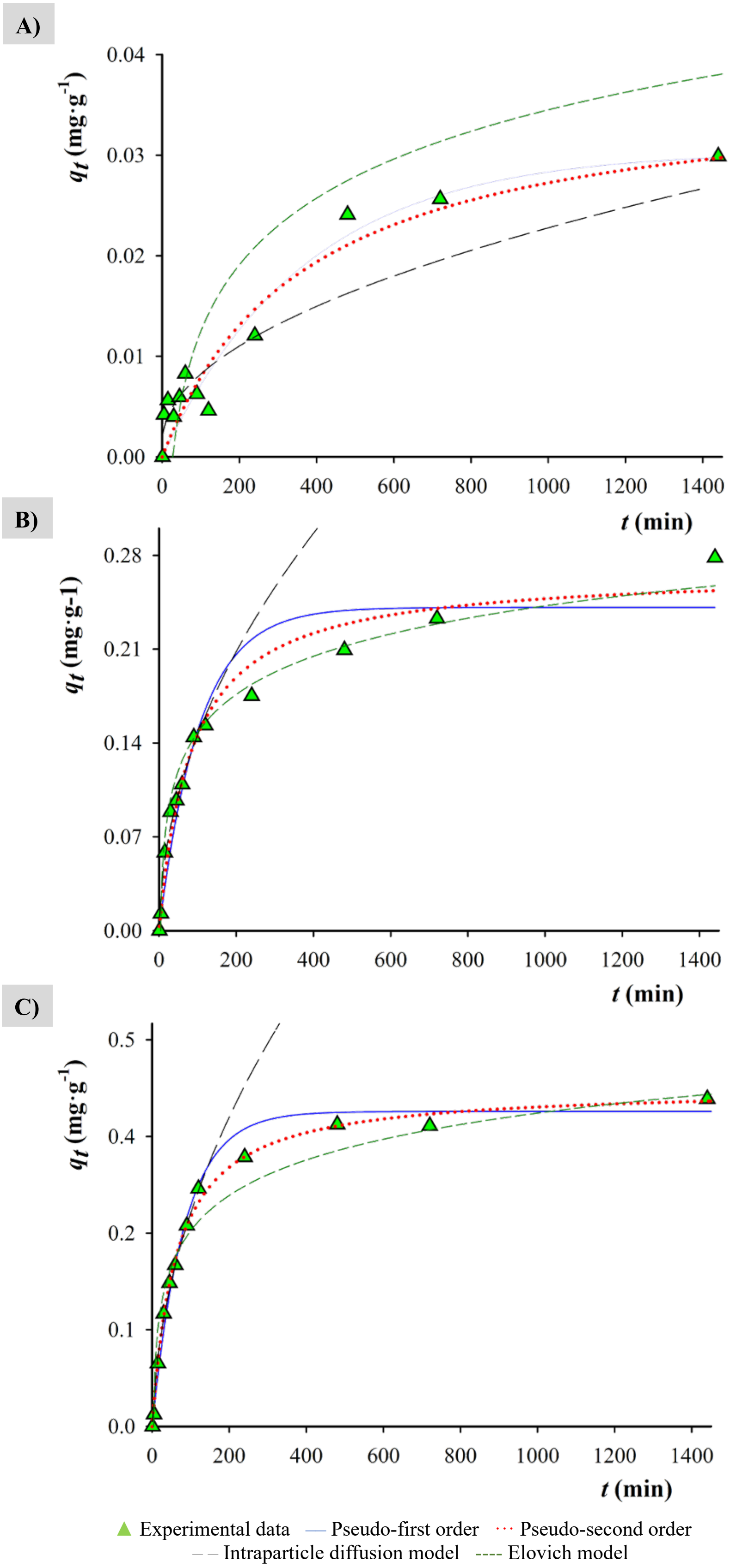

Figure SI.19. Kinetics of **A)** MePB, **B)** BP, and **C)** BP3 adsorption into PVDF/MOF-808, fitted through pseudo-first order, pseudo-second order, interparticle diffusion, and Elovich models.

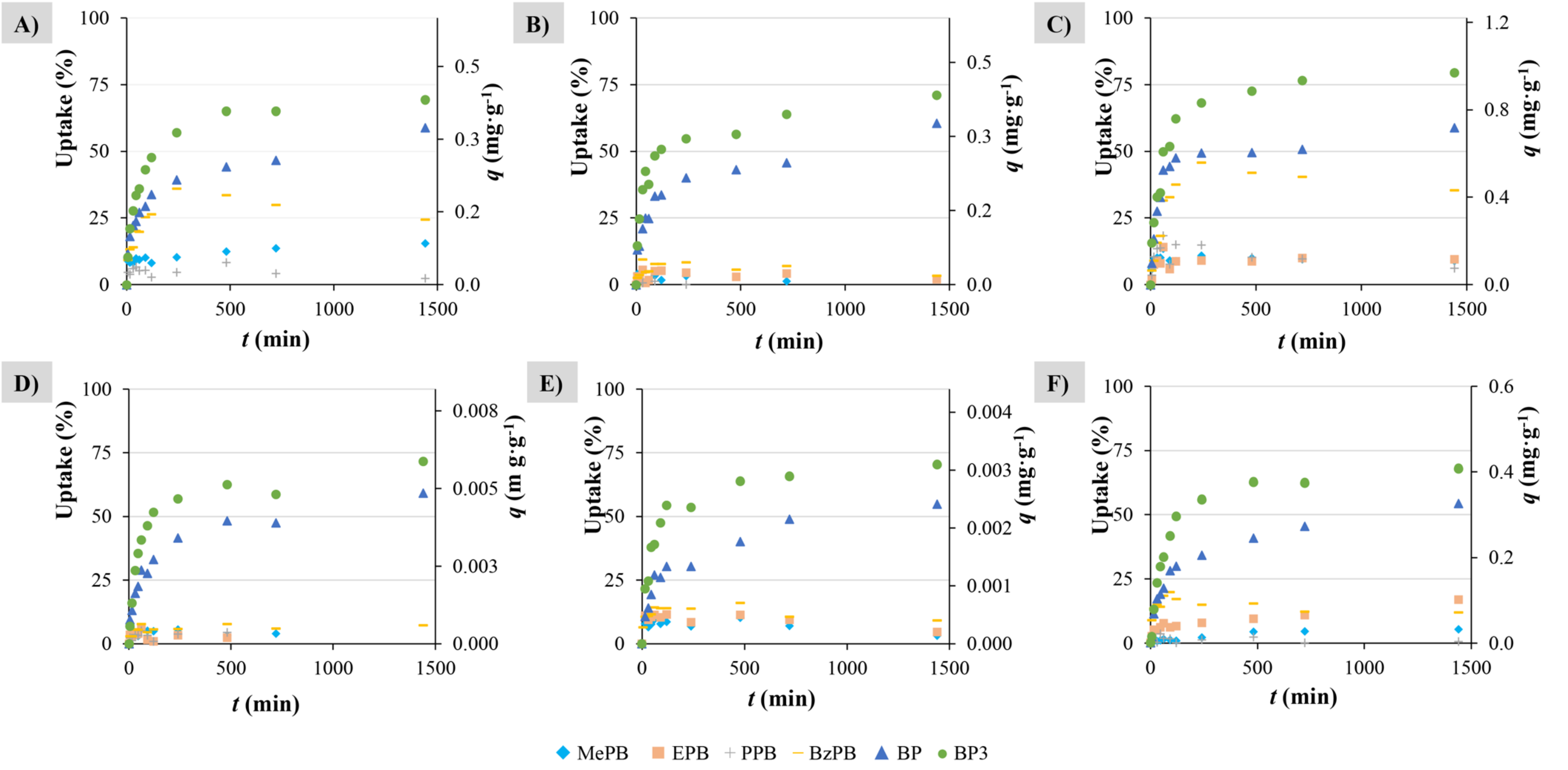

Figure SI.20. Uptake (expressed as (%) and q ($mg \cdot g^{-1}$)) of the six PCPs for: **A)** PVDF/UiO-66, **B)** PVDF/UiO-66-COOH, **C)** PVDF/UiO-67, **D)** PVDF/DUT-67, **E)** PVDF/MOF-801, and **F)** PVDF/MOF-808 MMMs.

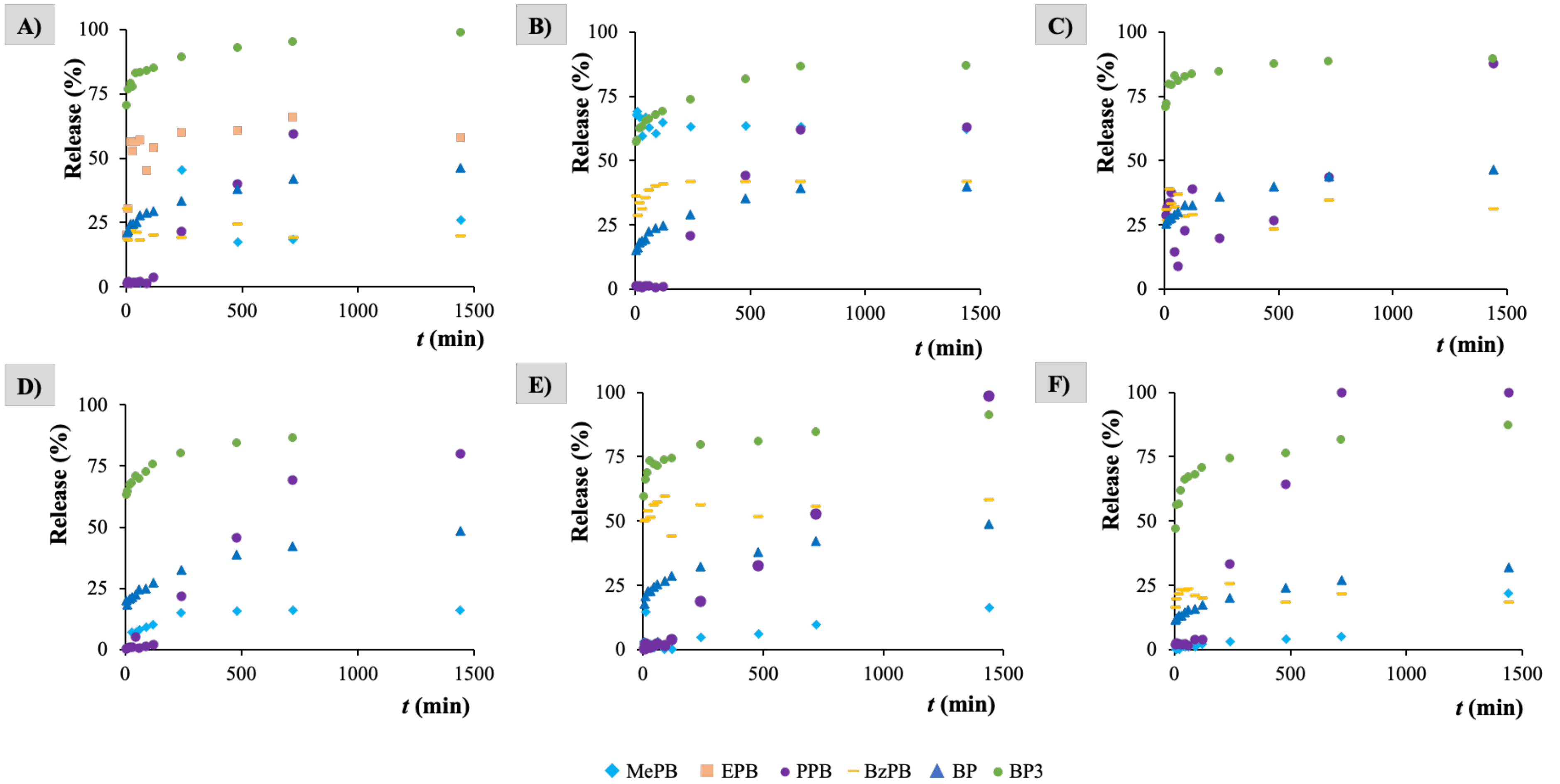

Figure SI.21. Release (in %) of the six PCPs for: **A)** PVDF/UiO-66, **B)** PVDF/UiO-66-COOH, **C)** PVDF/UiO-67, **D)** PVDF/DUT-67, **E)** PVDF/MOF-801, and **F)** PVDF/MOF-808 MMMs.

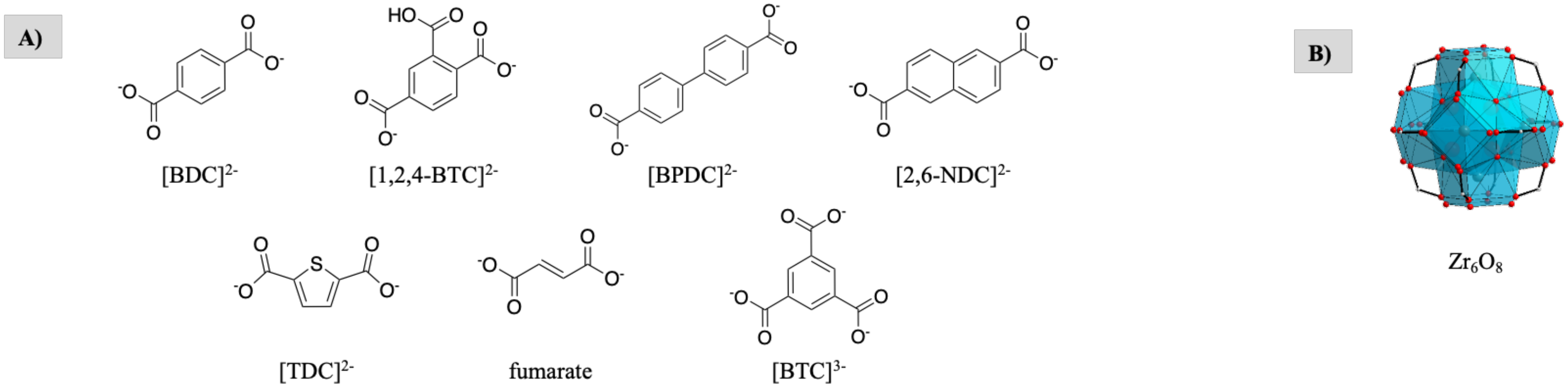

C)

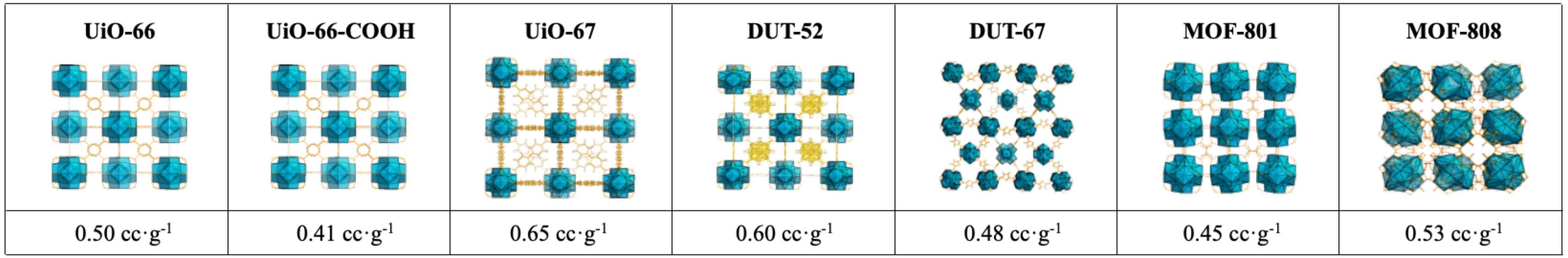

	UiO-66	UiO-66-COOH	UiO-67	DUT-52	DUT-67	MOF-801	MOF-808
Pore volume	0.50 cc·g^{-1}	0.41 cc·g^{-1}	0.65 cc·g^{-1}	0.60 cc·g^{-1}	0.48 cc·g^{-1}	0.45 cc·g^{-1}	0.53 cc·g^{-1}

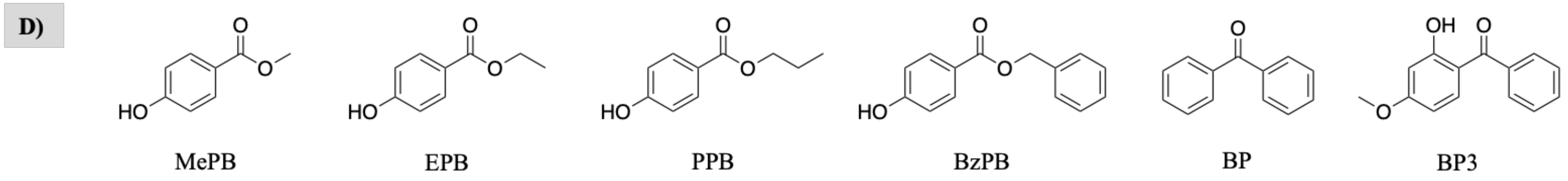

Figure SI.22. Scheme of **A**) ligands of the MOFs, **B)** MOFs metal nodes, **C)** structures and the pores volume of the MOFs, and **D)** the structure of the PCPs studied.

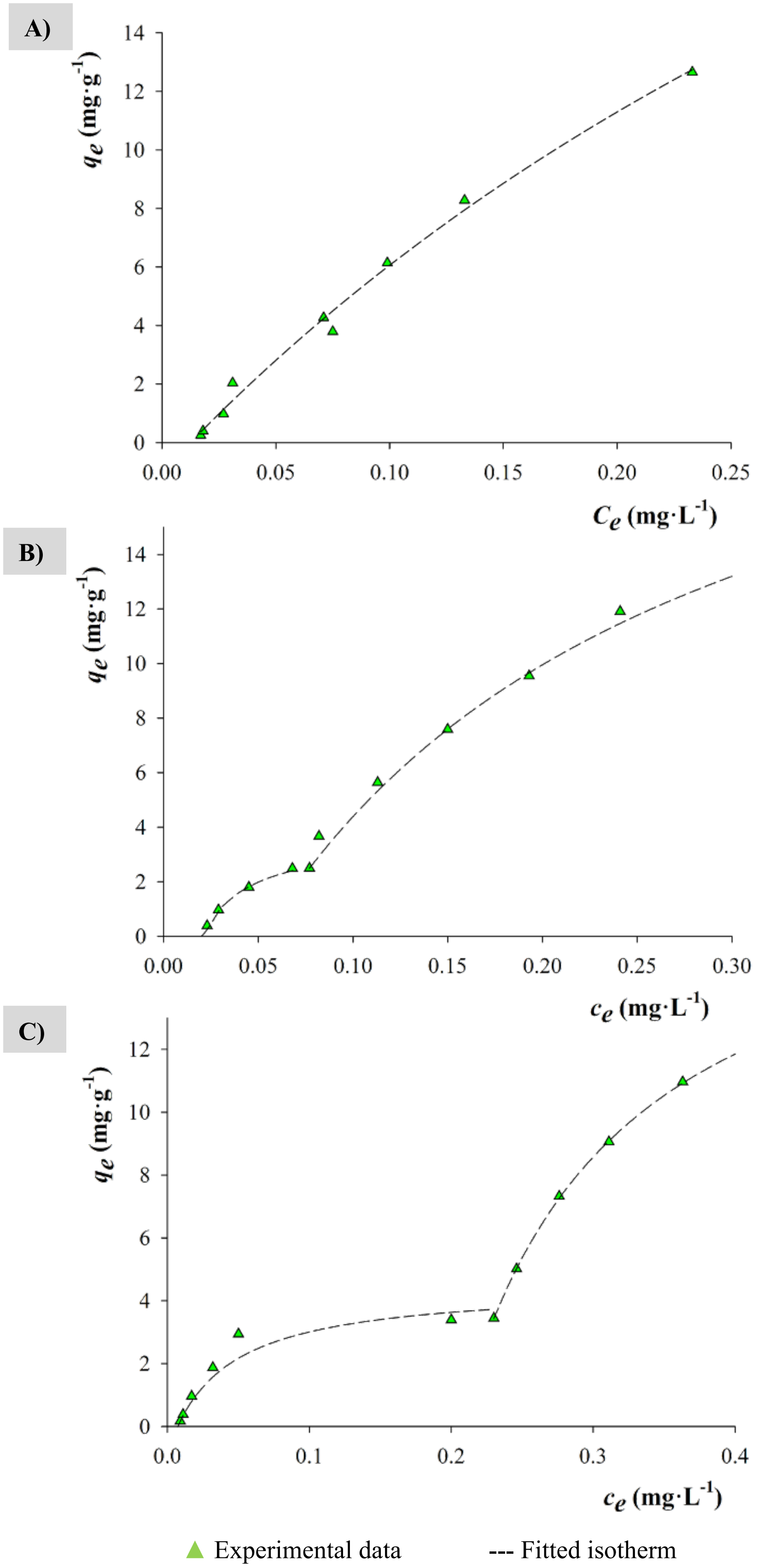

Figure SI.23. Adsorption isotherms of BP3 for: **A)** PVDF/UiO-66, **B)** PVDF/UiO-66-COOH, **C)** PVDF/UiO-67, **D)** PVDF/DUT-67, **E)** PVDF/MOF-801, and **F)** PVDF/MOF-808.

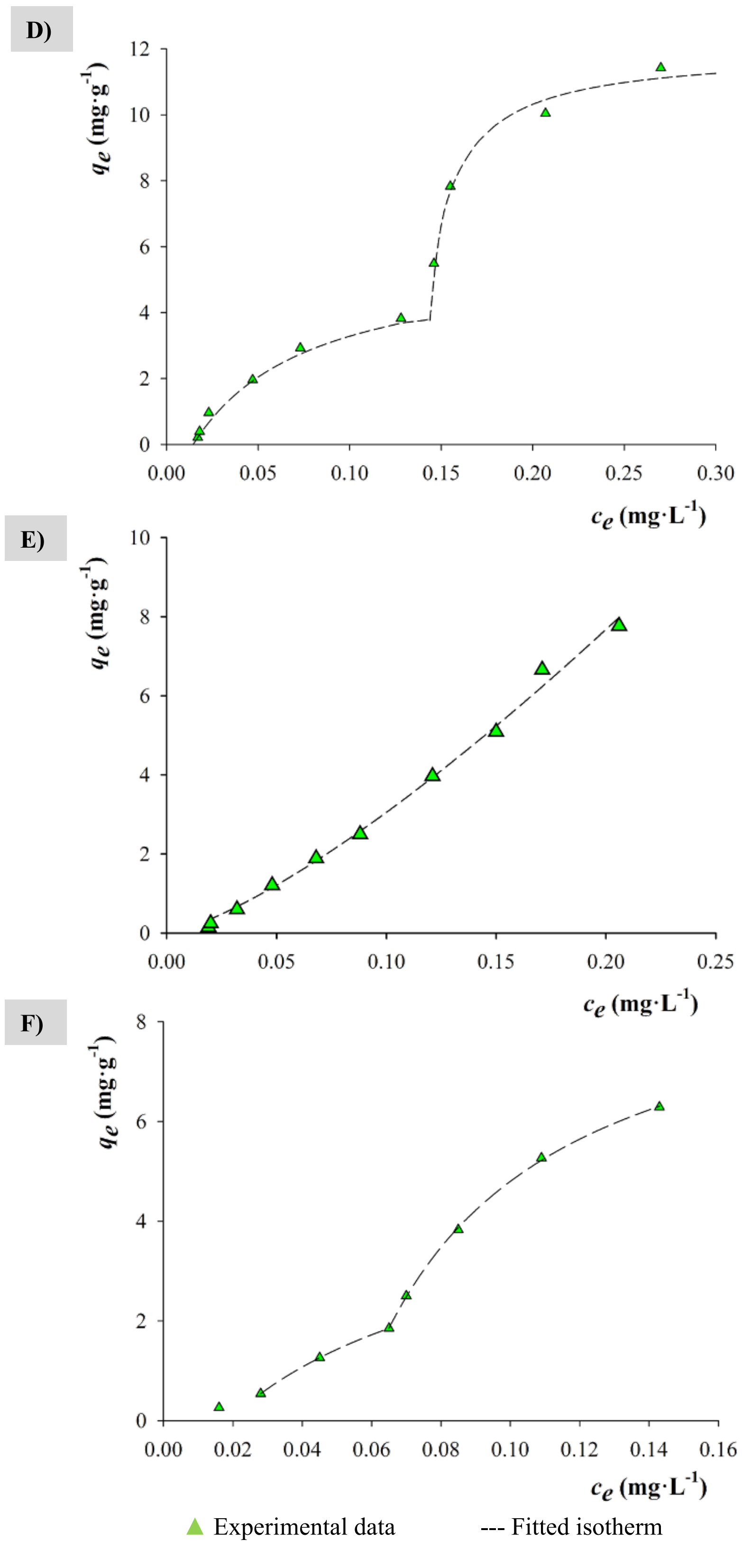

Figure SI.23.(continued)

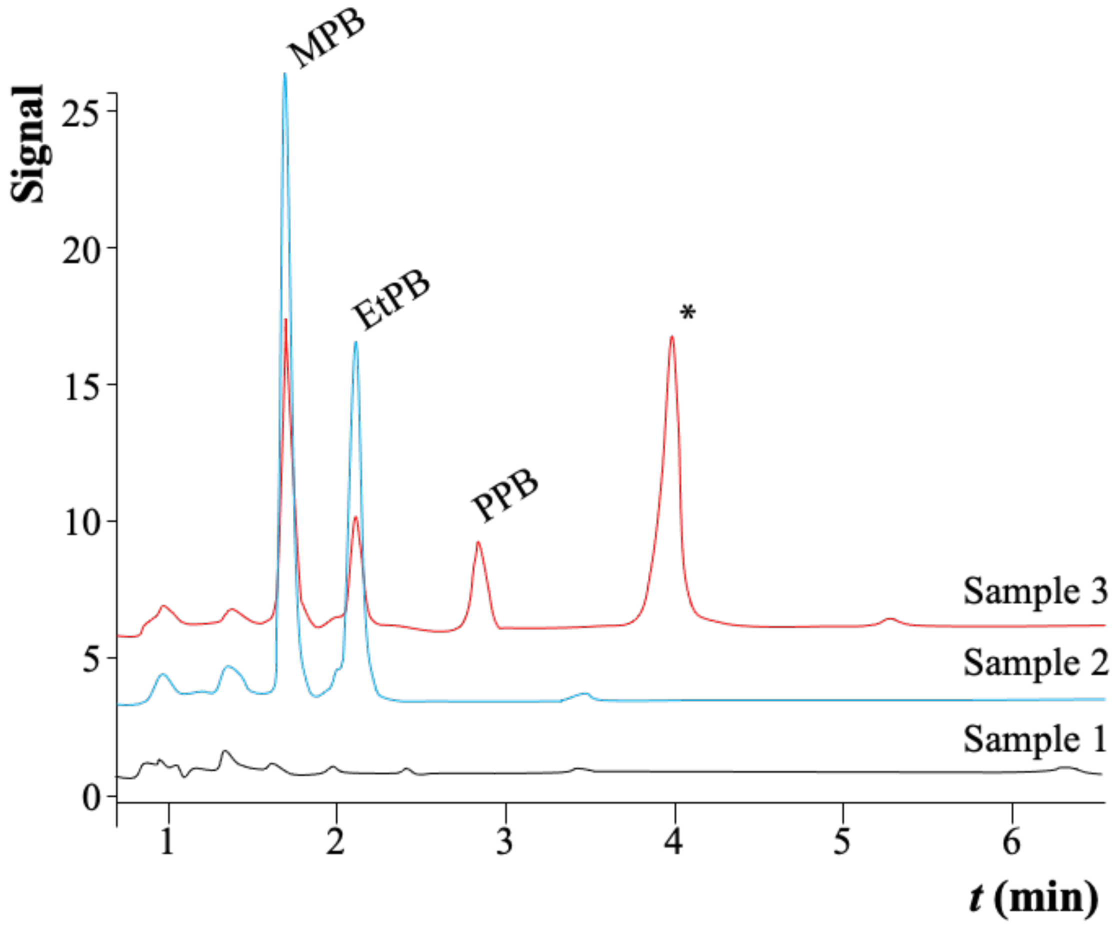

Figure SI.24. Representative chromatograms corresponding to the final extract of the three samples obtained after the MMMs extraction followed by HPLC-UV-Vis determination. *Unknown peak that may be related to BuPB.

Table SI.1. Fitting parameters of the kinetic models for PVDF/UiO-66 MMM.

PCP	Pseudo-first order			Pseudo-second order			Elovich model			Intraparticle diffusion model		
	k_1 (min^{-1})	q_e (mg·g^{-1})	R^2	k_2 (min^{-1})	q_e (mg·g^{-1})	R^2	α (mg·g^{-1}·min^{-1})	B (g·mg^{-1})	R^2	k_p (min^{-1})	C (mg·g^{-1})	R^2
MePB	0.221	0.06	0.552	0.0940	0.06	0.686	0.009	89.3	0.755	0.003	0.02	0.436
BzPB	0.0284	0.1	0.831	0.0572	0.2	0.819	0.08	54.7	0.668	0.01	0.01	0.962
BP	0.0108	0.3	0.874	0.0146	0.3	0.936	0.03	25.1	0.984	0.02	0.02	0.952
BP3	0.0135	0.4	0.963	0.0197	0.4	0.992	0.04	17.5	0.984	0.02	0.01	0.987

Table SI.2. Fitting parameters of the kinetic models for PVDF/UiO-66-COOH MMM.

**PCP**	**Pseudo-first order**			**Pseudo-second order**			**Elovich model**			**Intraparticle diffusion model**		
	k_1 (min^{-1})	q_e ($mg \cdot g^{-1}$)	R^2	k_2 (min^{-1})	q_e ($mg \cdot g^{-1}$)	R^2	α ($mg \cdot g^{-1} \cdot min^{-1}$)	B ($g \cdot mg^{-1}$)	R^2	k_p (min^{-1})	C ($mg \cdot g^{-1}$)	R^2
BP	0.0128	0.3	0.889	0.0172	0.3	0.938	0.03	24.5	0.968	0.02	1.7×10^{-4}	0.960
BP3	0.0199	0.3	0.901	0.0292	0.4	0.960	0.06	19.4	0.986	0.03	0.02	0.938

Table SI.3. Fitting parameters of the kinetic models for PVDF/UiO-67 MMM.

PCP	Pseudo-first order			Pseudo-second order			Elovich model			Intraparticle diffusion model		
	k_1 (min^{-1})	q_e (mg·g^{-1})	R^2	k_2 (min^{-1})	q_e (mg·g^{-1})	R^2	α (mg·g^{-1}·min^{-1})	B (g·mg^{-1})	R^2	k_p (min^{-1})	C (mg·g^{-1})	R^2
MePB	0.185	0.1	0.854	0.457	0.1	0.817	0.8	90.8	0.290	0.008	0.05	0.474
EPB	0.0845	0.1	0.746	0.141	0.1	0.712	0.08	79.8	0.502	0.008	0.02	0.452
BzPB	0.0200	0.5	0.943	0.0323	0.5	0.891	0.08	15.6	0.749	0.04	0.03	0.953
BP	0.0238	0.6	0.976	0.0350	0.6	0.981	0.09	10	0.933	0.05	6.2×10^{-4}	0.968
BP3	0.0156	0.9	0.967	0.0228	1.0	0.987	0.05	71.4	0.967	0.06	0.02	0.981

Table SI.4. Fitting models of the kinetic models for PVDF/DUT-52 MMM.

PCP	Pseudo-first order			Pseudo-second order			Elovich model			Intraparticle diffusion model		
	k_1 (min^{-1})	q_e (mg·g^{-1})	R^2	k_2 (min^{-1})	q_e (mg·g^{-1})	R^2	α (mg·g^{-1}·min^{-1})	B (g·mg^{-1})	R^2	k_p (min^{-1})	C (mg·g^{-1})	R^2
MePB	0.0175	0.06	0.870	0.0288	0.06	0.895	0.004	89.7	0.786	0.004	0.002	0.847
EPB	0.0171	0.08	0.946	0.0278	0.09	0.952	0.009	76.6	0.897	0.006	0.002	0.928
PPB	0.0136	0.1	0.990	0.0198	0.1	0.979	0.01	57.8	0.920	0.006	0.004	0.979
BzPB	0.00460	0.3	0.990	0.00500	0.4	0.984	0.008	18.6	0.887	0.02	0.04	0.853
BP	0.00971	0.3	0.989	0.0126	0.3	0.996	0.02	22.2	0.957	0.02	0.01	0.983
BP3	0.00950	0.3	0.995	0.0124	0.4	0.995	0.02	18.6	0.947	0.02	0.01	0.979

Table SI.5. Fitting parameters of the kinetic models for PVDF/DUT-67 MMM.

**PCP**	**Pseudo-first order**			**Pseudo-second order**			**Intraparticle diffusion model**		
	k_1 (min^{-1})	q_e (mg·g^{-1})	R^2	k_2 (min^{-1})	q_e (mg·g^{-1})	R^2	k_p (min^{-1})	C (mg·g^{-1})	R^2
BP	0.00950	0.004	0.925	0.0126	0.005	0.968	0.0002	0.0002	0.964
BP3	0.0152	0.005	0.963	0.0210	0.006	0.987	0.0004	0.0001	0.982

Table SI.6. Fitting parameters of the kinetic models for PVDF/MOF-801 MMM.

PCP	Pseudo-first order			Pseudo-second order			Intraparticle diffusion model		
	k_1 (min^{-1})	q_e ($mg \cdot g^{-1}$)	R^2	k_2 (min^{-1})	q_e ($mg \cdot g^{-1}$)	R^2	k_p (min^{-1})	C ($mg \cdot g^{-1}$)	R^2
BP	0.00780	0.002	0.901	0.0100	0.002	0.956	0.0001	3.19×10^{-7}	0.959
BP3	0.0155	0.003	0.955	0.0219	0.003	0.985	0.0002	3.29×10^{-5}	0.985

Table SI.7. Fitting parameters of the kinetic models for PVDF/MOF-808 MMM.

PCP	Pseudo-first order			Pseudo-second order			Elovich model			Intraparticle diffusion model		
	k_1 (min^{-1})	q_e (mg·g^{-1})	R^2	k_2 (min^{-1})	q_e (mg·g^{-1})	R^2	α (mg·g^{-1}·min^{-1})	B (g·mg^{-1})	R^2	k_p (min^{-1})	C (mg·g^{-1})	R^2
MePB	0.0027	0.03	0.958	0.0027	0.04	0.956	–	–	–	0.0004	0.002	0.434
BP	0.0096	0.2	0.941	0.012	0.3	0.978	0.02	24.4	0.968	0.03	0.02	0.982
BP3	0.012	0.4	0.988	0.016	0.4	0.997	0.03	15.8	0.962	0.0006	0.002	0.851

Table SI.8. Summary containing the best fitting model for each MOF-MMM and analyte.

PCP	UiO-66	UiO-66-COOH	UiO-67	DUT-52	DUT-67	MOF-801	MOF-808
MePB	E	–	F	S	–	–	
EPB	–	–	F	S	–	–	–
PPB	–	–	–	S / I	–	–	–
BzPB	I	–	I	F	–	–	–
BP	E	E	S	S	S	I	I
BP3	S	E	S	F / S	S	S / I	S

F: pseduo-first order
S: pseudo-second order
E: Elovich model
I: intraparticle diffusion model

Table SI.9. Properties of the six analytes studied. The quantum mechanics kinetic diameter was obtained from Gonzalez-Hernandez *et al.*.[27]

PCP	Molecular formula	Structure	Molecular weight ($g \cdot mol^{-1}$)	pKa	$K_{o/w}$	Quantum mechanics kinetic diameter (Å)	Solubility in water ($g \cdot L^{-1}$)
MePB	$C_8H_8O_3$	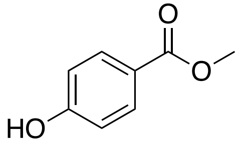	152.15	8.50	1.96	6.50	2.5
EPB	$C_9H_{10}O_3$	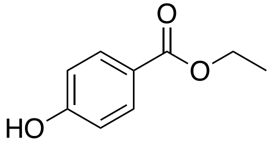	166.17	8.34	2.47	6.50	0.88
PPB	$C_{10}H_{12}O_3$	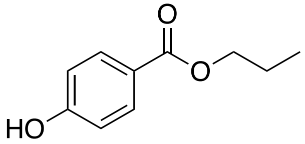	180.20	8.50	3.04	6.50	0.50
BzPB	$C_{14}H_{12}O_3$	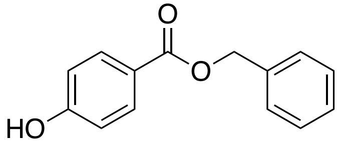	228.24	–	3.56	7.10	0.135
BP	$C_{13}H_{10}O$	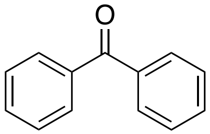	182.22	–	3.18	7.70	0.14
BP3	$C_{14}H_{12}O_3$	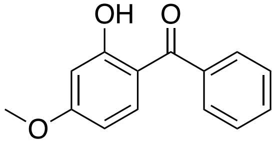	228.24	7.10	3.79	7.99	0.0037

Table SI.10. Uptake (expressed as *q* (mg·g^{-1}) and %) of the six PCPs for UiO-66, UiO-66-COOH, UiO-67, DUT-52, DUT-67, MOF-801, and MOF-808.

PCP	UiO-66		UiO-66-COOH		UiO-67		DUT-52		DUT-67		MOF-801		MOF-808	
	q (mg·g^{-1})	%	*q* (mg·g^{-1})	%	*q* (mg·g^{-1})	%	*q* (mg·g^{-1})	%	*q* (mg·g^{-1})	%	*q* (mg·g^{-1})	%	*q* (mg·g^{-1})	%
MePB	0.07	24	0.04	16	0.07	26	0.08	29	0.01	4	~0.00	2	0.03	10
EPB	0.09	27	0.03	12	0.05	21	0.04	20	0.01	5	0.02	10	~0.00	0
PPB	0.19	66	0.11	48	0.18	79	0.18	78	0.11	53	0.09	46	0.12	52
BzPB	0.29	63	0.12	45	0.14	60	0.18	75	0.05	22	0.02	8	0.04	17
BP	0.21	78	0.15	72	0.17	81	0.14	67	0.09	47	0.11	55	0.15	65
BP3	0.30	86	0.19	79	0.20	85	0.18	78	0.15	71	0.13	66	0.13	70

Table SI.11. Representative studies reported with respect to the adsorption of parabens and UV filters.

Material	amount of material (g)	volume (mL)	BP3 content ($mg \cdot L^{-1}$)	q_e of BP3 ($mg \cdot g^{-1}$)	Ref.
GO[a]	0.0025	4.0	5.0	1.2**	[S2]
PS[b]	0.050	4.0	5.0	0.36**	[S2]
PS[b]-GO[a]	0.050	4.0	5.0	0.40**	[S2]
GAC[c]	0.050	4.0	5.0	0.38**	[S2]
OS_L[d]	0.50	25	2500*	340	[S3]
Lap[e]	0.50	25	2500*	137	[S3]
Mt[f]	0.50	25	2500*	192	[S3]
OS_L[d]	0.50	25	2500*	291	[S4]
Lap[e]	0.50	25	2500*	128	[S4]
Mt[f]	0.50	25	2500*	194	[S4]
PVDF/DUT-52	0.007	20	3.0	11.5	This study

[a]graphene oxide
[b]polysulfone
[c]granular activated carbon
[d]lipophilic organosilicate
[e]laponite
[f]montmorillonite
*ethanolic solution
**value indirectly estimated

Table SI.12. Several analytical quality parameters of the HPLC-DAD method under optimum conditions.

Analyte	Slope ± SD[a]	$R^{2\,b}$	$S_{y/x}$[c]	LOD[d] (µg·L^{-1})	LOQ[e] (µg·L^{-1})	Intra-day RSD[f] (%)	Inter-day RSD[f] (%)
MePB	1490 ± 19	0.999	18807	0.5	1.5	1.5	1.9
EPB	1355 ± 4	0.999	3963	0.5	1.5	0.73	3.5
PPB	1380 ± 6	0.999	5488	0.5	1.5	1.0	1.4
BzPB	943 ± 7	0.999	3619	0.6	1.8	1.7	3.7
BP	1476 ± 5	0.999	5086	0.6	1.8	1.3	4.2
BP3	496 ± 7	0.999	7243	1.0	2.5	3.7	4.8

[a] Standard deviation of the slope
[b] Determination coefficient,
[c] Standard deviation of the residuals (or error of the estimate)
[d] Limits of detection, determined as three times signal to noise ratio
[e] Limits of quantification, determined as ten times signal to noise ratio
[f] Relative standard deviation of the intra-day and inter-day precision, evaluated at a concentration level of 40 µg·L^{-1} (n = 4)

Table SI.13. Relative standard deviation (RSD, in %) of the entire extraction method using the MOF-based MMMs, evaluated at a concentration level of 100 μg·L^{-1} (n = 3).

	**RSD (%)**						
**Analyte**	**PVDF/UiO-66**	**PVDF/UiO-66-COOH**	**PVDF/UiO-67**	**PVDF/DUT-52**	**PVDF/DUT-67**	**PVDF/MOF-801**	**PVDF/MOF-808**
MePB	–	19	23	16	6.7	9.9	5.1
EPB	7.9	18	3.3	19	9.3	8.0	7.7
PPB	13	23	4.5	22	20	8.4	6.0
BzPB	2.3	–	19	6.6	24	9.5	7.7
BP	19	23	2.8	23	2.8	23	21
BP3	6.7	20	14	14	2.8	20	13

Table SI.14. Preconcentration capacity of the seven MOF-based MMMs expressed as enrichment factor (E_F).

PCP	UiO-66	UiO-66-COOH	UiO-67	DUT-52	DUT-67	MOF-801	MOF-808
	E_F[a]	E_F[a]	E_F[a]	E_F[a]	E_F[a]	E_F[a]	E_F[a]
MePB	–	0.8 (0.2)	2.8 (0.9)	8 (1)	1.0 (0.1)	0.9 (0.1)	1.5 (0.1)
EPB	1.6 (0.1)	0.9 (0.3)	9.5 (0.3)	13 (3)	0.9 (0.1)	1.1 (0.1)	1.5 (0.1)
PPB	3.2 (0.4)	1.5 (0.3)	20 (0.9)	19 (4)	1.7 (0.3)	1.7 (0.1)	1.9 (0.1)
BzPB	41 (0.6)	9 (1)	–	32 (2)	9 (2)	7.9 (0.8)	8.0 (0.6)
BP	0.17 (0.09)	0.08 (0.03)	0.76 (0.02)	0.9 (0.1)	0.5 (0.1)	0.5 (0.3)	0.2 (0.2)
BP3	23 (2)	15 (3)	17 (2)	20 (3)	32 (1)	18 (6)	12 (1)

[a] Enrichment factor

Standard deviations are given in brackets

Table SI.15. Extraction efficiency (E_R, in %) of the entire method with the seven MOF-based MMMs, at a MOF loading of 60% (w/w), and HPLC-UV/Vis.

PCP	UiO-66	UiO-66-COOH	UiO-67	DUT-52	DUT-67	MOF-801	MOF-808
	E_R[a] (%)	E_R[a] (%)	E_R[a] (%)	E_R[a] (%)	E_R[a] (%)	E_R[a] (%)	E_R[a] (%)
MePB	–	0.61 (0.3)	2.1 (0.6)	6 (1)	0.7 (0.1)	0.7 (0.1)	1.2 (0.1)
EPB	1.2 (0.1)	0.70 (0.3)	7.2 (0.2)	9 (2)	0.7 (0.1)	0.8 (0.1)	1.1 (0.1)
PPB	2.4 (0.3)	1.12 (0.3)	14.8 (0.7)	14 (2)	1.3 (0.2)	1.2 (0.1)	1.4 (0.1)
BzPB	30.6 (0.4)	3.21 (0.7)	–	23 (2)	7 (1)	5.9 (0.6)	6.0 (0.5)
BP	0.3 (0.1)	0.06 (0.01)	0.6 (0.1)	0.7 (0.2)	0.4 (0.1)	0.4 (0.1)	0.3 (0.1)
BP3	17 (1)	11 (2)	13 (2)	15 (2)	24 (1)	13 (3)	9 (1)

[a] Enrichment factor
Standard deviations are given in brackets

Table SI.16. Estimated limits of detection for the entire methodology using the MOF-based MMMs and HPLC-UV/Vis.

PCP	LOD ($\mu g \cdot L^{-1}$)						
	**UiO-66**	**UiO-66-COOH**	**UiO-67**	**DUT-52**	**DUT-67**	**MOF-801**	**MOF-808**
MePB	–	0.6	0.2	0.06	0.5	0.6	0.3
EPB	0.3	0.6	0.05	0.04	0.6	0.5	0.3
PPB	0.2	0.3	0.03	0.03	0.3	0.3	0.3
BzPB	0.01	0.07	–	0.02	0.07	0.08	0.08
BP	4	8	0.8	0.7	1	1	3
BP3	0.04	0.07	0.06	0.05	0.03	0.06	0.08

References

[S1] Chung, T.-S.; Jiang, L.J.; Li, Y.; Kulprathipanja, S. Mixed matrix membranes (MMMs) comprising organic polymers with dispersed inorganic fillers for gas separation. *Prog. Polym. Sci.* **2007**, *32*, 483–507.

[S2] Rajesha, B.J.; Vishaka, V.H.; Balakrishna, G.R.; Padaki, M.; Nazri, N.A.M. Effective composite membranes of cellulose acetate for removal of benzophenone-3. *J. Water Process. Eng.* **2019**, *30*, 100419.

[S3] Charaabi, S.; Tchara, L.; Marminon, C.; Bouaziz, Z.; Holtzinger, G.; Pensé-Lhéritier, A.-M.; Borgne, M.L.; Issa, S. A comparative adsorption study of benzophenone-3 onto synthesized lipophilic organosilicate, Laponite and montmorillonite. *Appl. Clay Sci.* **2019**, *170*, 114–124.

[S4] Charaabi, S.; Absi, R.; Pensé-Lhéritier, A.-M.; Borgne, M.L.; Issa, S. Adsorption studies of benzophenone-3 onto clay minerals and organosilicates: Kinetics and modelling. *Appl. Clay Sci.* **2021**, *202*, 105937.
